# Synthesis, Antimycobacterial Activity, and Computational Insight of Novel 1,4‐Benzoxazin‐2‐one Derivatives as Promising Candidates against Multidrug‐Resistant *Mycobacterium Tuberculosis*

**DOI:** 10.1002/cmdc.202500073

**Published:** 2025-06-10

**Authors:** Maria Grazia Mamolo, Emanuele Carosati, Diletta Pasin, Alessandro De Logu, Gianluigi Cabiddu, Marko Jukič, Daniele Zampieri

**Affiliations:** ^1^ Department of Chemistry and Pharmaceutical Sciences University of Trieste Via L. Giorgieri 1 34127 Trieste Italy; ^2^ S.O.C. Experimental and Clinical Pharmacology IRCSS RO Aviano Via F. Gallini 2 33081 Aviano Italy; ^3^ Department of Life and Environmental Sciences University of Cagliari Cittadella Universitaria di Monserrato Monserrato 09042 Cagliari Italy; ^4^ Faculty of Chemistry and Chemical Engineering University of Maribor Smetanova ulica 17 SI‐2000 Maribor Slovenia; ^5^ Faculty of Mathematics Natural Sciences and Information Technologies University of Primorska Glagoljaška ulica 8 SI‐6000 Koper Slovenia

**Keywords:** antimycobacterial, benzoxazinones, cytotoxicities, dockings, menaquinone‐B, minimum inhibitory concentrations

## Abstract

In the search for new antitubercular agents, a series of 1,4‐benzoxazinone‐based compounds is designed, synthesized, and evaluated. These molecules show potent antimycobacterial activity, with a minimum inhibitory concentration between 2 and 8 μg mL^−1^. This interesting profile includes activity against several drug‐resistant strains and minimal cytotoxicity against mammalian Vero cells. Structural similarities with analogs from the literature are reinforced by molecular docking and molecular dynamics simulations, suggesting that inhibition of the menaquinone‐B enzyme as a potential mechanism of action. In addition, the active compounds exhibit favorable predicted Absorption, Distribuition, Metabolism, and Excretion (ADME) properties, indicating their potential for oral administration in humans.

## Graphical abstract

A series of 14 novel 1,4‐benzoxazinone derivatives is tested against various strains of *Mycobacterium tuberculosis*. All compounds show high activity against all tested strains, particularly the resistant strains. Additionally, the novel derivatives exhibit low cytotoxicity toward mammalian Vero cells. Favorable ADME profiles are observed and computationally assessed for their interaction with the menaquinone‐B binding site.

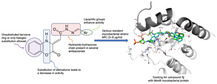

## 1 Introduction

Despite all the challenges, tuberculosis (TB) remains a significant global health problem by 2022. It is the second leading cause of death due to a single infectious disease worldwide, surpassing the impact of HIV/AIDS. Tuberculosis continues to affect millions of people every year, making it critical to address this urgent health crisis quickly and decisively. The United Nations has set a crucial goal of eradicating TB by 2030, which requires unwavering commitment and concerted efforts from all UN member states.^[^ ^1^ ^]^

*Mycobacterium tuberculosis*, discovered by Robert Koch in 1882,^[^ ^2^ ^]^ is the causative agent of TB and continues to claim more lives than any other infectious agent worldwide. The introduction of the Bacillus Calmette–Guérin vaccine and streptomycin ushered in a new era of TB prevention and treatment and dramatically changed the approach to TB control.^[^ ^3^ ^]^ These advances have led to the development of combined treatment approaches, which form the basis of the current six‐month standard treatment regimen.^[^ ^4^ ^]^ Conventional anti‐TB drugs include first‐line drugs such as isoniazid (INH), rifampicin (RIF), pyrazinamide (PYR), and ethambutol (EMB), which are commonly used for newly diagnosed TB.^[^ ^5^ ^]^ When first‐line therapies for TB are ineffective, or the disease acquires drug resistance, second‐line therapies are essential. These alternative drugs are primarily used to manage drug‐resistant forms of TB, such as rifampicin‐resistant TB (RR‐TB), multidrug‐resistant TB (MDR‐TB), and extensively drug‐resistant TB (XDR‐TB). These drugs are categorized into different classes: fluoroquinolones (levofloxacin, gatifloxacin, and others), injectable aminoglycosides (streptomycin, amikacin, kanamycin), antibiotics (cyclosporine, p‐aminosalicylic acid, ethionamide, terizidone), and new drugs like Bedaquiline, Linezolid, and Delamanid.^[^ ^6^ ^]^

Resistant bacterial strains present a significant challenge in the search for novel drug treatments. In fact, numerous mycobacterial enzymes have been identified in recent years. These include alanine–racemase,^[^ ^7^ ^]^ methionine aminopeptidase (MetAP),^[^ ^8^ ^]^ isocitrate lyase (ICL),^[^ ^9^ ^]^ 2‐trans‐enoyl‐acyl carrier reductase (InhA),^[^ ^10^ ^]^ decaprenyl‐phosphoryl‐D‐ribose oxidase (Dpre1),^[^ ^11^ ^]^ effluxpump inhibitors (EPIs),^[^ ^12^ ^]^ and menaquinone‐B enzyme (MenB).^[^ ^13^ ^]^

**Figure 1:**
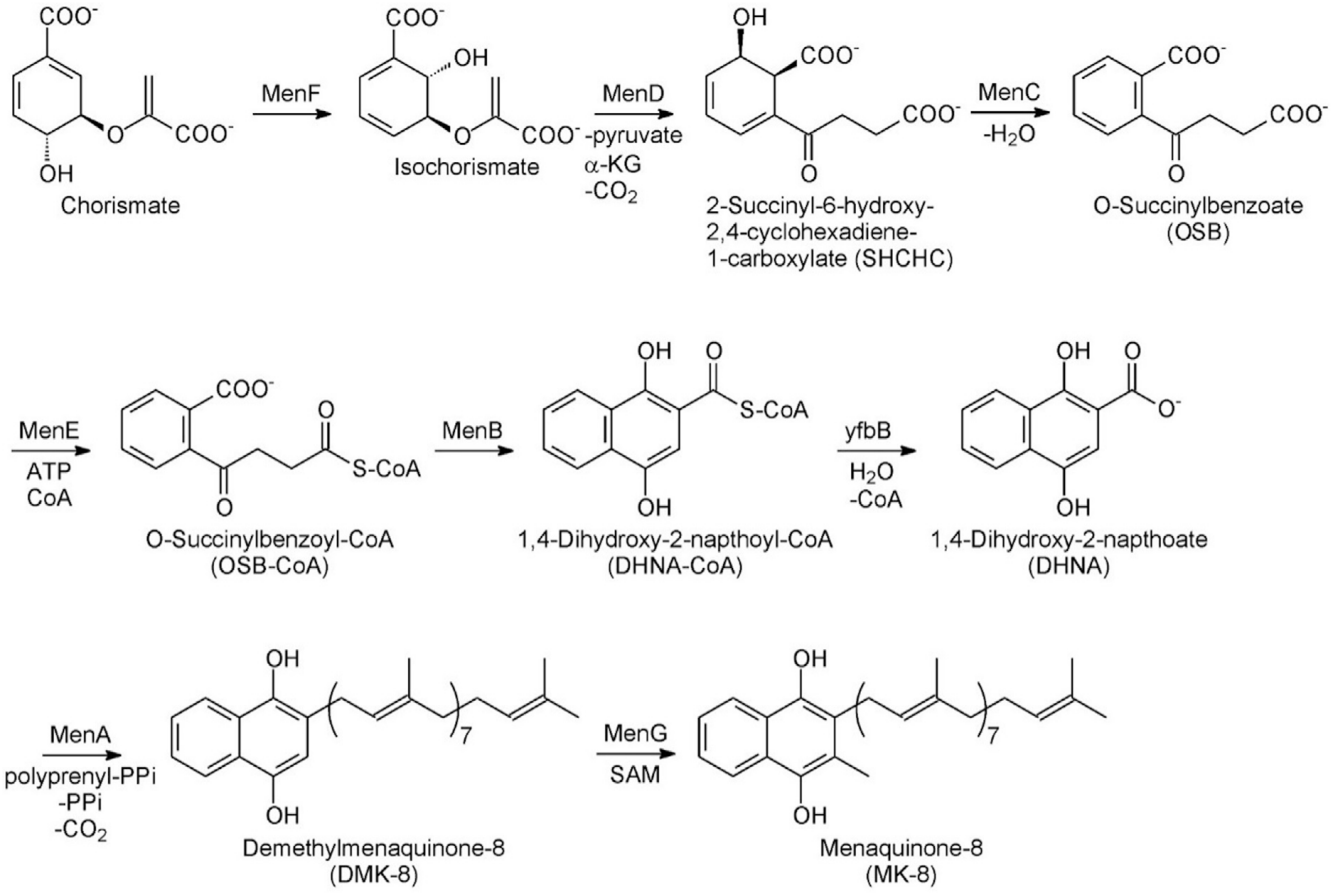
Manaquinone biosynthetic pathway.

In 2010, X. Li and collaborators^[^ ^13^ ^]^ investigated a series of benzoxazinone‐based compounds, targeting the MenB enzyme. This protein is exclusive to bacteria, whereas in humans, this function is mediated by ubiquinone. MenB protein plays a crucial role in preserving the viability of mycobacteria. It converts O‐succinylbenzoyl‐CoA (OSB‐CoA) to 1,4‐dihydroxy‐2‐naphthoyl‐CoA via Claisen condensation in the electron transfer pathway (**Figure** **1**). Therefore, this protein may be a valuable target for the development of new TB treatments.

**Figure 2:**
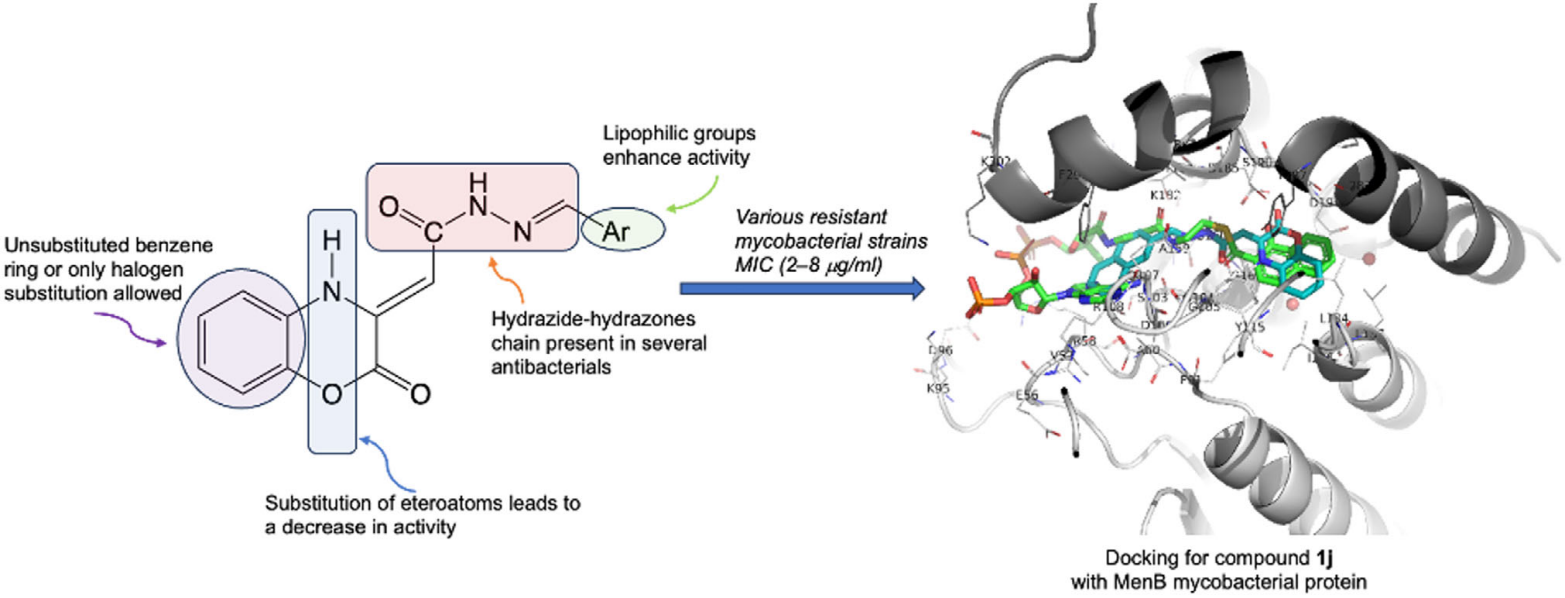
Rational drug design for new 1,4‐benzoxazin‐2‐one derivatives **1a–n**.

In continuation of our search for new benzoxazinone‐based compounds^[^ ^14^ ^]^ with the aim of targeting the MenB enzyme, we planned to synthesize a new series of 1,4‐benzoxazin‐2‐one‐hydrazone derivatives using a hybridization approach. Previous scaffold hopping approaches^[^ ^13^, ^14^ ^]^ did not yield significant improvements in terms of antimycobacterial activity. Moreover, the modification of the substituents on the benzene ring of the benzoxazinone core was limited, as only one halogen atom was tolerated. Based on these assumptions, we decided to leave an unmodified benzoxazinone core. Because many antimycobacterial molecules have been reported to possess a hydrazine, hydrazide, or hydrazone structural moiety,^[^ ^15^, ^16^ ^]^ we inserted a hydrazide–hydrazone linker to connect a secondary (hetero)aromatic group, with the aim of improving the lipophilicity of our compounds, which is crucial for their interaction with the mycobacterial cell wall (**Figure** **2**). The high lipophilicity of the outer waxy layer of mycobacterial cells is one of the main causes of drug development failure in the field of anti‐TB drugs.

## 2 Experimental Design

### 2.1 Chemistry

**Scheme 1:**
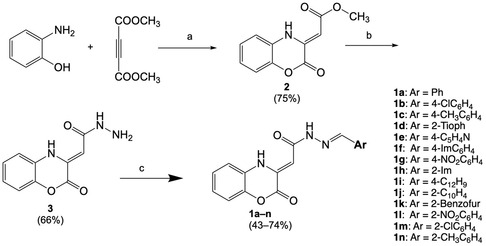
Synthetic route of title compounds **1a–n**. Reagents and conditions: a) abs EtOH, N_2_, reflux; b) H_2_NNH_2_, abs EtOH, reflux; c) appropriate aromatic or heteroaromatic aldehyde, abs EtOH, reflux.

Benzoxazinone derivatives **1a–n** were synthesized in several steps. The process started with 2‐aminophenol, which was combined with dimethyl but‐2‐ynedioate to produce ester intermediate **2** in a high yield. This intermediate was purified by crystallization and then refluxed with an excess of hydrazine hydrate in ethanol to afford carbohydrazide **3**. The final step consisted of the condensation of various aromatic and heterocyclic aldehydes with carbohydrazide, leading to substituted 1,4‐benzoxazinone–hydrazone final compounds **1a–n**. The resulting compounds were isolated as solid materials and analyzed thoroughly. Techniques such as infrared spectroscopy, proton and carbon nuclear magnetic resonance (NMR) spectroscopy, low‐resolution mass spectrometry, and elemental analysis were used to confirm that the structures were consistent with the intended design (**Scheme** **1**).

**Figure 3:**
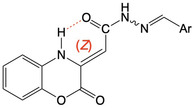
H—bond involved in benzoxazin‐2‐one compounds **1a‐n**.

All the final compounds showed an ^1^H‐NMR spectrum consistent with the presence of a pure *Z*–isomer in the enamine form, which is due to the stabilization of the intramolecular H—bond between the NH group of the oxazine and the exocyclic carbonyl function (**Figure** **3**). From the literature, the signals of the hydrazide and azomethine protons of the *E*−isomers of hydrazido–hydrazone derivatives and hydrazone derivatives appear at lower fields than the corresponding signals of the *Z*–isomers.^[^ ^17^ ^]^ Based on these results, we assigned the *Z*−configuration to the isomers with a higher percentage in the mixtures.

### 2.2 Microbiology

All new 1,4‐benzoxazinone–hydrazone hybrid compounds **1a–n** were tested in vitro for their antimycobacterial activity against *M. tuberculosis* H37Ra (dormant strain), H37Rv (virulent strain), and various resistant strains, as reported in **Table** **1**. For all synthesized compounds **1a–n**, the minimum inhibitory concentration (MIC) was determined, and the corresponding MIC values were expressed as μg mL^−1^ and μM, respectively. We compared the antimycobacterial data obtained with positive reference controls. We used both the 1,4‐benzoxazin‐2‐one compound synthesized by Li et al. (compound 1,^[^ ^13^ ^]^) and by us (intermediate **2**,^[^ ^14^ ^]^), as well as the reference drugs isoniazid (INH), rifampicin (RIF), and streptomycin (STR), which are well‐known anti‐TB agents (Table 1).

**Table 1:** Antimycobacterial activity of the final compounds **1a–n** against several *M. Tuberculosis* strains and cytotoxicity concentration (CC_50_); all the tests were performed in triplicate.

**Cpd**	**Ar**	***M. tuberculosis strain***						**CC**_**50**_ [μ**g mL^−1^]**
		**H37Ra^a)^**	**H37Rv^b)^**	**INH‐R^c)^**	**Rifa‐R^d)^**	**Pyr‐R^e)^**	**Str‐R^f)^**	
		**MIC **μ**g mL^−1^ [**μ**M]**						
**1a**	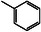	4 (13)	2 (6.5)	8 (26.0)	2 (6.5)	2 (6.5)	4 (13)	>100
**1b**	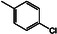	2 (5.8)	2 (5.8)	8 (23.0)	2 (5.8)	2 (5.8)	4 (11.7)	>100
**1c**	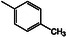	2 (6.2)	2 (6.2)	4 (12.4)	2 (6.2)	2 (6.2)	2 (6.2)	>100
**1d**	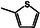	2 (6.4)	2 (6.4)	4 (12.8)	2 (6.4)	2 (6.4)	2 (6.4)	>100
**1e**	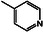	2 (6.5)	2 (6.5)	8 (26.0)	2 (6.5)	2 (6.5)	2 (6.5)	84.14
**1f**	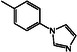	2 (5.3)	2 (5.3)	8 (21.4)	2 (5.3)	2 (5.3)	4 (10.7)	>100
**1g**	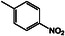	2 (5.7)	2 (5.7)	8 (22.7)	2 (5.7)	2 (5.7)	2 (5.7)	>100
**1h**	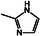	2 (6.7)	2 (6.7)	8 (27.0)	2 (6.7)	2 (6.7)	2 (6.7)	>100
**1i**	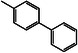	4 (10.4)	2 (5.2)	8 (20.8)	4 (10.4)	2 (5.2)	4 (10.4)	>100
**1j**	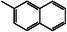	2 (5.6)	2 (5.6)	4 (11.2)	2 (5.6)	2 (5.6)	4 (11.2)	78.26
**1k**	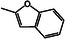	2 (5.7)	4 (11.5)	8 (23.0)	2 (5.7)	2 (5.7)	2 (5.7)	>100
**1l**	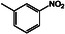	2 (5.7)	2 (5.7)	8 (22.7)	2 (5.7)	2 (5.7)	2 (5.7)	>100
**1m**	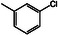	2 (5.8)	2 (5.8)	8 (23.0)	2 (5.8)	2 (5.8)	4 (11.7)	74.82
**1n**	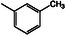	2 (6.2)	2 (6.2)	8 (24.8)	2 (6.2)	2 (6.2)	4 (12.4)	94.35
**2** (Cpd 1)g)	–	4 (18)	1 (4.5)	>64 (>0.3 mM)	2 (9.1)	1 (4.5)	1 (4.5)	>100
INH	–	0.25 (1.8)	0.25 (1.8)	>64 (>0.5 mM)	0.25 (1.8)	0.25 (1.8)	0.25 (1.8)	>1000
RIF	–	0.25 (0.3)	0.5 (0.6)	0.25 (0.3)	>64 (>78)	0.25 (0.3)	0.25 (0.3)	207.03
STR	–	0.5 (0.9)	0.5 (0.9)	0.5 (0.9)	0.5 (0.9)	0.5	>64	>1000

a) *M. tuberculosis* dormant strain ATCC 25 177;

b) *M. tuberculosis* virulent strain ATCC 27 294;

c) Isoniazid‐resistant strain ATCC 35 822;

d) Rifampicin‐resistant strain ATCC 35 838;

e) Pyrazinamide‐resistant strain ATCC 35 828;

f) Streptomycin‐resistant strain ATCC 35 820.

g) From ref. [14].

### 2.3 Cytotoxicity Study

We also investigated the cytotoxicity (CC_50_) of all synthesized compounds **1a–n** against mammalian Vero cells (Table 1), which are commonly used for this type of assay. The results showed that all new molecules exhibited no cytotoxicity even at the highest concentration tested (100 μg mL^−1^). Only four compounds showed minimal cytotoxicity below 100 μg mL^−1^ (corresponding to a concentration range of 200–300 μM). These results indicate that the derivatives have favorable safety profiles.

### 2.4 Computational Study

Currently, there are eight PDB entries available in the RCSB Protein Data Bank [https://www.rcsb.org/ accessed on 20 October 2024] for the *M. tuberculosis* MenB, with the following PDB IDs (in brackets, the corresponding revised PDB accession codes): 1RJM (pdb_00001rjm), 1RJN (pdb_00001rjn), 3T8A (pdb_00003t8a), 3T8B (pdb_00003t8b), 1Q51 (pdb_00001q51), 1Q52 (pdb_00001q52), 4QII (pdb_00004qii) and 4QIJ (pdb_00004qij). After protein alignment and binding site comparison, the chains H and J of the entry 4QIJ^[^ ^18^ ^]^ were chosen for ligand‐target analysis with the ligand 1‐hydroxy‐2‐naphthoyl‐CoA (1‐HNA‐CoA) in a binding pocket (composed of residues from chain J and only partially from chain H). Preliminary analysis was carried out by means of Molecular Interaction Fields from the GRID software,^[^ ^19^, ^20^, ^21^ ^]^ to identify the hotspots in the binding site, as well as PoseView^[^ ^22^, ^23^, ^24^, ^25^ ^]^ to identify the key residues and schematize with 2D representation of the key ligand–target interactions, highlighting hydrogen bonding, hydrophobic, and charge–based interactions. Four conserved water molecules were identified as the crucial components of the binding site (508, 524, 541, and 575). Although none of these directly contributed to the stabilization of the key interactions of the ligand, these water molecules were retained in the receptor structure for subsequent docking studies. Several key residues were identified at the binding site, with a large variety of interactions, including hydrogen bonds (Ser103, Gly105, Gly107, Gly161, Thr184, Asp185, and Ser190), hydrophobic interactions (Trp157 and Ala159), *π*–*π* stacking (Phe299), and charge interactions with the ligand's phosphate groups (Arg58, Lys95, and Lys302).

A subset of derivatives from Li et al.^[^ ^13^ ^]^ who synthesized and tested a set of 1,4‐benzoxazinones, underwent a first run of preliminary docking calculations to verify putative orientations within the 1‐HNA‐CoA binding site. A large difference existed in size among the mentioned derivatives and the ligand from the X‐ray complex, and the docking was questioned on where such small ligands would bind. Limiting the analysis to the first solution of three docked derivatives, which differ in the bicyclic scaffold, one active (compound 1) and two inactive (compounds 12 and 13),^[^ ^13^ ^]^ we observed differences both in the pose and in the associated docking scores. In particular, the unsubstituted derivative with the benzoxazine core was positioned over the naphthyl scaffold of the experimental cocrystallized ligand and had the lowest score value (−7.6 kcal/mol). The inactive derivative with the quinoxaline core (compound 12,^[^ ^13^ ^]^) had the same orientation and comparable predicted score (−7.5 kcal mol^−1^) whereas the one with the benzothiazine core (compound 13,^[^ ^13^ ^]^) had slightly less effective predicted score (‐7.0 kcal mol^−1^) and overlapped to the adenosine core of CoA (data reported as Supporting Information).

**Figure 4:**
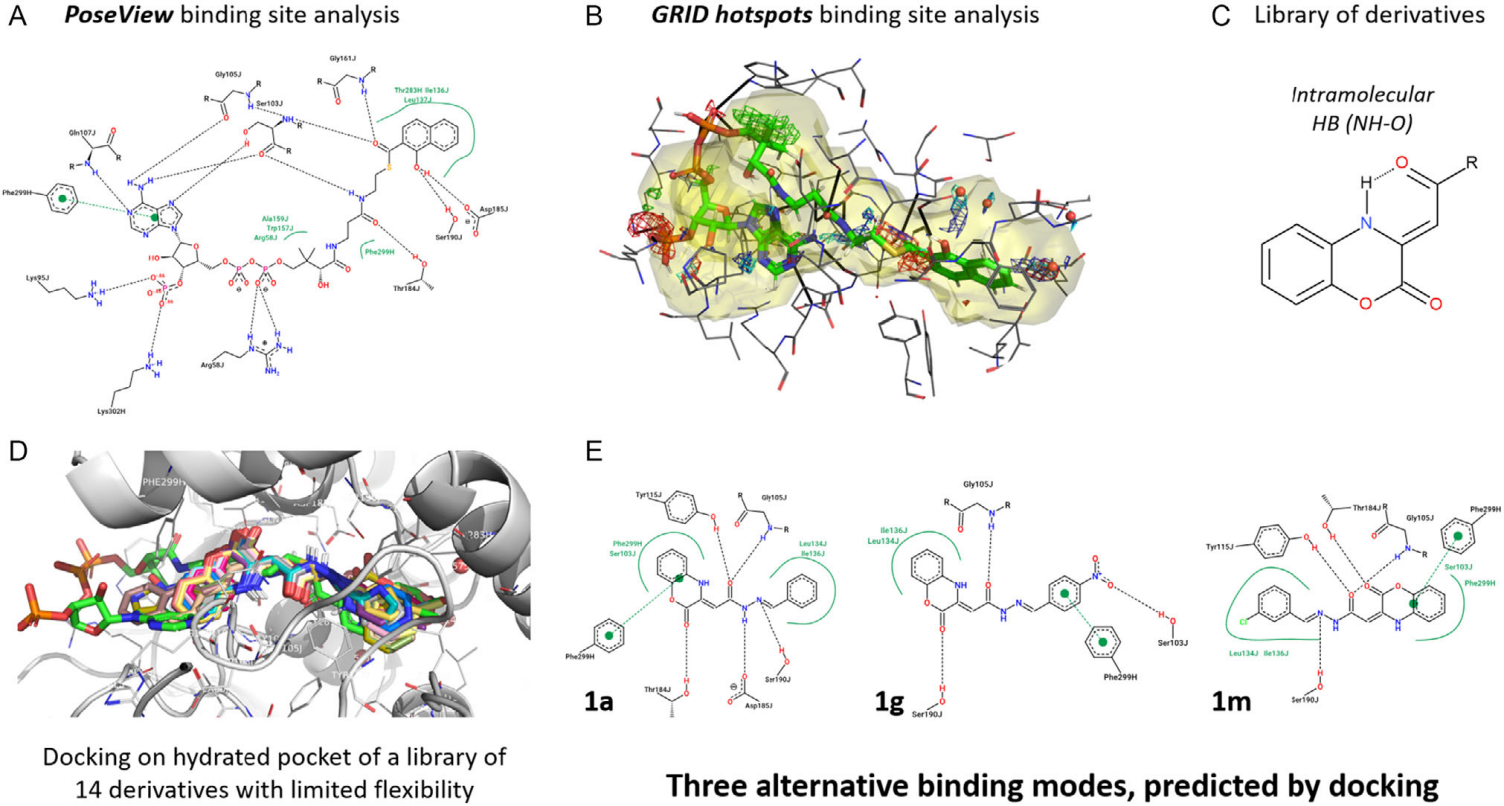
Binding site analysis conducted for the PDB entry 4QIJ (selected chains: J and H) with A) PoseView and B) GRID. C) Peculiarity of the library of ligands is the intramolecular hydrogen bond. For all the compounds, the first docking pose was analyzed; D) their 3D representation is given (differently colored), superposed to the ligand 1‐HNA‐CoA (reported in green). E) 2D images were generated with PoseView using docking poses of three different derivatives (**1a**, **1g** and **1m**), representative for different docking binding modes.

Interestingly, the larger molecular size and tapered shape of the derivatives presented herein (**1a–n**) guaranteed this series of molecules a larger occupancy of the binding site, compared to the smaller benzoxazinone derivatives that initially guided the design. Binding scores (reported as kcal/mol) and docking poses were analyzed to identify key interactions and determine binding modes. The predicted docking scores, reported in **Table** **2**, ranged from −8.0 to −11.0 kcal mol^−1^. For docking poses, we categorized them into two binding modes based on how the molecule occupies different regions of the pocket. When considering only the first pose for each derivative, compounds **1f**, **1g**, **1i,** and **1j** had a common binding mode, which differed from the other derivatives. In all cases, key interactions included hydrogen bonds with residues, including Gly105, Thr184, Asp185, and Ser190, as well as hydrophobic interactions with the polar side chains of Leu134, Leu137, and Phe299. **Figure** **4** shows an extract of the results, including some representative binding modes, whereas we report the first docking pose for all the derivatives in the Supporting Information.

**Table 2:** Predicted score values and key ligand–target interactions for three derivatives from ref. [13] and the new 14 synthesized derivatives **1a–n**.

**Cpd**	**Predicted docking** **score [kcal mol** ^**−1**^ **]**	**Key interactions**	**Fig.^b)^**
Cpd 1c) ^,^ d)	−7.564	Polar: S190(OH) Dry: L134, L137, T283a)	S1
Cpd 12c)	−7.452	Polar: S190(OH) Dry: L134, L137, T283	S2
Cpd 13c)	−6.974	Polar: T184(OH) Dry: F299	S3
**1a** d)	−10.132	Polar: G105(N), T184(OH), D185(COO^−^) Dry: F299, L134, L137	S4
**1b**	−10.023	Polar: T184(OH), S190(OH) Dry: F299, L134, L137	S5
**1c**	−10.170	Polar: G105(N), T184(OH), D185(COO^−^) Dry: F299, L134, L137	S6
**1d**	−9.669	Polar: G105(N), T184(OH), S190(OH) Dry: F299, L134, L137	S7
**1e**	−9.590	Polar: G105(N), T184(OH), S190(OH) Dry: F299, L134, L137	S8
**1f**	−10.117	Polar: G105(N), S190(OH) Dry: F299, L134, L137	S9
**1g** d)	−9.877	Polar: G105(N), S190(OH) Dry: F299, L134, L137	S10
**1h**	−9.631	Polar: G105(N), T184(OH), D185(COO^−^), S190(OH) Dry: F299, L137	S11
**1i**	−11.037	Polar: G105(N), S190(OH) Dry: F299, L134, L137	S12
**1j**	−11.562	Polar: G105(N), S190(OH) Dry: F299, L134, L137	S13
**1k**	−10.909	Polar: G105(N), T184(OH), D185(COO^−^), S190(OH) Dry: F299, L134, L137	S14
**1L**	−10.666	Polar: G105(N), T184(OH), S190(OH), WAT575 Dry: F299, L134, L137	S15
**1m** d)	−10.386	Polar: G105(N); T184(OH, O), D185(COO^−^), S190(OH)	S16
**1n**	−10.689	Polar: G105(N); T184 (OH, O), D185(COO^−^), S190(OH)	S17

a) Key interactions: polar stands for hydrogen bonds, dry stands for hydrophobic interactions (including *π*–*π* interactions);

b) Figures with the first docking pose of each molecule are available as Supporting Information;

c) From ref. [13];

d) Compounds were evaluated by MD as representatives of observed binding modes.

The docking results highlighted the importance of the carboxylic oxygen of the hydrazone linker, which is not only putatively involved in the intramolecular hydrogen bond but is likely to form a hydrogen bond with the NH from Gly105. Although the hydrophobicity of the binding site is low, key interactions occur, including the interaction with Phe299, which is involved in hydrophobic interactions with the two aromatic portions of the ligands, according to their preferred orientations. To simplify the presentation, we only presented and examined the first docking pose for each ligand. However, the second and third docking solutions confirmed the orientations of the ligands on the binding site through the mentioned binding modes, which were schematically reported for three representative molecules. In particular, in the binding mode of a few compounds (**1f**, **1g**, **1i**, and **1j**), the benzoxazine scaffold lies over the naphthyl group of the ligand, thus orienting the larger hydrophobic groups (**1i** and **1j**) or hydrogen bond‐accepting groups (either as heteroatoms on heteroaromatic rings or as nitro groups, such as **1f** and **1g**, respectively) toward the phosphate side of the binding site. In contrast, in the other binding mode, the terminal aromatic moiety overlapped with the naphthyl group of 1‐HNA‐CoA.

The calculated poses via molecular docking for representative systems were also elaborated with molecular dynamics (MD) along up to 250 ns production runs. The system with reference compound 1 from Li et al.^[^ ^13^ ^]^ consisted of three chains, 900 residues, 13 863 protein atoms, 135 Cl, 143 Na, and 49 321 waters, amounting to a total 162 129 atom system. Energy and root mean square deviations (RMSD) were stable along the trajectory, with the all‐atom RMSD of the system below 2.5 Å. The root mean square fluctuation (RMSF) for the reference ligand was measured at 1.42 Å indicating ligand pose stability. The same can also be observed from the ligand contact map along the complete trajectory (see Supporting Information). The system containing compound **1a** was energetically stable during the 250 ns production run. The all‐atom RMSD of the system was again below 2.5 Å. **1a** RMSF was observed at a very stable 0.64 Å with a consistent contact map along the complete trajectory. We could also identify stable alternative conformations with all‐atom average RMSDs at 2.30 and 2.50 Å and ligand RMSFs at 0.95 and 2.37 Å for **1g** and **1m** systems, respectively. Ligand **1m** exhibited a slight repositioning of the terminal meta‐chlorophenyl moiety and however still retained the general binding pose. Energies, as well as the ligand interaction patterns, were stable along the production runs (as can be observed in the supporting information from contact maps and ligand poses).

### 2.5 In Silico Pharmacokinetic Parameters

The novel compounds **1a–n** were predicted for their drug‐likeness properties, using the well‐established Swissadme tool (www.swissadme.ch, accessed on 29 July 2024,^[^ ^26^ ^]^), including the violation of the extended version of Lipinski's rule of five (eRO5).^[^ ^27^ ^]^ Compounds **1a–n** showed promising drug‐like properties, suggesting that they are likely to be well absorbed and effective when administered orally by humans (**Table** **3**). None of the synthesized compounds were expected to cross the blood–brain barrier (BBB), reducing the risk of neurotoxicity at later stages. In addition, none of the derivatives showed any warning of possible pan‐assay interference compounds compounds.

**Table 3:** In silico predicted pharmacokinetic parameters of compounds **1a–n**. Data for reference compound Isoniazid (INH) is reported for comparison.

**Cpd**	**MW**	**eRO5** **Viol.**	**PAINS^a)^**	**clogP^b)^**	**clogS^c)^**	**GI abs**	**BBB Perm**
**1a**	<500	≤1	–	≤5	≤5	–	–
	307.30	0	No	2.30	−3.67	High	No
**1b**	341.75	0	No	2.83	−4.25	High	No
**1c**	321.33	0	No	2.65	−3.96	High	No
**1d**	313.33	0	No	2.34	−3.71	High	No
**1e**	308.29	0	No	1.57	−3,00	High	No
**1f**	373.36	0	No	2.05	−3.89	High	No
**1g**	352.30	0	No	1.56	−3.71	High	No
**1h**	297.27	0	No	0.85	−2.64	High	No
**1i**	383.40	0	No	3.66	−5.14	High	No
**1j**	357.36	0	No	3.21	−4.80	High	No
**1k**	347.32	0	No	2.63	−4.41	High	No
**1L**	352.30	0	No	1.53	−3.71	High	No
**1m**	341.75	0	No	2.85	−4.25	High	No
**1n**	321.33	0	No	2.63	−3.96	High	No
INH	137.14	0	No	−0.35	−0.56	High	No

a) Implemented from J. B. Bell and G. A. Holloway, J. Med. Chem. 2010;

b) calculated partition coefficient [C_octanol_]/[C_water_];

c) calculated water solubility (mol L^−1^)_water_.

## 3 Results and Discussion

Upon reviewing public databases for bioactivity data on MenB, we found that the available data were limited. Specifically, in the ChEMBL database, the target 1,4‐Dihydroxy‐2‐naphthoyl‐CoA synthase (ChEMBL ID: CHEMBL1275214, UniProt ID: P9WNP5) was associated with only 11 binding records under the “associated assays” category, derived from two publications,^[^ ^13^, ^28^ ^]^ both from the same research group. Similarly, under the “associated bioactivities” category, 69 records, comprising 12 K_*i*_ values, 37 IC_50_ values, and 20 inhibition data points, also originate from these papers. This scarcity of data underscores the challenges in developing specific assays for MenB, yet it reinforced our decision to consider MenB as a putative target owing to its shared scaffold.

All newly synthesized 1,4‐benzoxazinone–hydrazone compounds **1a–n** were tested for their antimycobacterial activity against different *M. tuberculosis* strains and evaluated for potential cytotoxicity (Table 1). The results showed high antitubercular activity against all the strains tested, with values in the range of 2–8 μg mL^−1^. Notably, this new series of molecules displayed potent inhibition against INH‐resistant strain, with MIC values in the range of 4–8 μg mL^−1^ (reference, INH: >64 μg mL^−1^). Neither substitution on the phenyl ring nor the presence of a heterocyclic nucleus substantially affect the antimycobacterial potency, as it remained largely unchanged across the examined compounds. Note that our new series of benzoxazinone derivatives is only slightly less active against the H37Rv strain than the lead compound discovered by Li et al.^[^ ^13^ ^]^ when considering the MIC data in micromolar terms. In fact, their lead compound reached MIC value of 0.6 μg ml^−1^, corresponding to 2.7 μM, slightly lower than our best result of 5.2 μM (compound **1i**). A further comparison can be made with compound 1 from Li et al. as well as with the benzoxazinone derivatives previously synthesized by Li^[^ ^13^ ^]^ and our group,^[^ ^14^ ^]^ where the MIC values were comparable for almost all the tested strains, except for the activity against the INH‐resistant strain, where they proved to be inactive. The latter is one of the most important data points obtained from our new molecules. This flattened profile could be due to the spatial distribution of the key pharmacophores in the series, with two lipophilic portions at the two extremities. Through docking simulations, we hypothesized binding modes with contributions from two hydrophobic regions, one close to Phe299 and another in proximity to Leu134 and Leu137, and polar interactions, with several residues interacting with the oxygen atoms of the benzoxazinone core as well as the nitrogen atoms of the hydrazone group. Preliminary validation of such modes was performed via MD simulations; however, further validation may arise through the design, synthesis, and testing of new derivatives.

The newly synthesized compounds **1a–n** were subjected to cytotoxicity evaluation using an assay against mammalian Vero cells. The findings revealed that none of the molecules exhibited cytotoxicity at the highest dose examined (100 μg mL^−1^), except for four derivatives that demonstrated values almost reaching the uppermost level.

Compounds **1a–n** were evaluated in silico for their physiochemical parameters (ADME), and the results showed that they possess good drug‐likeness properties and can be orally administered in humans. None of the synthesized molecules could penetrate the BBB, thus avoiding possible side effects in the central nervous system.

## 4 Conclusions

In this study, we describe the synthesis of a new series of benzoxazin‐2‐one‐hydrazone derivatives through a hybridization approach and evaluate their antimycobacterial activity.

The synthesized compounds **1a–n** showed effective antimicrobial activity, with MIC of 2 μg mL^−1^ against both the attenuated (H37Ra) and the virulent (H37Rv) strains of *M. tuberculosis*. Furthermore, these compounds exhibited a MIC range of 2−8 μg mL^−1^ against various resistant strains and demonstrated particularly high inhibitory activity against an isoniazid–resistant strain, outperforming the INH reference drug (MIC >64 μg mL^−1^). Importantly, they did not display significant cytotoxicity toward mammalian Vero cells, indicating good selectivity for mycobacteria.

The antimycobacterial profiles of the derivatives suggest that they are all effective, although chemical diversity suggests different binding modes. Thus, we extended the in silico analysis through docking calculations and molecular dynamics simulations, confirming that the molecules of the series could occupy the cavity region of the naphthyl terminal of MenB through the benzoxazin‐2‐one scaffold or with a substituted aryl moiety.

The novel derivatives outperformed literature compounds in predicted binding affinities, with observed interactions aligned with key binding site features. Further experimental validation of these derivatives as potential 1‐HNA‐CoA synthase inhibitors is warranted; thus, the design of new derivatives is ongoing to strengthen the structure–activity relationship of this interesting series of analogs.

## 5 Experimental Section

5.1

5.1.1

##### Chemistry: General Chemistry

The chemicals used in the experiments were of reagent grade and were used as supplied. Thin‐layer chromatography (TLC‐GF_254_) on silica gel plates containing a 254 nm fluorescent indicator (Merck Life Science S.r.l., Milan, Italy) was routinely used to monitor the progress of the reactions and the composition of the product mixtures. Melting points were measured using a Stuart SMP300 instrument (Cole–Palmer UK, Cambridgeshire, UK), and the values reported were uncorrected. Infrared spectra were recorded using a Jasco 4700 FTIR spectrometer (Fourier Transform Infrared Spectroscopy [JASCO], Lexington, KY, USA) equipped with an ATR device. NMR spectra were recorded using a Varian 400 MHz instrument (Agilent, Santa Clara, CA, USA), with the ^1^H‐NMR and ^13^C‐NMR signals referenced to tetramethyl silane as the internal standard. A drop of D_2_O was added to facilitate the assignment of the NH protons. The coupling constants (*J*) are given in Hz, and the abbreviations for the splitting are s, singlet; d, doublet; dd, doublet of doublets; dd, doublet of doublet of doublets; t, triplet; dt, doublet of triplets; td, triplet of doublets; q, quartet; m, multiplet; br, broad. Chemical shifts are reported as *δ* (ppm) in DMSOease‐*d6*. Elemental analyzes (C, H, N) were carried out using an Elemental Vario ELIII apparatus (Elementar Italia srl, Lomazzo, Como, Italy), and the results agreed with the theoretical values within a ±0.4% tolerance. The substance was analyzed using a Thermo Fisher Ion Trap LTQ XL spectrometer (Thermo Fisher Scientific Inc., Waltham, Massachusetts, USA). This was done by steadily feeding a methanol solution of the sample into the instrument (the methanol was of High‐purPerformance Liquid Cromatography grade). Compounds were named following IUPAC rules as applied by ChemDraw Professional 16.0 software (PerkinElmer Informatics, Inc., USA).

##### General Procedure for the Synthesis of Intermediates **2** and **3** (NMR spectra are reported in the Supporting Information): (Z)‐Methyl‐2‐(2‐oxo‐2H‐benzo[b][1,4]oxazin‐3(4H)‐ylidene)acetate **2**

The 2‐aminophenol (4.0 g, 36.7 mmol) was dissolved in absolute ethanol (50 mL) under a nitrogen atmosphere. Then, an equimolar amount of dimethyl but‐2‐ynedioate was added to the stirred solution. After a short time, a yellow solid began to form. Few hours later, the mixture was filtered under vacuum, and the yellow solid was washed with cold ethanol, resulting in a pure and chromatographically clean product.

Yield: 75%. M.p. 164–168 °C (164–165 °C^[^ ^29^ ^]^). I.R. (ATR, cm^−1^) 3631, 1754, 1698, 1635; MS (ESI^−^): *m/z* 218 [*M*‐H]^−^. elemental analysis calcd (%) for C_11_H_9_NO_4_: C 60.14, H 4.14, N 6.39; found: C 60.20, H 4.30, N 6.50. ^1^H NMR (400 MHz, DMSO‐*d6*) d 10.66 (s, 1H, NH, cyc.), 7.50 (dd, *J* = 8.0, 1.5 Hz, 1H, arom.), 7.21 – 7.09 (m, 2H, arom.), 7.02 (ddd, *J* = 8.1, 7.4, 1.5 Hz, 1H, arom.), 5.60 (s, 1H, C=CH), 3.69 (s, 3H, OCH_3_). ^13^C NMR (101 MHz, DMSO‐*d6*) d 168.98, 156.37, 140.33, 139.22, 125.62, 125.05, 122.90, 116.71, 116.46, 88.68, 51.51.

##### (Z)‐2‐(2‐oxo‐2H‐benzo[b][1,4]oxazin‐3(4H)‐ylidene)acetohydrazide **3**

2.5 grams (8.58 mmol) of the initial compound **2** were dissolved in 50 mL of hot, pure ethanol. Then, 830 microliters (12.9 mmol) of hydrazine hydrate, diluted with 2 mL of pure ethanol, were added. The mixture was refluxed and stirred for 3 h. When the reaction finished, a dark‐yellow solid precipitated, which was filtered and recrystallized using pure ethanol.

Yield: 66%. M.p. 178–182 °C. I.R. (ATR, cm^−1^): 3308, 3247, 3177, 1679, and 1635. MS (ESI^−^): *m/z* 218 [*M*‐H]^−^. ^1^H NMR (400 MHz, DMSO‐*d6*) *δ* 10.30 (s, 1H, NH cyc.), 8.18 (s, 1H, NH), 7.27 (dd, *J* = 7.9, 1.5 Hz, 1H, arom.), 7.02–6.93 (m, 1H, arom.), 6.91 (dd, *J* = 8.0, 1.6 Hz, 1H, arom.), 6.87–6.79 (m, 1H, arom.), 5.43 (s, 1H, C=CH), 4.66 (s, 2H, NH_2_). ^13^C NMR (101 MHz, DMSO‐*d6*) *δ* 172.32, 166.95, 148.24, 141.71, 126.81, 125.31, 120.16, 119.41, 115.74, 86.37.

##### General Procedure for the Synthesis of Final Compounds **1a–n** (NMR spectra are reported in the Supporting Information)

A quantity of 0.1 grams (0.45 mmol) of compound **3** was dissolved in warm ethanol (30 mL), while being stirred. Gradually, 1 equivalent of the aromatic aldehyde, previously diluted with a small amount of ethanol, was added.

The mixture was then heated to reflux and stirred for 3–4 h (as monitored by TLC using a solvent system of 9.5 parts CH_2_Cl_2_ to 0.5 parts EtOH). Afterward, the solution was left to allow the product to precipitate in a glass evaporation dish. The solid residue was collected by filtration under reduced pressure, yielding a pure, colored solid.

##### (Z)‐N′‐((E)‐benzylidene)‐2‐(2‐oxo‐2H‐benzo[b][1,4]oxazin‐3(4H)‐ylidene)acetohydrazide **1a**

Orangy–yellow solid. Yield: 44%. M.p. 152–155 °C. I.R. (ATR, cm^−1^) 3343, 1748, 1696, 1637, 1590. MS (ESI^−^): *m/z* 308 [M+H]^+^. ^1^H NMR (400 MHz, DMSO‐*d6*) *δ* 10.32 (s, 1H, NH–N), 9.15 (s, 1H, N=CH), 8.55 (s, 1H, NH cyc.), 7.85–7.77 (m, 2H, arom.), 7.50 (tq, *J* = 5.9, 2.1 Hz, 4 H, arom.), 7.32 (dd, *J* = 7.9, 1.5 Hz, 1H, arom.), 7.03 (ddd, *J* = 8.1, 7.3, 1.5 Hz, 1 H, arom.), 6.94 (dd, *J* = 8.1, 1.5 Hz, 1H, arom.), 6.86 (td, *J* = 7.6, 1.5 Hz, 1H, arom.), 5.55 (s, 1H, C=CH).^13^C NMR (101 MHz, DMSO‐*d6*) *δ* 169.54, 164.62, 157.01, 148.80, 142.77, 134.14, 129.71, 129.42, 128.14, 126.33, 126.08, 120.58, 120.19, 116.05, 87.35. Elemental analysis calcd (%) for C_17_H_13_N_3_O_3_: C 66.44, H 4.26, and N 13.67; found: C 66.70, H 4.25, and N 13.30.

##### (Z)‐N′‐((E)‐4‐chlorobenzylidene)‐2‐(2‐oxo‐2H‐benzo[b][1,4]oxazin‐3(4H)‐ylidene)acetohydrazide **1b**

Light–orange solid. Yield: 46%. M.p. 213–216 °C. I.R. (ATR, cm^−1^) 3335, 1753, 1694, 1633, 1587; MS (ESI^−^). *m/z* 340 [*M*‐H]^−^, 342 [M+H]^+^. ^1^H NMR (400 MHz, DMSO‐*d6*) *δ* 10.31 (s, 1H, NH–N), 9.19 (s, 1H, N=CH), 8.58 (s, 1H, NH cyc.), 7.90–7.81 (m, 2H, arom.), 7.59–7.52 (m, 2H, arom.), 7.32 (dd, *J* = 8.0, 1.5 Hz, 1H, arom.), 7.08–6.98 (m, 1H, arom.), 6.94 (dd, *J* = 8.1, 1.4 Hz, 1H, arom.), 6.90–6.82 (m, 1H, arom.), 5.55 (s, 1H, C=CH). ^13^C NMR (101 MHz, DMSO‐*d6*) *δ* 169.44, 155.10, 148.84, 142.84, 136.26, 133.15, 129.76, 129.55, 126.28, 126.14, 120.69, 120.18, 116.07, 87.42. Elemental analysis calcd (%) for C_17_H_12_N_3_O_3_Cl: C 59.75, H 3.54, and N 12.30; found: C 59.60, H 3.25, and N 12.00.

##### (Z)‐N′‐((E)‐4‐methylbenzylidene)‐2‐(2‐oxo‐2H‐benzo[b][1,4]oxazin‐3(4H)‐ylidene)acetohydrazide **1c**

Yellow solid. Yield: 55%. M.p. 222–225 °C. I.R. (ATR, cm^−1^) 3341, 1753, 1695, 1639, 1588. MS (ESI^−^): *m/z* 320 [*M*‐H]^−^. ^1^H NMR (400 MHz, DMSO‐*d6*) δ 10.34 (s, 1H, NH–N), 9.08 (s, 1H, N=CH), 8.54 (s, 1H, NH cyc.), 7.74–7.67 (m, 2H, arom.), 7.35–7.27 (m, 3H, arom.), 7.03 (td, *J* = 7.6, 1.5 Hz, 1H, arom.), 6.94 (dd, *J* = 8.1, 1.5 Hz, 1H, arom.), 6.86 (td, *J *= 7.6, 1.5 Hz, 1H, arom.), 5.55 (s, 1H, C=CH), 2.36 (s, 3H, CH_3_). ^13^C NMR (101 MHz, DMSO‐*d6*) *δ* 169.60, 164.64, 157.39, 148.83, 142.70, 141.88, 131.42, 130.03, 128.17, 126.37, 126.04, 120.48, 120.13, 116.03, 87.26, 21.59. Elemental analysis calcd (%) for C_18_H_15_N_3_O_3_: C 67.28, H 4.71, and N 13.08; found: C 67.50, H 4.65, and N 13.20.

##### (Z)‐2‐(2‐oxo‐2H‐benzo[b][1,4]oxazin‐3(4H)‐ylidene)‐N′‐((E)‐thiophen‐2‐ylmethylene)acetohydrazide **1d**

Orange solid. Yield: 57%. M.p. 180–183 °C. I.R. (ATR, cm^−1^) 3337, 1750, 1692, 1635, 1588. MS (ESI^−^): *m/z* 312 [*M*‐H]^−^. ^1^H NMR (400 MHz, DMSO‐*d6*) *δ* 10.31 (s, 1H, NH–N), 9.29 (s, 1H, N=CH), 8.54 (s, 1H, NH cyc.), 7.78 (dd, *J* = 5.0, 1.1 Hz, 1H, arom.), 7.65 (dd, *J* = 3.8, 1.2 Hz, 1H, arom.), 7.31 (dd, *J* = 7.9, 1.5 Hz, 1H, arom.), 7.19 (dd, *J* = 5.0, 3.6 Hz, 1H, arom.), 7.03 (td, *J* = 7.7, 1.5 Hz, 1H, arom.), 6.94 (dd, *J* = 8.1, 1.5 Hz, 1H, arom.), 6.86 (td, *J* = 7.6, 1.5 Hz, 1H, arom.), 5.53 (s, 1H, C=CH). ^13^C NMR (101 MHz, DMSO‐*d6*) *δ* 169.49, 164.46, 151.89, 148.80, 142.82, 138.76, 134.07, 131.13, 128.63, 126.30, 126.10, 120.61, 120.19, 116.05. Elemental analysis calcd (%) for C_15_H_11_N_3_O_3_S: C 57.50, H 3.54, and N 13.41; found: C 57.25, H 3.75, and N 13.20.

##### (Z)‐2‐(2‐oxo‐2H‐benzo[b][1,4]oxazin‐3(4H)‐ylidene)‐N′‐((E)‐pyridin‐4‐ylmethylene)acetohydrazide **1e**

Orange solid. Yield: 50%. M.p. 210–213 °C. I.R. (ATR, cm^−1^) 3344, 1755, 1704, 1641, 1592. MS (ESI^−^): *m/z* 307 [*M*‐H]^−^. ^1^H NMR (400 MHz, DMSO‐*d6*) *δ* 10.31 (s, 1H, NH–N), 9.29 (s, 1H, N=CH), 8.69 (d, *J* = 5.0 Hz, 2 H, arom.), 8.64 (s, 1H, NH cyc.), 7.74 (d, *J* = 5.2 Hz, 2H, arom.), 7.32 (d, *J* = 7.9 Hz, 1H, arom.), 7.04 (t, *J *= 7.7 Hz, 1H, arom.), 6.94 (d, *J* = 8.1 Hz, 1H, arom.), 6.86 (t, *J* = 7.7 Hz, 1H, arom.), 5.56 (s, 1H, C=CH). ^13^C NMR (101 MHz, DMSO‐*d6*) *δ* 169.27, 164.50, 152.68, 150.91, 148.97, 143.06, 141.51, 126.30, 126.16, 121.72, 120.93, 120.17, 116.14, 87.72. Elemental analysis calcd (%) for C_16_H_12_N_4_O_3_: C 62.33, H 3.92, and N 18.17; found: C 62.20.25, H 3.80, and N 17.90.

##### (Z)‐N′‐((E)‐4‐(1H‐imidazol‐1‐yl)benzylidene)‐2‐(2‐oxo‐2H‐benzo[b][1,4]oxazin‐3(4H)‐ylidene)acetohydrazide **1f**

Orange solid. Yield: 55%. M.p. 119–222 °C. I.R. (ATR, cm^−1^) 3340, 1757, 1707, 1639, 1592. MS (ESI^−^): *m/z* 374 [M+H]^+^. ^1^H NMR (400 MHz, DMSO‐*d6*) *δ* 10.33 (s, 1H, NH–N), 9.21 (s, 1H, N=CH), 8.57 (s, 1H, NH cyc.), 8.38 (t, *J* = 1.2 Hz, 1H, arom.), 7.99–7.90 (m, 2 H, arom.), 7.85 (t, *J* = 1.4 Hz, 1H, arom.), 7.85–7.77 (m, 2 H, arom.), 7.33 (dd, *J* = 7.9, 1.5 Hz, 1H, arom.), 7.13 (t, *J* = 1.2 Hz, 1H, arom.), 7.04 (td, *J* = 7.7, 1.5 Hz, 1H, arom.), 6.95 (dd, *J* = 8.1, 1.5 Hz, 1H, arom.), 6.86 (td, *J* = 7.6, 1.5 Hz, 1H, arom.), 5.56 (s, 1H, C=CH). ^13^C NMR (101 MHz, DMSO‐*d6*) *δ* 169.52, 164.61, 155.63, 148.82, 142.82, 139.24, 136.04, 132.48, 130.67, 129.67, 126.30, 126.13, 120.76, 120.64, 120.20, 118.23, 116.06, 87.39. Elemental analysis calcd (%) for C_20_H_15_N_5_O_3_: C 64.34, H 4.05, and N 18.76; found: C 64.50, H 4.00, and N 18.90.

##### (Z)‐N′‐((E)‐4‐nitrobenzylidene)‐2‐(2‐oxo‐2H‐benzo[b][1,4]oxazin‐3(4H)‐ylidene)acetohydrazide **1g**

Yellow solid. Yield: 74%. M.p. 237–240 °C. I.R. (ATR, cm^−1^) 3328, 1731, 1639, 1611, 1591. MS (ESI^−^): *m/z* 351 [*M*‐H]^−^. ^1^H NMR (400 MHz, DMSO‐*d6*) *δ* 10.31 (s, 1H, NH–N), 9.39 (s, 1H, N=CH), 8.64 (s, 1H, NH cyc.), 8.35–8.21 (m, 2H, arom.), 8.15–8.03 (m, 2 H, arom.), 7.32 (dd, *J* = 8.0, 1.5 Hz, 1H, arom.), 7.04 (td, *J* = 7.7, 1.5 Hz, 1H, arom.), 6.94 (dd, *J* = 8.0, 1.5 Hz, 1H, arom.), 6.86 (td, *J* = 7.6, 1.5 Hz, 1H, arom.), 5.56 (s, 1H, C=CH). ^13^C NMR (101 MHz, DMSO‐*d6*) *δ* 169.27, 164.49, 152.57, 149.04, 148.95, 143.04, 140.47, 129.04, 126.29, 126.16, 124.55, 120.90, 120.17, 116.13, 87.70. Elemental analysis calcd (%) for C_17_H_12_N_4_O_5_: C 57.96, H 3.43, and N 15.90; found: C 58.10, H 3.55, and N 15.95.

##### (Z)‐N′‐((E)‐(1H‐imidazol‐2‐yl)methylene)‐2‐(2‐oxo‐2H‐benzo[b][1,4]oxazin‐3(4H)‐ylidene)acetohydrazide1H **1h**

Dark‐yellow solid. Yield: 45%. M.p. 237–238 °C. I.R. (ATR, cm^−1^) 3326, 1760, 1711, 1637, 1593. MS (ESI^−^): *m/z* 296 [*M*‐H]^−^. ^1^H NMR (400 MHz, DMSO‐*d6*) *δ* 13.01 (s, 1H, NH pirr.), 10.32 (s, 1H, NH–N), 8.99 (s, 1H,, N=CH), 8.59 (s, 1H, NH cyc.), 7.31 (d, *J* = 7.8 Hz, 2H, arom.), 7.17 (d, *J* = 8.9 Hz, 1H, arom.), 7.03 (t, *J* = 7.7 Hz, 1H, arom.), 6.94 (d, *J* = 8.0 Hz, 1H, arom.), 6.86 (t, *J* = 7.7 Hz, 1H, arom.), 5.53 (s, 1H, C=CH). ^13^C NMR (101 MHz, DMSO‐*d6*) *δ* 169.43, 164.47, 148.89, 147.86, 142.94, 142.31, 126.25, 126.19, 120.76, 120.19, 116.10, 87.30. Elemental analysis calcd (%) for C_14_H_11_N_5_O_3_: C 56.57, H 3.73, and N 23.56; found: C 56.10, H 3.52, and N 23.25.

##### (Z)‐N′‐((E)‐[1,1′‐biphenyl]‐4‐ylmethylene)‐2‐(2‐oxo‐2H‐benzo[b][1,4]oxazin‐3(4H)‐ylidene)acetohydrazide **1i**

Light‐orange solid. Yield: 73%. M.p. 224–226 °C. I.R. (ATR, cm^−1^) 3345, 1769, 1696, 1638, 1590. MS (ESI^−^): *m/z* 382 [*M*‐H]^−^. ^1^H NMR (400 MHz, DMSO‐*d6*) *δ* 10.34 (s, 1H, NH–N), 9.20 (s, 1H, N=CH), 8.56 (s, 1H, NH cyc.), 7.90 (d, *J* = 8.2 Hz, 2H, arom.), 7.80 (d, *J* = 7.9 Hz, 2H, arom.), 7.73 (d, *J* = 7.6 Hz, 2H, arom.), 7.48 (t, *J* = 7.5 Hz, 2H, arom.), 7.44–7.30 (m, 2H, arom.), 7.04 (t, *J* = 7.8 Hz, 1H, arom.), 6.95 (d, *J* = 8.1 Hz, 1H, arom.), 6.87 (t, *J* = 7.8 Hz, 1H, arom.), 5.57 (s, 1H, C=CH). ^13^C NMR (101 MHz, DMSO‐*d6*) *δ* 169.57, 164.64, 156.39, 148.80, 143.18, 142.78, 139.59, 133.23, 129.52, 128.76, 128.56, 127.60, 127.24, 126.33, 126.09, 120.58, 120.19, 116.06. Elemental analysis calcd (%) for C_23_H_17_N_3_O_3_: C 72.05, H 4.47, and N 10.96; found: C 72.20, H 4.50, and N 10.85.

##### (Z)‐N′‐((E)‐naphthalen‐2‐ylmethylene)‐2‐(2‐oxo‐2H‐benzo[b][1,4]oxazin‐3(4H)‐ylidene)acetohydrazide **1j**

Orange solid. Yield: 43%. M.p. 242–244 °C. I.R. (ATR, cm^−1^) 3223, 1761, 1701, 1637, 1588. MS (ESI^−^): *m/z* 356 [*M*‐H]^−^. ^1^H NMR (400 MHz, DMSO‐*d6*) *δ* 10.33 (s, 1H, NH–N), 9.33 (s, 1H, N=CH), 8.58 (s, 1H, NH cyc.), 8.29 (s, 1H, arom.), 8.00 (s, 1H, arom.), 8.06–7.89 (m, 3H, arom.), 7.58 (tq, *J* = 7.7, 4.1, 2.4 Hz, 2H, arom.), 7.34 (d, *J* = 7.9 Hz, 1H, arom.), 7.03 (q, *J* = 7.2, 6.6 Hz, 1H, arom.), 6.95 (d, *J* = 7.9 Hz, 1H, arom.), 6.87 (t, *J* = 7.6 Hz, 1H, arom.), 5.57 (s, 1H, C=CH). ^13^C NMR (101 MHz, DMSO‐*d6*) *δ* 169.60, 164.66, 156.62, 148.83, 142.83, 134.75, 133.17, 131.86, 130.91, 129.12, 128.30, 128.18, 127.38, 126.33, 126.11, 122.75, 120.63, 120.20, 116.07. Elemental analysis calcd (%) for C_21_H_15_N_3_O_3_: C 70.58, H 4.23, and N 11.76; found: C 70.40, H 4.10, and N 11.65.

##### (Z)‐N′‐((E)‐benzofuran‐2‐ylmethylene)‐2‐(2‐oxo‐2H‐benzo[b][1,4]oxazin‐3(4H)‐ylidene)acetohydrazide **1k**

Orange solid. Yield: 66%. M.p. 221–224 °C. I.R. (ATR, cm^−1^) 3332, 1757, 1705, 1635, 1589. MS (ESI^−^): *m/z* 346 [*M*‐H]^−^. ^1^H NMR (400 MHz, DMSO‐*d6*) *δ*10.31 (s, 1H, NH–N), 9.29 (s, 1H, N=CH), 8.64 (s, 1H, NH cyc.), 7.74 (d, *J* = 7.9 Hz, 1H, arom.), 7.68 (d, *J* = 8.2 Hz, 1H, arom.), 7.56 (s, 1H, arom.), 7.44 (t, *J* = 7.8 Hz, 1H, arom.), 7.32 (d, *J* = 8.0 Hz, 2H, arom.), 7.04 (t, *J* = 7.7 Hz, 1H, arom.), 6.95 (d, *J* = 8.0 Hz, 1H, arom.), 6.86 (t, *J* = 7.6 Hz, 1H, arom.), 5.55 (s, 1H, C=CH). ^13^C NMR (101 MHz, DMSO‐*d6*) *δ* 169.55, 164.52, 155.45, 150.99, 148.97, 144.35, 143.03, 127.99, 127.41, 126.27, 126.20, 124.13, 122.77, 120.91, 120.17, 116.14, 113.77, 112.10, 87.59. Elemental analysis calcd (%) for C_19_H_13_N_3_O_4_: C 65.70, H 3.77, and N 12.10; found: C 65.70, H 3.65, and N 12.00.

##### (Z)‐N′‐((E)‐2‐nitrobenzylidene)‐2‐(2‐oxo‐2H‐benzo[b][1,4]oxazin‐3(4H)‐ylidene)acetohydrazide 1l

Light‐orange solid. Yield: 61%. M.p. 256–258 °C. I.R. (ATR, cm^−1^) 3362, 1759, 1709, 1633, 1594. MS (ESI^−^): *m/z* 351 [*M*‐H]^−^. ^1^H NMR (400 MHz, DMSO‐*d6*) *δ* 10.30 (s, 1H, NH–N), 9.71 (s, 1H, N=CH), 8.64 (s, 1H, NH cyc.), 8.10 (ddd, *J* = 18.6, 8.0, 1.4 Hz, 2H, arom.), 7.85 (td, *J* = 7.4, 1.2 Hz, 1H, arom.), 7.74 (ddd, *J* = 8.1, 7.4, 1.5 Hz, 1H, arom.), 7.32 (dd, *J* = 7.9, 1.5 Hz, 1H, arom.), 7.04 (ddd, *J* = 8.0, 7.3, 1.5 Hz, 1H, arom.), 6.94 (dd, *J* = 8.1, 1.4 Hz, 1H, arom.), 6.86 (td, *J* = 7.6, 1.4 Hz, 1H, arom.), 5.54 (s, 1H, C=CH). ^13^C NMR (101 MHz, DMSO‐*d6*) *δ* 169.35, 164.57, 150.37, 148.98, 143.02, 134.46, 132.12, 128.95, 128.57, 126.29, 126.18, 125.28, 120.94, 120.17, 116.13, 87.57. Elemental analysis calcd (%) for C_17_H_12_N_4_O_5_: C 57.96, H 3.43, and N 15.90; found: C 57.90, H 3.40, and N 16.15.

##### (Z)‐N′‐((E)‐2‐chlorobenzylidene)‐2‐(2‐oxo‐2H‐benzo[b][1,4]oxazin‐3(4H)‐ylidene)acetohydrazide **1m**

Yellow solid. Yield: 48%. M.p. 229–231 °C. I.R. (ATR, cm^−1^) 3330, 1766, 1705, 1642, 1593. MS (ESI^−^): *m/z* 340 [*M*‐H]^−^. ^1^H NMR (400 MHz, DMSO‐*d6*) *δ* 10.32 (s, 1H, NH–N), 9.64 (s, 1H, N=CH), 8.59 (s, 1H, NH cyc.), 8.03 (dd, *J* = 7.7, 1.8 Hz, 1H, arom.), 7.61–7.42 (m, 3 H, arom.), 7.32 (dd, *J* = 8.0, 1.5 Hz, 1H, arom.), 7.04 (td, *J* = 7.7, 1.5 Hz, 1H, arom.), 6.94 (dd, *J* = 8.1, 1.5 Hz, 1H, arom.), 6.86 (td, *J* = 7.6, 1.5 Hz, 1H, arom.), 5.57 (s, 1H, C=CH). ^13^C NMR (101 MHz, DMSO‐*d6*) *δ* 169.47, 164.63, 150.80, 148.86, 142.87, 134.53, 133.12, 131.58, 130.55, 128.26, 127.22, 126.24, 126.18, 120.70, 120.18, 116.07, 87.54. Elemental analysis calcd (%) for C_17_H_12_ClN_3_O_3_: C 59.75, H 3.54, and N 12.30; found: C 59.90, H 3.60, and N 12.30.

##### (Z)‐N′‐((E)‐2‐methylbenzylidene)‐2‐(2‐oxo‐2H‐benzo[b][1,4]oxazin‐3(4H)‐ylidene)acetohydrazide **1n**

Yellow solid. Yield: 58%. M.p. 217–219 °C. I.R. (ATR, cm^−1^) 3278, 1765, 1700, 1643, 1595. MS (ESI^−^): *m/z* 320 [*M*‐H]^−^. ^1^H NMR (400 MHz, DMSO‐*d6*) *δ* 10.33 (s, 1H, NH–N), 9.39 (s, 1H, N=CH), 8.54 (s, 1H, NH cyc.), 7.90–7.83 (m, 1H, arom.), 7.43 – 7.35 (m, 1H, arom.), 7.31 (q, *J* = 7.5 Hz, 3 H, arom.), 7.08–6.99 (m, 1H, arom.), 6.94 (dd, *J* = 8.1, 1.5 Hz, 1H, arom.), 6.86 (t, *J* = 7.3 Hz, 1H, arom.), 5.56 (s, 1H, C=CH), 2.45 (s, 3H, CH_3_). ^13^C NMR (101 MHz, DMSO‐*d6*) *δ* 169.59, 164.66, 155.92, 148.78, 142.73, 138.41, 132.17, 131.49, 126.78, 126.55, 126.34, 126.06, 120.53, 120.19, 116.02, 87.29, 19.41. Elemental analysis calcd (%) for C_18_H_15_N_3_O_3_: C 67.28, H 4.71, and N 13.08; found: C 67.40, H 4.80, and N 13.30.

##### Antimycobacterial and Cytotoxicity Studies: Cultures Preparation

*M. tuberculosis* H37Ra ATCC 25 177, H37Rv ATCC 27 294, INH‐R ATCC35822, Pyr‐R ATCC 35 828, Rifa‐R ATCC 35 838, and Strepto‐R ATCC 35 820 were used. The strains were cultured in Lowenstein–Jensen Medium at 37 °C in a 5% CO_2_ atmosphere. The cultures were then transferred to Middlebrook 7H9 broth, supplemented with 10% Albumin Dextrose Catalase (ADC) and 0.04% Tween 80, to prevent clumping. Cells were harvested, resuspended in saline, and adjusted to a (1) McFarland density. The suspension was diluted 1:20 in Middlebrook 7H9 broth, supplemented with 10% ADC and 0.04% Tween 80, and then sonicated to disrupt any remaining clumps. The absence of clumps was verified using Ziehl–Neelsen staining. Finally, the bacterial suspensions were diluted and seeded on Middlebrook 7H11 agar supplemented with 10% OADC, and the plates were incubated at 37 °C for 28*d* to determine the number of colonies.

##### Staging of the Solutions of the Test Compounds

The test substances were dissolved in DMSO at a concentration of 1–5 mg mL^−1^, depending on their solubility, and stored at −20 °C until needed. The substances were then diluted in Middlebrook 7H9 broth with ADC 10% to get final concentrations of the testing substances ranging from 64 to 0.03 μg mL^−1^.

##### Determination of Antimycobacterial Activity

The tested compounds were evaluated for their MICs against various *M. tuberculosis* strains, including resistant strains, using the resazurin microtiter assay with slight modifications.^[^ ^30^, ^31^ ^]^ Each solution (100 μL) of the tested compounds was added to 96‐well plates containing 100 μL of mycobacterial suspension, and the plates were incubated at 37 °C in a 5% CO_2_ atmosphere for 7 days. Resazurin solution (Sigma–Aldrich Co., St. Louis, MO, USA), 30 μL in 0.01% wt/vol in distilled water, was added to each well, incubated at 37 °C for 24–48 h, and a color change from blue to pink, indicating bacterial growth, was observed. MIC was determined as the lowest concentration of the test compound that prevented color change and inhibited bacterial growth. Isoniazid, rifampicin, and streptomycin (Sigma–Aldrich, St. Louis, MO, USA) were used as reference compounds for comparison. Each measurement was performed in triplicate under the conditions described.

##### Cytotoxicity Study

Vero 76 cells (Biobanking of the Veterinary Resources, BVR, IZSLER, Brescia, Italy) were grown in RPMI 1640 medium supplemented with 25 mM HEPES, 2 mM L‐glutamine, Penicillin 10 000 U mL^−1^, Streptomycin 10 000 μg mL^−1^, and 10% fetal bovine serum. All reagents were purchased from Gibco (Thermo Fisher Scientific, Waltham, MA, USA). Cells were seeded at 1 × 10^4^ cells mL^−1^, in 96‐well plastic microtiter plates (Euroclone) in RPMI 1640 containing no fetal bovine serum and antibiotics, and the test compounds at concentrations ranged between 3.12 and 100 μg mL^−1^, depending on their solubility in DMSO. To avoid interference from the solvent, the maximum concentration of DMSO was 2%. The cells were incubated at 37 °C in 5% CO_2_ and observed for morphological changes after 24 and 48 h of incubation. After 48 h, the effects on the proliferation of Vero cells were determined using a tetrazolium‐based colorimetric 3‐[4,5‐dimethylthiazol‐2‐yl]‐2,5‐diphenyltetrazolium bromide (MTT, Sigma–Aldrich) assay. The cytotoxic concentration (CC_50_) was measured using an automatic plate reader and defined as a 50% reduction in the absorbance value (*λ* = 570 nm) compared with the control untreated cells. Each measurement was performed in triplicate under the conditions described.

##### Computational Methods: MenB Binding Site Analysis

Pymol [version 2.6, Schrödinger LLC] was used for protein alignment, as well as for all the 3D molecular graphics. Binding site analysis was performed using two complementary methods. GRID software (version 2022.1)^[^ ^19^, ^20^, ^21^ ^]^ allowed the identification of significant hotspots in the binding pocket, its physicochemical characteristics, and key interactions using the OH2, DRY, N1, and O probes. Additional analysis of ligand–protein interactions and visualization of binding site residues were conducted using PoseView,^[^ ^16^, ^17^, ^18^, ^19^ ^]^ which generated 2D schematics. The software was accessed as a tool through the ProteinPlus webportal.^[^ ^32^, ^33^ ^]^ For both methods (GRID and PoseView), all options were used at the default.

##### Docking

Molecular docking simulations were performed using an HP server equipped with 64 processors and Linux OS (server beta1 @Dept. Pharm. Chem. Sciences, University of Trieste, Italy). Two sets of ligands were considered for docking: 1) literature‐derived ligands: molecules from Li et al.^[^ ^13^ ^]^ specifically compounds 1 (with an unsubstituted 1,4‐benzoxazine core), 12 (with the quinoxaline core), and 13 (with the benzothiazine core); and 2) novel derivatives: a series of 14 benzoxazin‐2‐one derivatives with a bicyclic core structure, an amide linker, and a terminal aromatic moiety. In particular, the carbonyl function of the hydrazone group enables an intramolecular hydrogen bond with the NH of benzoxazinone, conferring rigidity to the ligand; all ligands were modeled with restricted C—C rotation.

The ligands and the receptor were prepared using Python scripts from the AutoDockFR  interface for AutoDock,^[^ ^34^, ^35^ ^]^ whereas docking calculations were performed using Vina (1.2.5) docking software.^[^ ^36^, ^37^, ^38^ ^]^ The docking parameters were kept at default, except for exhaustiveness, which was set to 64 to enhance a larger sampling of ligand orientations to be evaluated. The grid box was built over 1‐hydroxy‐2‐naphthoyl‐CoA (ligand from 4QIJ), with the center of the grid set to the molecular barycenter and the size set according to the molecular dimensions over the three coordinates, by adding an extension of 5 Å on each side.

##### Molecular Dynamics

Crystal structure of MenB from *M. tuberculosis* in complex with 1‐HNA‐CoA (3 protein chains H J K, compounds 1a, 1g, 1m docked conformations and reference compound 1 from Li et al.^[^ ^13^ ^]^ 904 residues, 13 868 atoms with retained key waters 508, 524, 541, 575; (PDB ID: 4QIJ; resolution: 2.20 Å; by Guo et al.^[^ ^18^ ^]^) was prepared with Yasara (25.1.13) software.^[^ ^39^, ^40^ ^]^ Missing hydrogens were added, overlapping atoms were adjusted, missing residues and atoms were checked, hydrogen bonds were optimized, and residue ionization was assigned at pH 7.4.^[^ ^41^, ^42^ ^]^ A cubic box (9 Å away from all atoms) was solvated using the TIP3P water model and 0.9% NaCl (Na^+^ and Cl^−^ ions). After initial minimization with the steepest descent, annealing minimization was employed toward the local energy minimum. The simulation was initiated by assigning random initial velocities and slowly heating the system to 298 K. During the entire simulation, the energy of the system was monitored. Up to 250 ns MD simulations using the AMBER14 force field for the solute, GAFF and AM1BCC charges for ligands, and TIP3P for water were initiated. Experiments were performed in replicates with different seeds. The integration time step was 1.25 fs for bonded interactions and 2.5 fs for nonbonded interactions at a temperature of 298 K and a pressure of 1 atm, coupled with the time average temperature and density.^[^ ^43^ ^]^ Nonbonded long‐range interactions were calculated using the Particle Mesh Ewald algorithm.^[^ ^44^ ^]^ The Shake or LINCS (or SETTLE for water molecules) algorithms were not used in the simulation. MD snapshots in 100 ps intervals were collected. The energy parameters of the systems were stable throughout the production runs, as were the RMSD of the protein backbones (see Supporting Information).

## Conflict of Interests

The authors declare no conflict of interest.

## Supporting information

Supplementary Material
