# Supplementary material for: Synthesis, Antimycobacterial Activity, and Computational Insight of Novel 1,4‐Benzoxazin‐2‐one Derivatives as Promising Candidates against Multidrug‐Resistant Mycobacterium Tuberculosis
Source: ChemMedChem. 2025 Jun 10;20(14):e202500073. doi: 10.1002/cmdc.202500073 (PMC12276033; doi:10.1002/cmdc.202500073)
Supplement: Supplementary file 1 — Supplementary Material [file CMDC-20-e202500073-s001.pdf]

Supporting Information

Synthesis, Antimycobacterial Activity and Computational Insight of novel 1,4-Benzoxazin-2-one Derivatives as Promising Candidates Against Multidrug-Resistant *Mycobacterium tuberculosis*

Maria Grazia Mamolo,[a] Emanuele Carosati, [a] Diletta Pasin, [b] Alessandro De Logu, [c] Gianluigi Cabiddu, [c] Marko Jukič, [d,e] and Daniele Zampieri * [a]

[a] Department of Chemistry and Pharmaceutical Sciences, Via L. Giorgieri 1, University of Trieste, 34127 Trieste, Italy; dzampieri@units.it; emanuele.carosati@units.it; mamolo@units.it
[b] S.O.C. Experimental and Clinical Pharmacology, IRCSS, CRO Aviano, Via F. Gallini 2, 33081, Aviano, Italy; diletta.pasin@cro.it
[c] Department of Life and Environmental Sciences, University of Cagliari, Cittadella Universitaria di Monserrato, 09042, Monserrato (Cagliari), Italy; adelogu@unica.it; cabgianluigi@gmail.com
[d] Faculty of Chemistry and Chemical Engineering, University of Maribor, Smetanova ulica 17, SI-2000 Maribor (Slovenia)
[e] Faculty of Mathematics, Natural Sciences and Information Technologies, University of Primorska, Glagoljaška ulica 8, SI-6000 Koper (Slovenia)

Correspondence: dzampieri@units.it; Tel.: +39-040-5583677

Table of Contents

1. Experimental data

- Spectral data page 2

- Details for docking calculations and docking figures page 20

- MD graphs page 27

<u>1H-NMR and 13C-NMR of compounds **2**, **3** and **1a-n** in DMSO-d_6 as deuterated solvent</u>

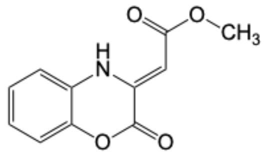

(Z)-Methyl-2-(2-oxo-2H-benzo[b][1,4]oxazin-3(4H)-ylidene)acetate **2**

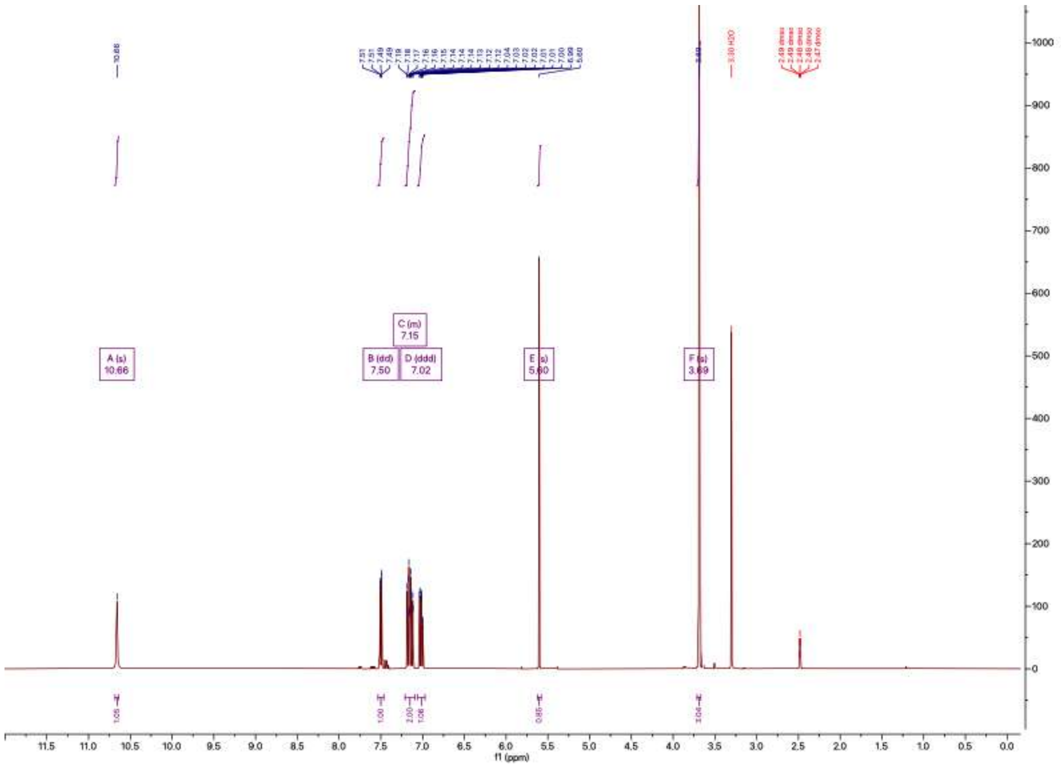

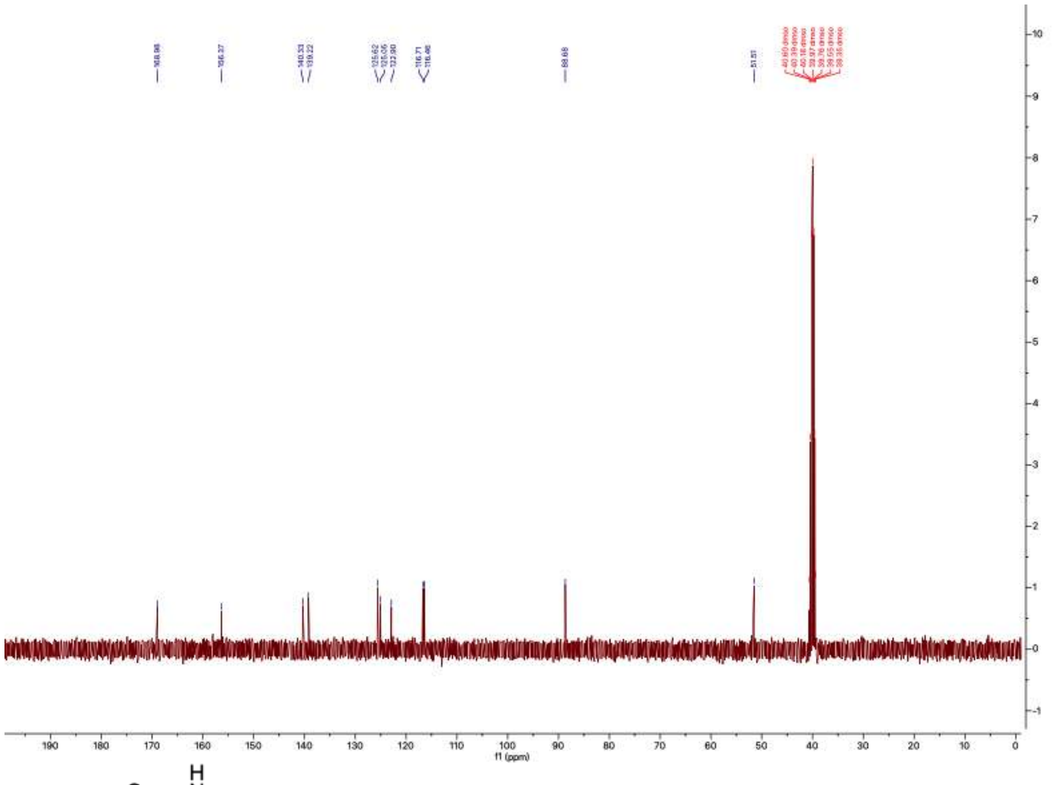

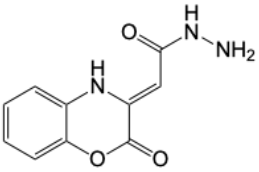

(Z)-2-(2-oxo-2H-benzo[b][1,4]oxazin-3(4H)-ylidene)acetohydrazide **3**

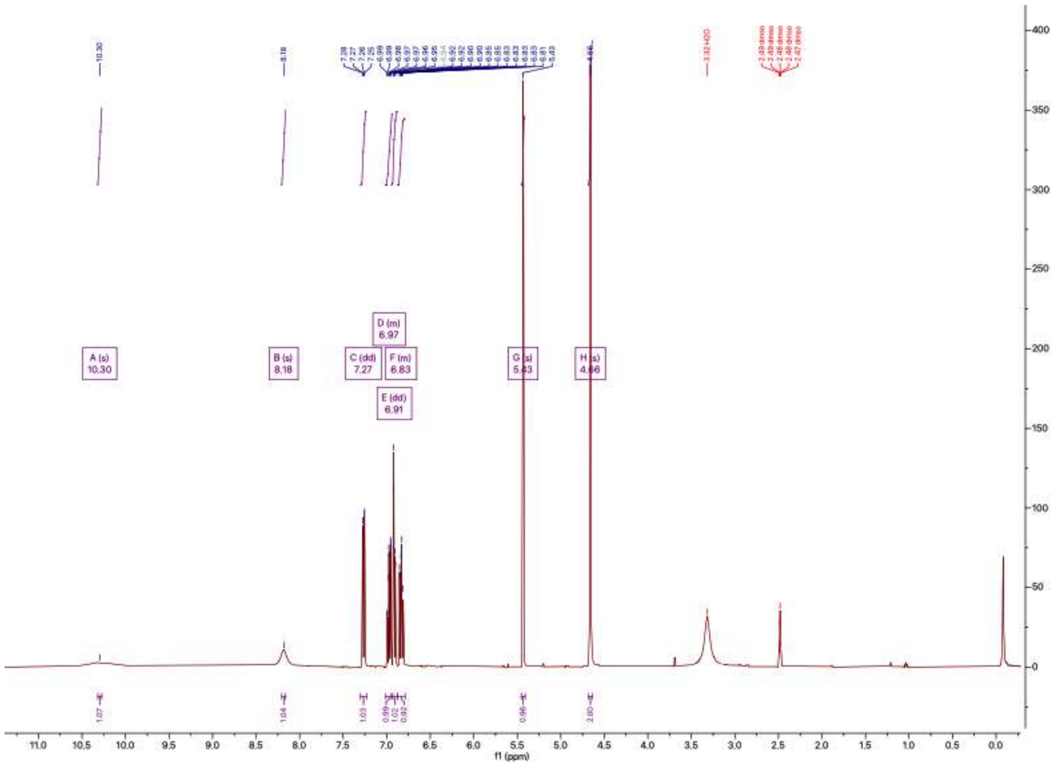

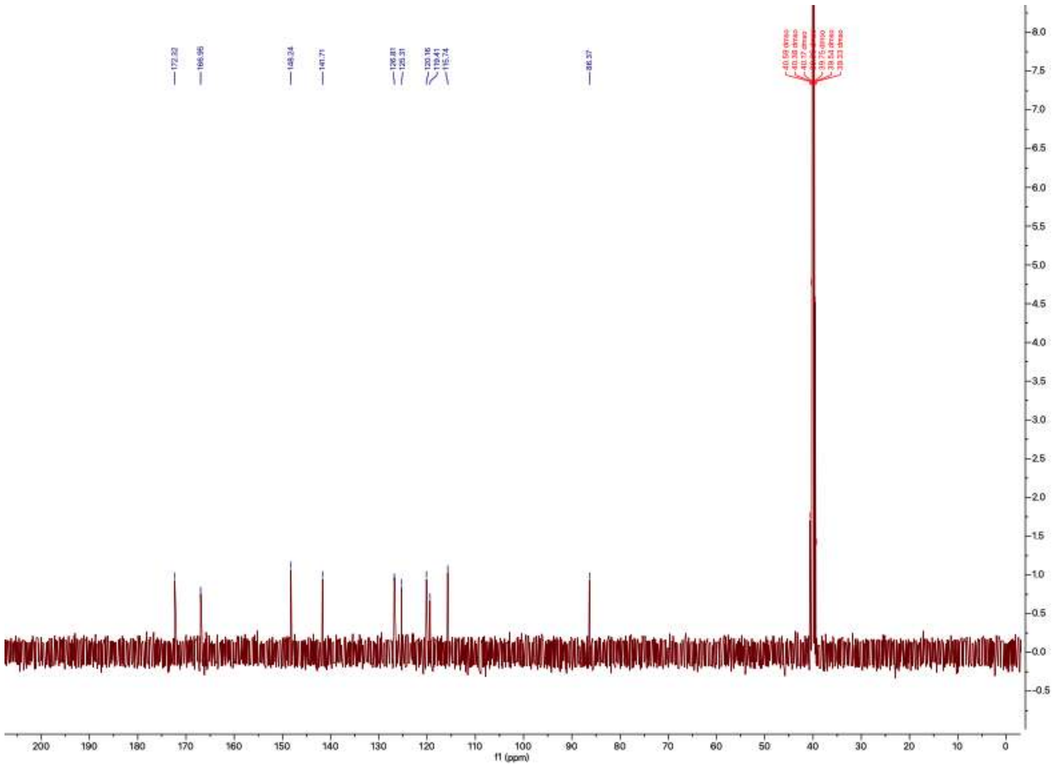

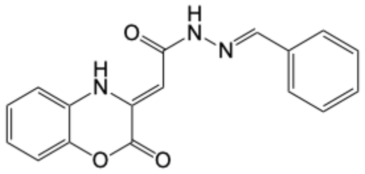

(Z)-N'-((E)-benzylidene)-2-(2-oxo-2H-benzo[b][1,4]oxazin-3(4H)-ylidene)acetohydrazide **1a**

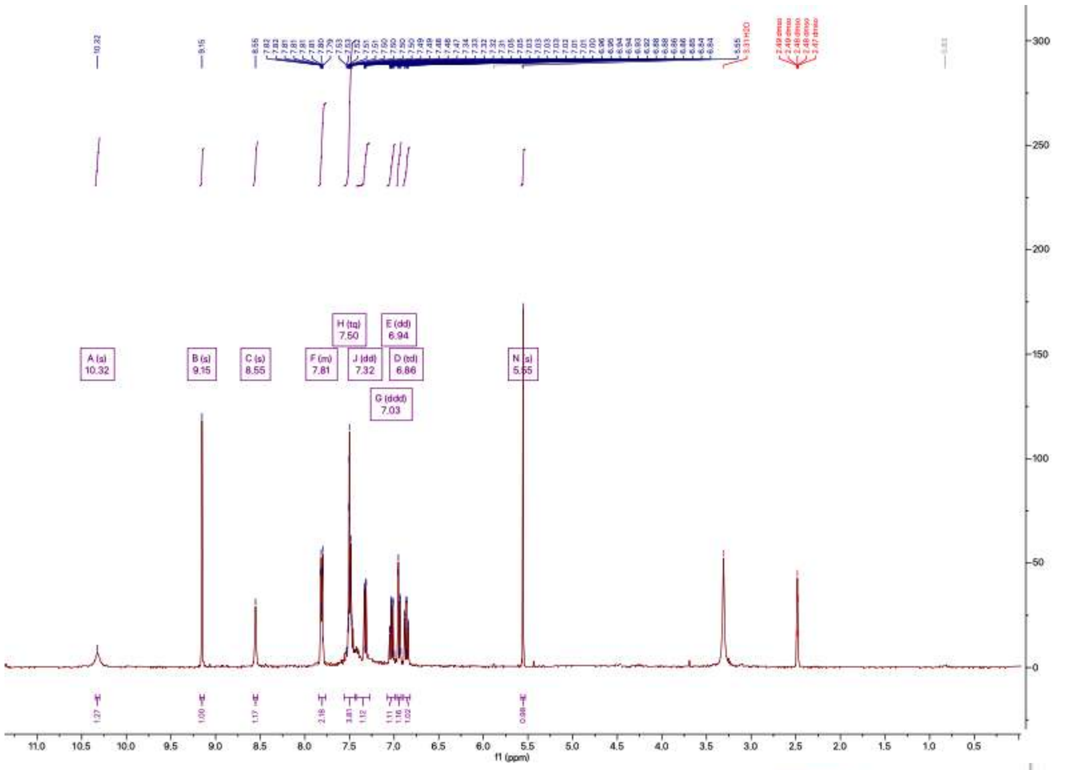

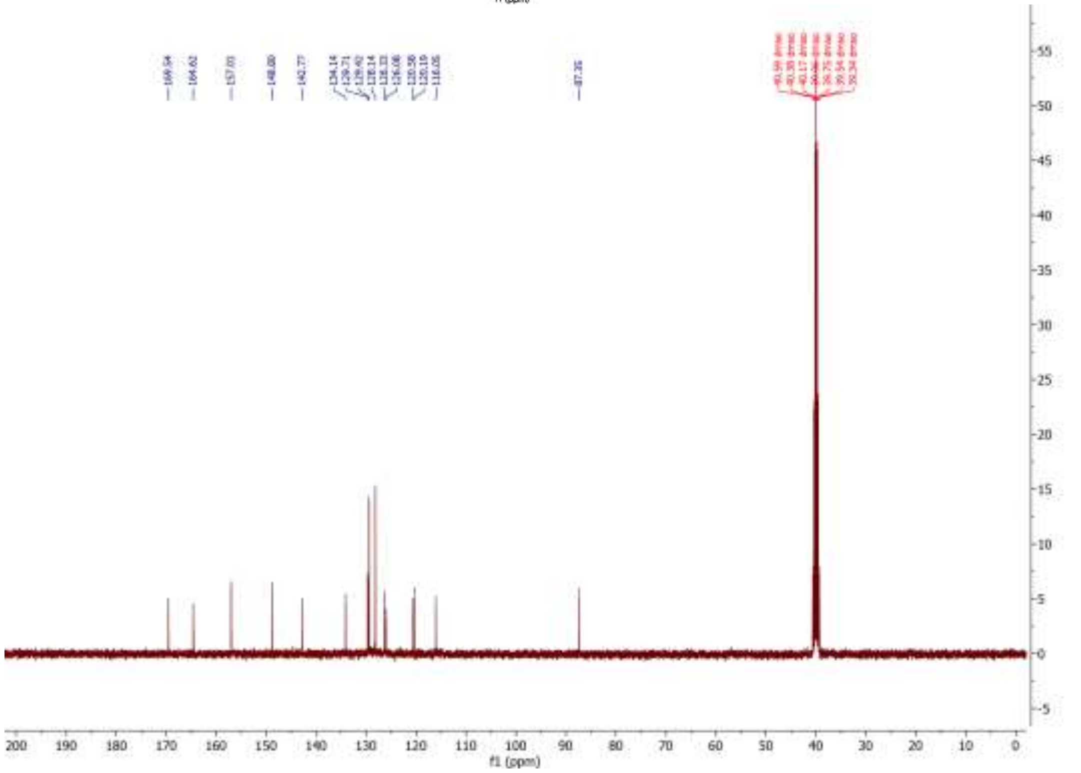

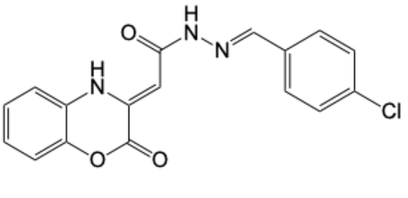

(Z)-N′-((E)-4-chlorobenzylidene)-2-(2-oxo-2H-benzo[b][1,4]oxazin-3(4H)-ylidene)acetohydrazide **1b**

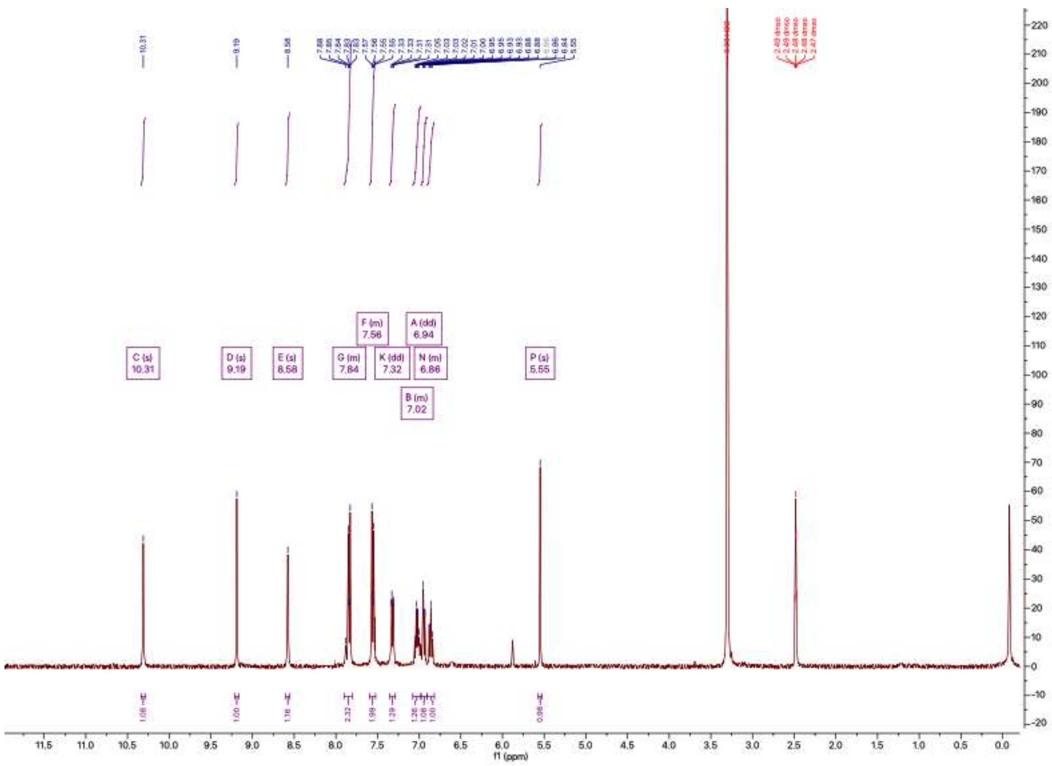

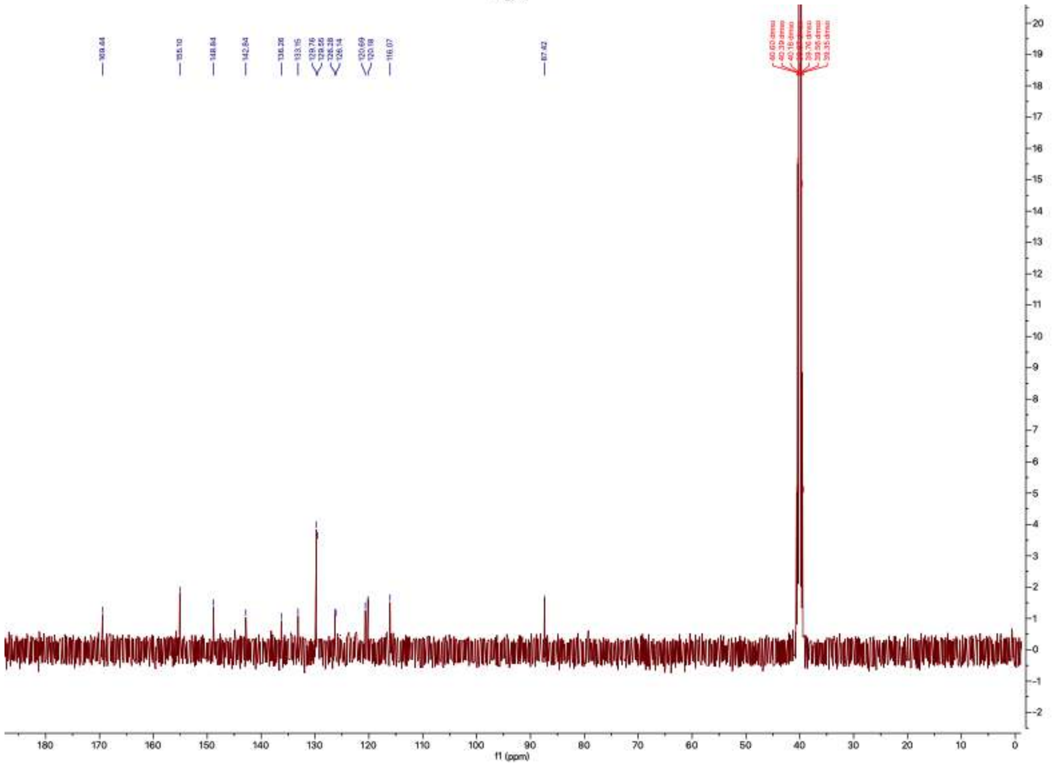

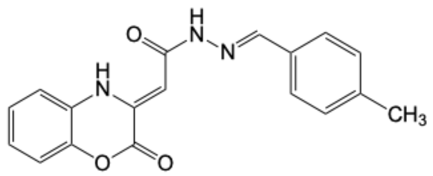

(Z)-N'-((E)-4-methylbenzylidene)-2-(2-oxo-2H-benzo[b][1,4]oxazin-3(4H)-ylidene)acetohydrazide **1c**

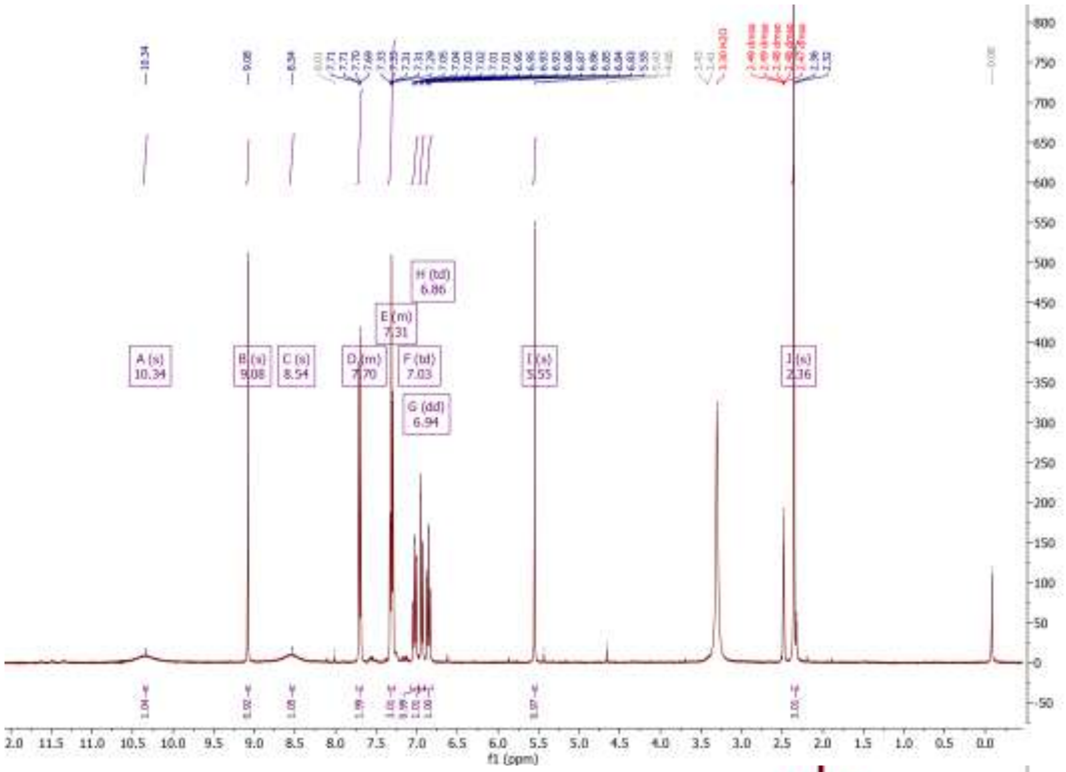

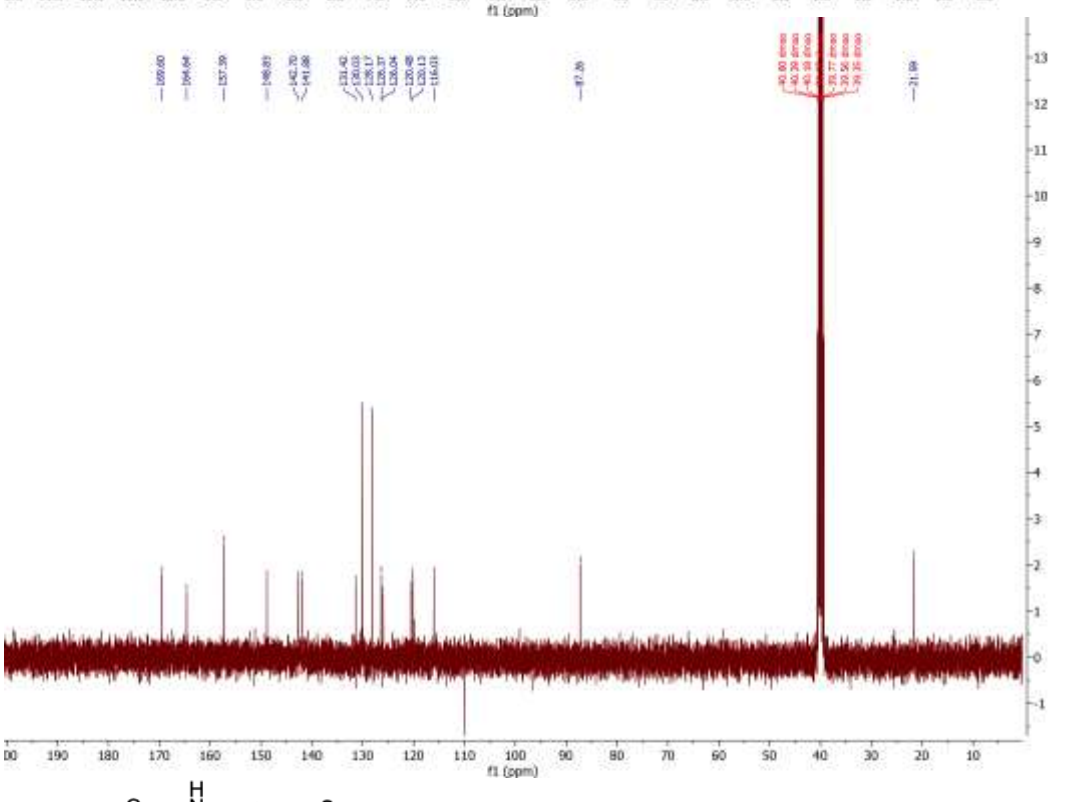

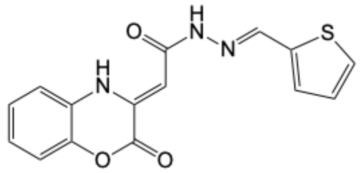

(Z)-2-(2-oxo-2H-benzo[b][1,4]oxazin-3(4H)-ylidene)-N′-((E)-thiophen-2-ylmethylene)acetohydrazide **1d**

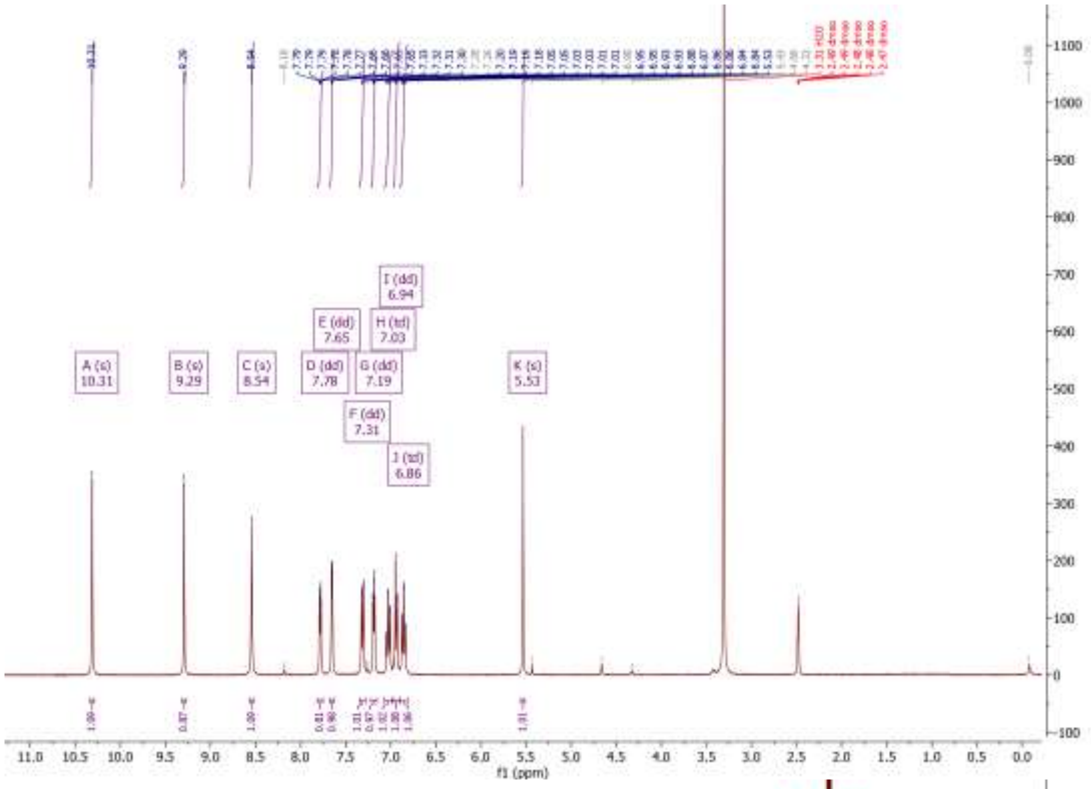

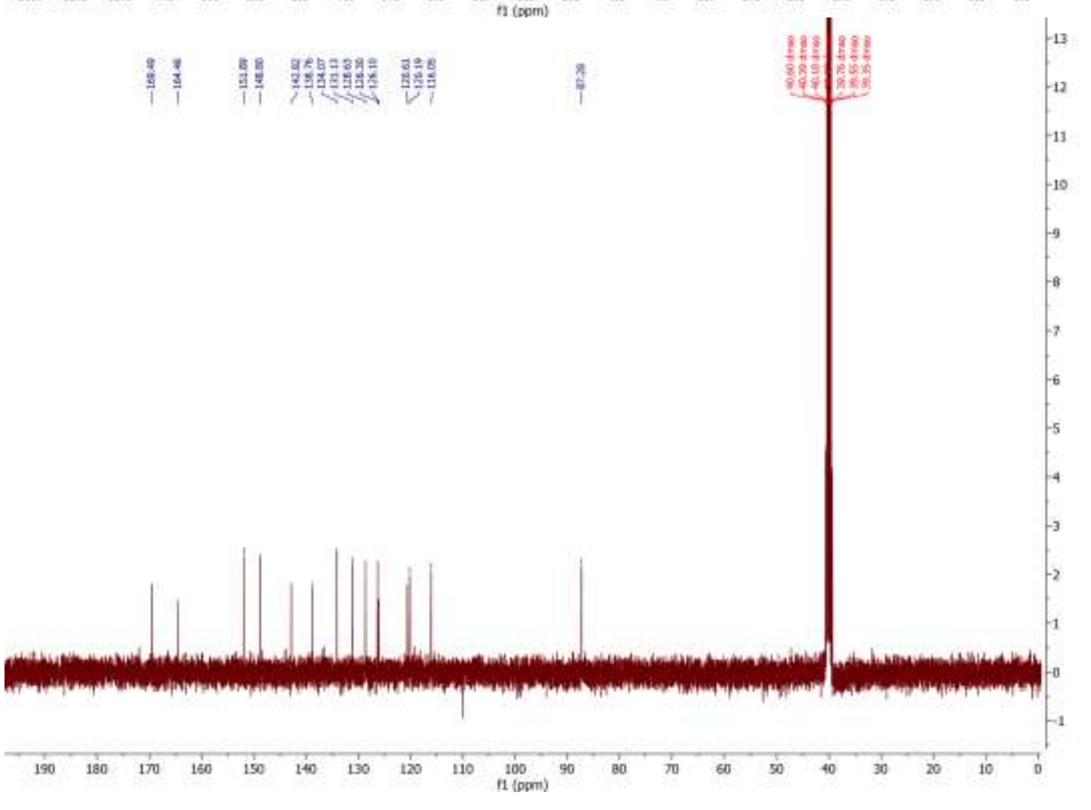

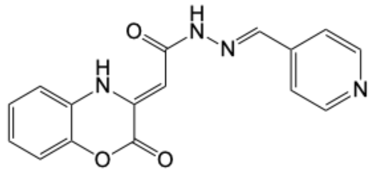

(Z)-2-(2-oxo-2H-benzo[b][1,4]oxazin-3(4H)-ylidene)-N′-((E)-pyridin-4-ylmethylene)acetohydrazide **1e**

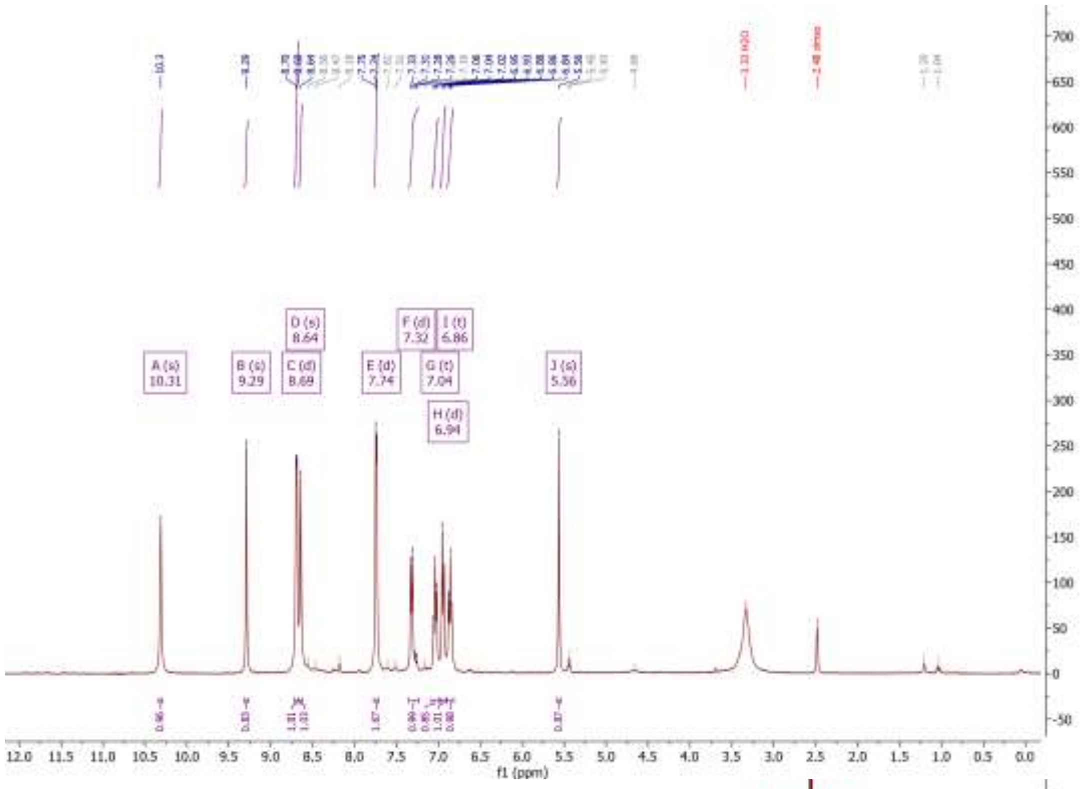

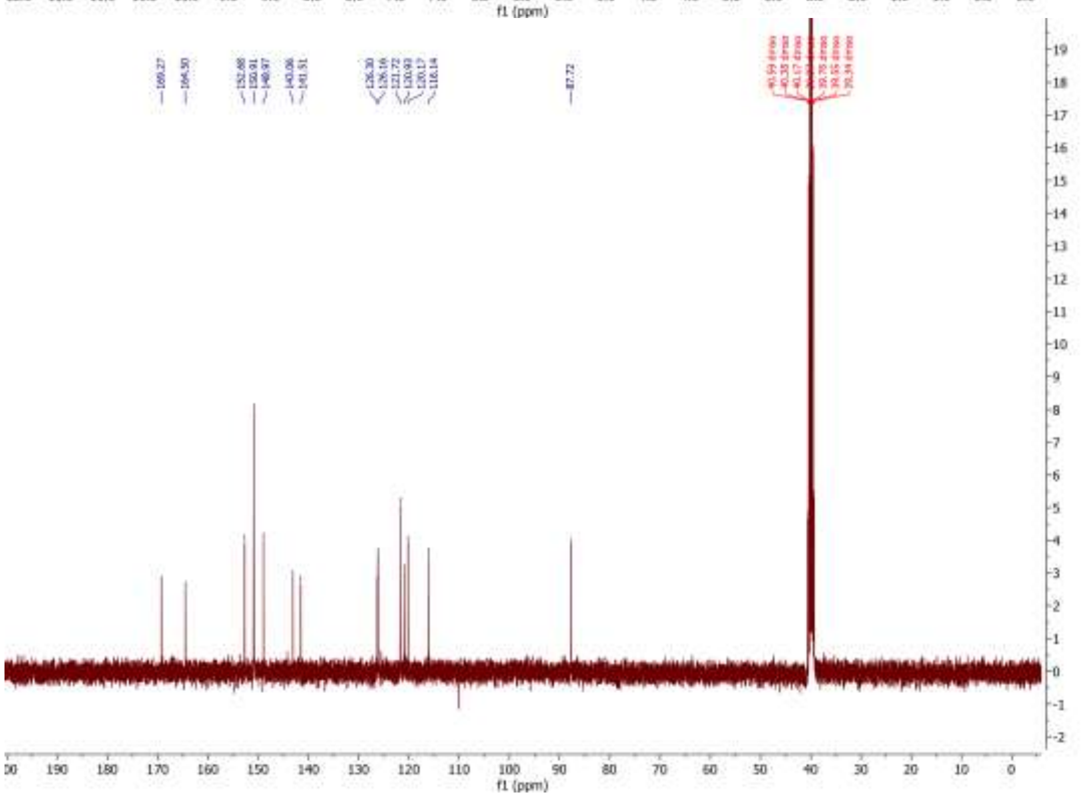

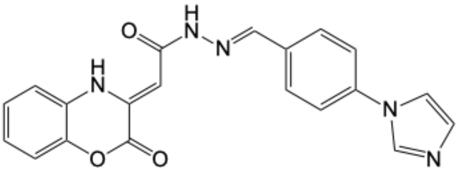

(Z)-N′-((E)-4-(1H-imidazol-1-yl)benzylidene)-2-(2-oxo-2H-benzo[b][1,4]oxazin-3(4H)-ylidene)acetohydrazide **1f**

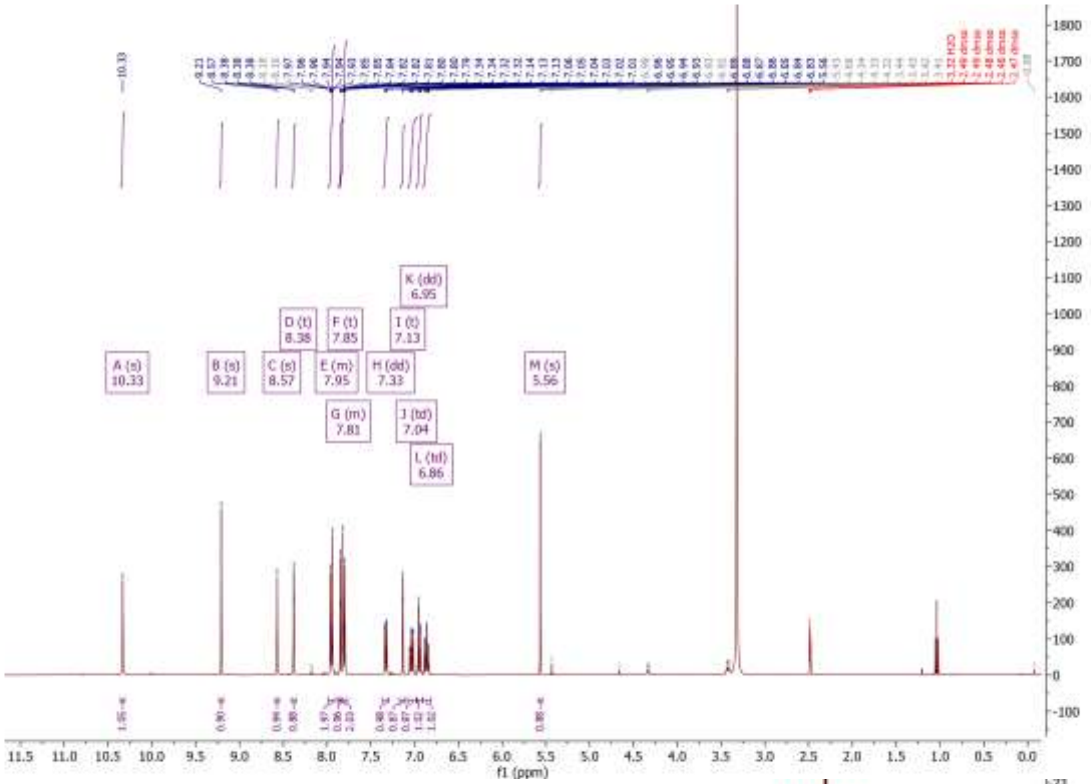

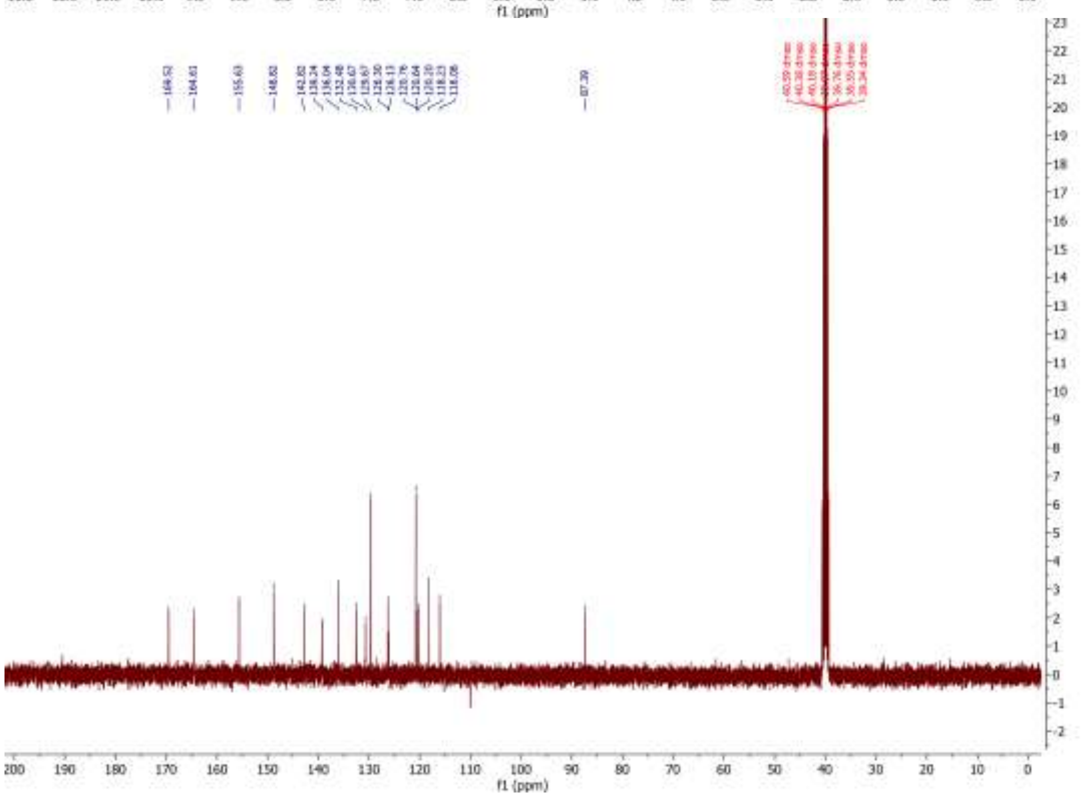

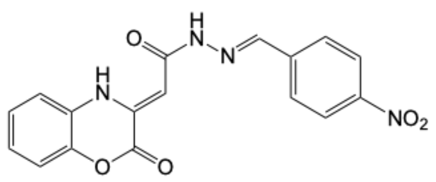

(Z)-N′-((E)-4-nitrobenzylidene)-2-(2-oxo-2H-benzo[b][1,4]oxazin-3(4H)-ylidene)acetohydrazide **1g**

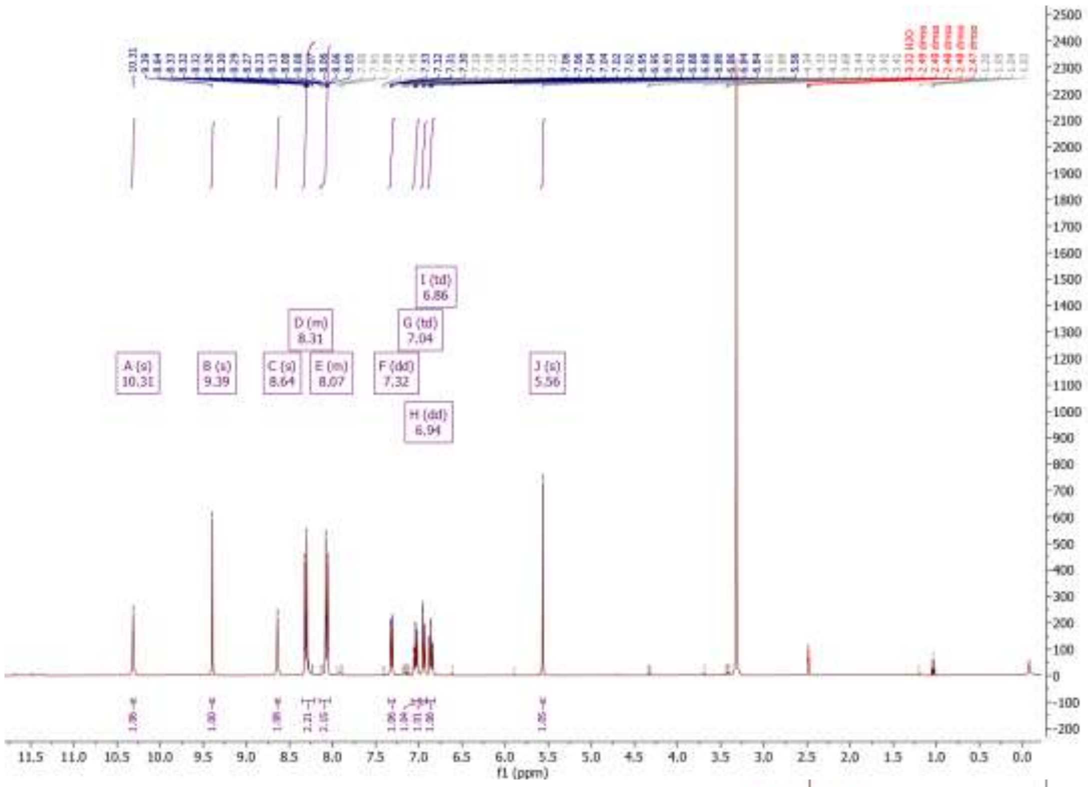

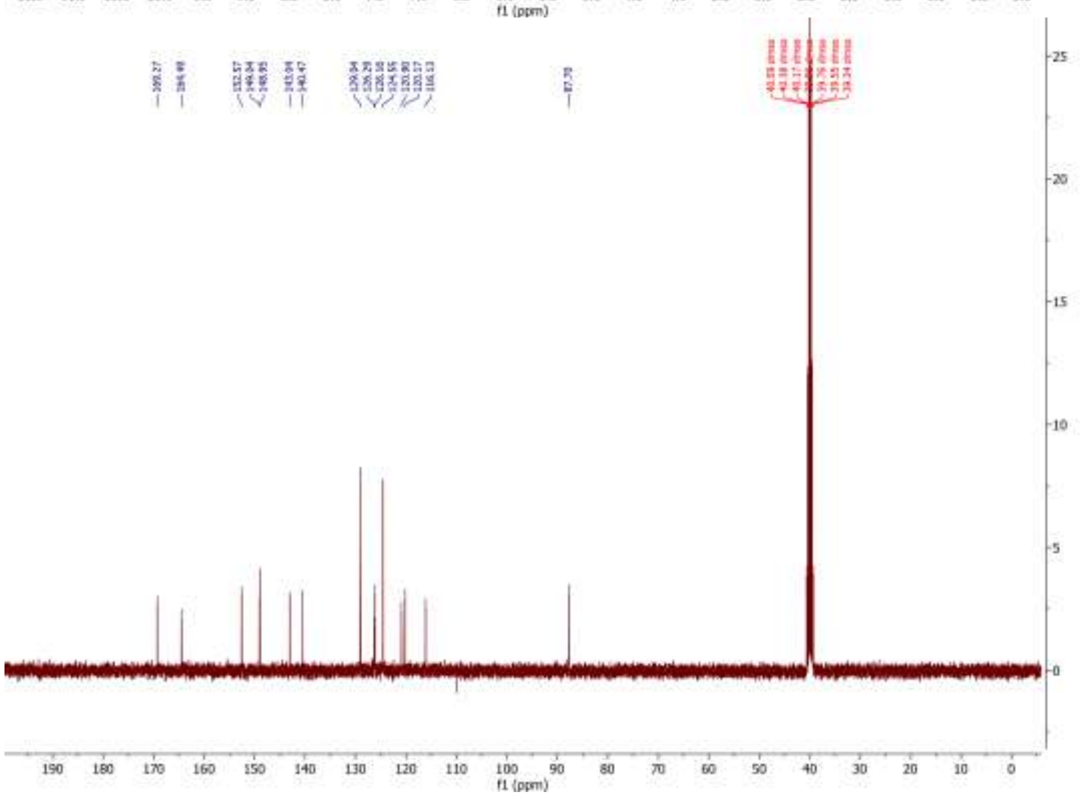

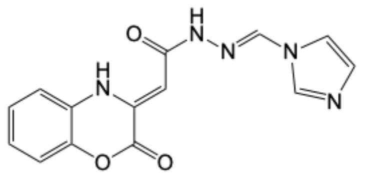

(Z)-N′-((E)-(1H-imidazol-2-yl)methylene)-2-(2-oxo-2H-benzo[b][1,4]oxazin-3(4H)-ylidene)acetohydrazide1H **1h**

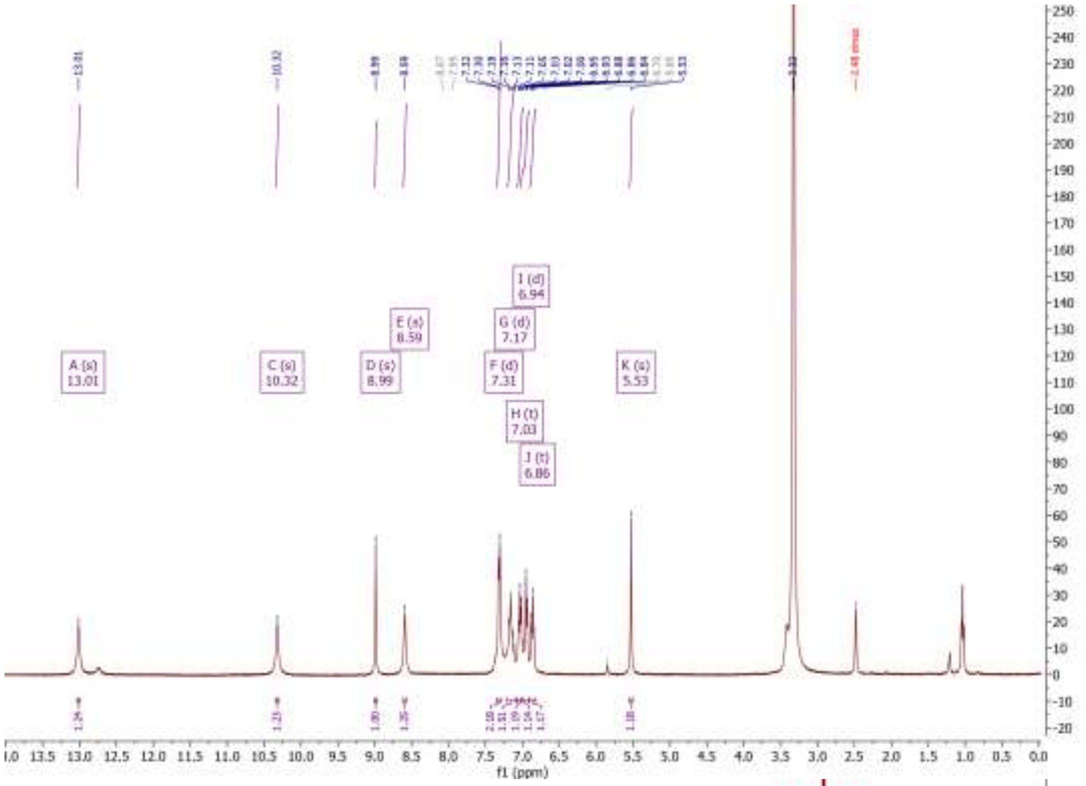

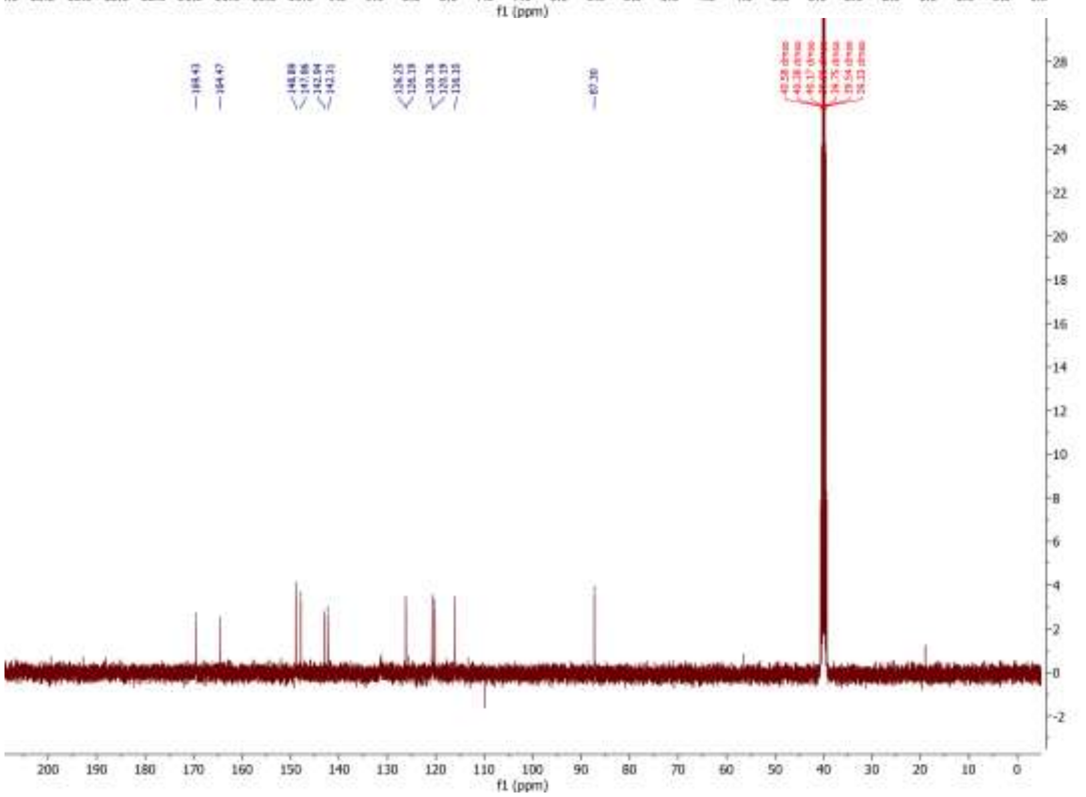

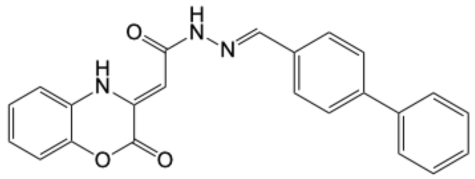

(Z)-N′-((E)-[1,1′-biphenyl]-4-ylmethylene)-2-(2-oxo-2H-benzo[b][1,4]oxazin-3(4H)-ylidene)acetohydrazide **1i**

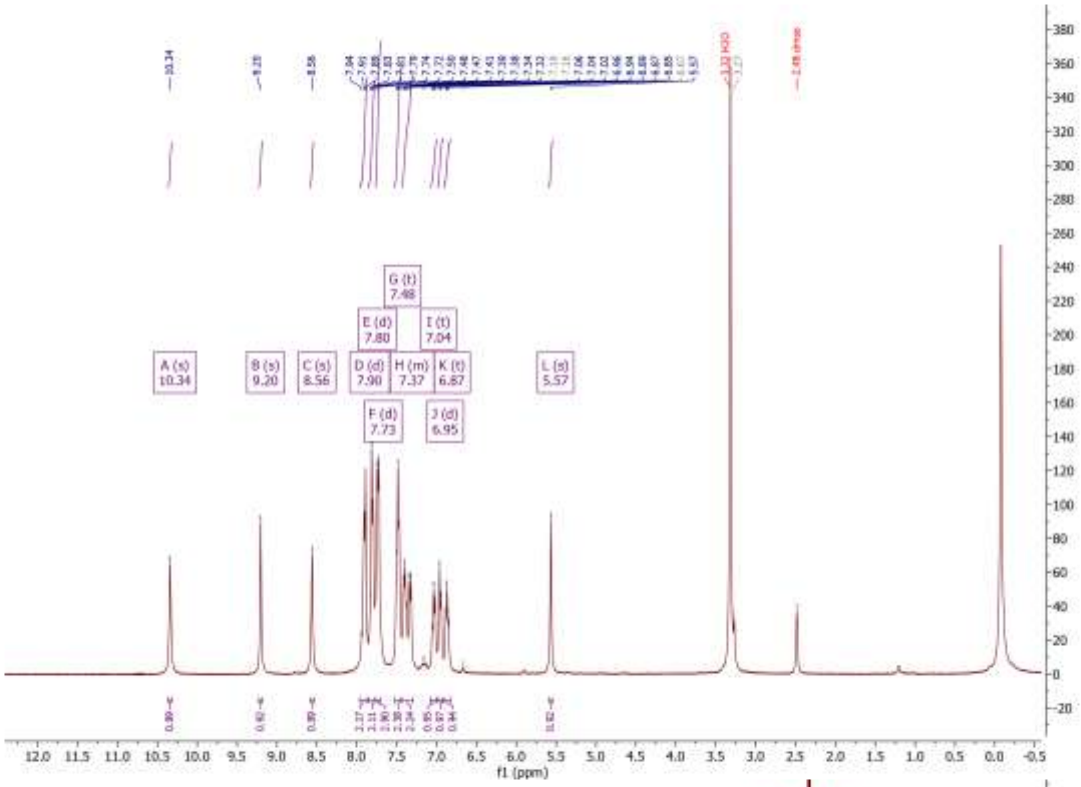

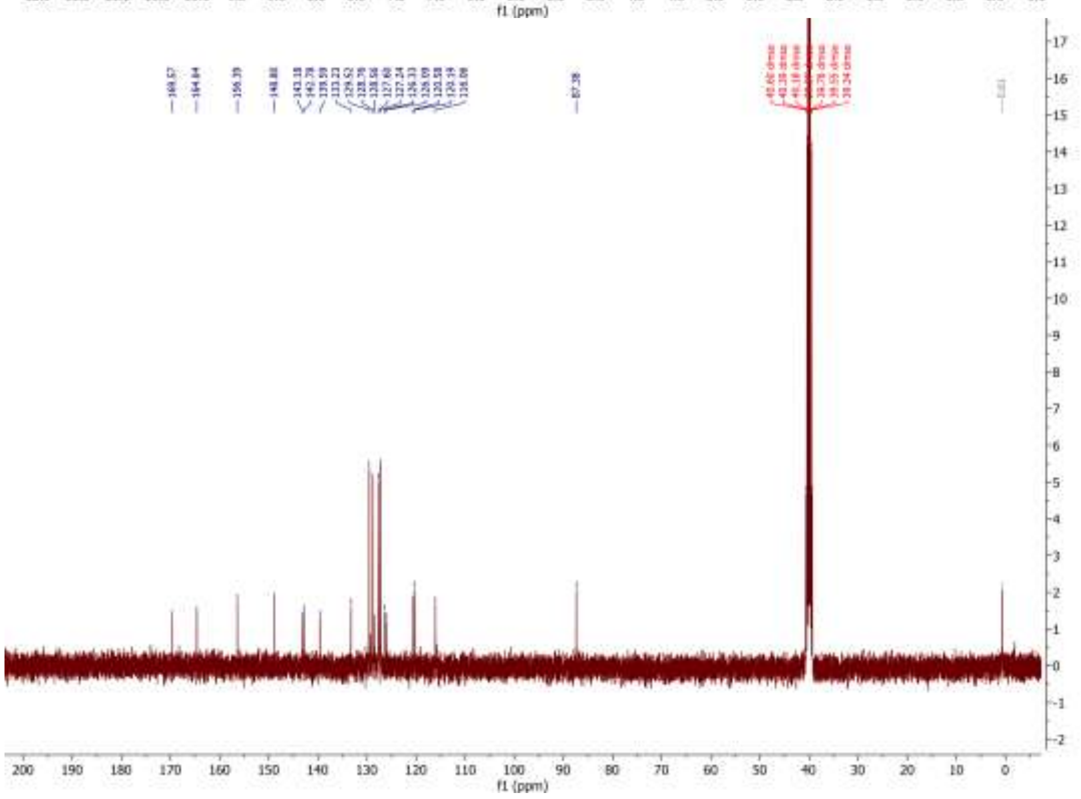

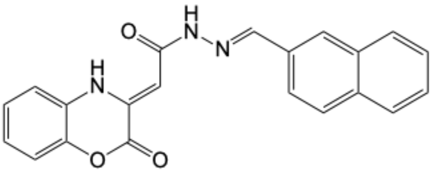

(Z)-N′-((E)-naphthalen-2-ylmethylene)-2-(2-oxo-2H-benzo[b][1,4]oxazin-3(4H)-ylidene)acetohydrazide **1j**

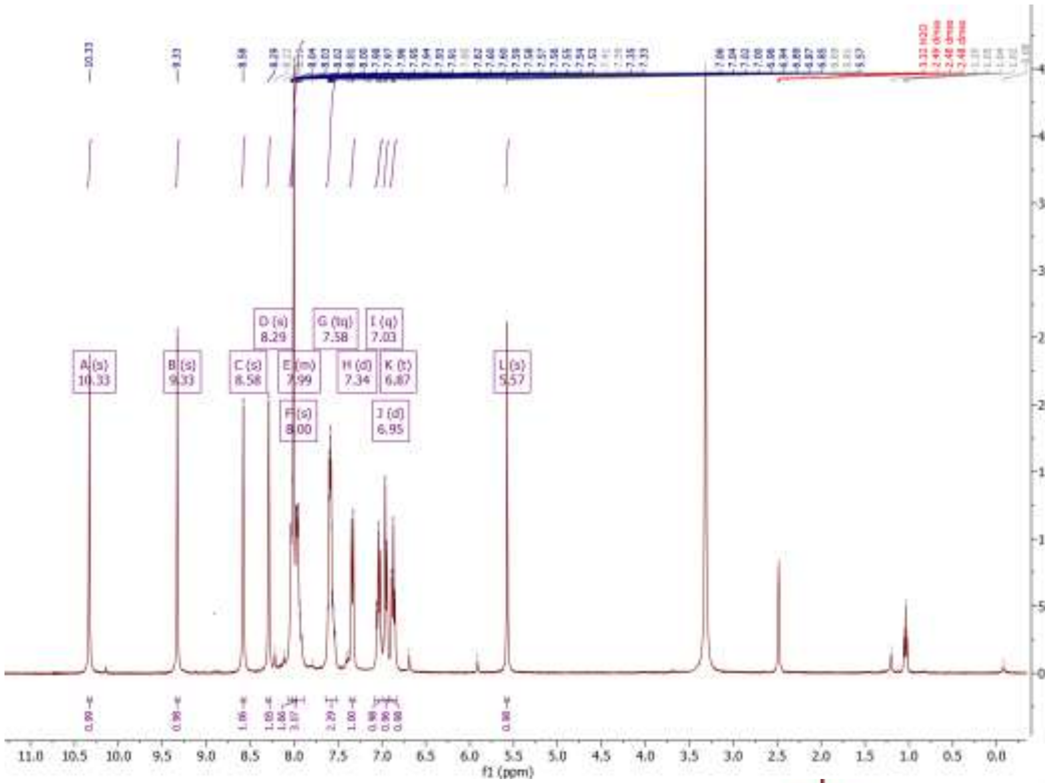

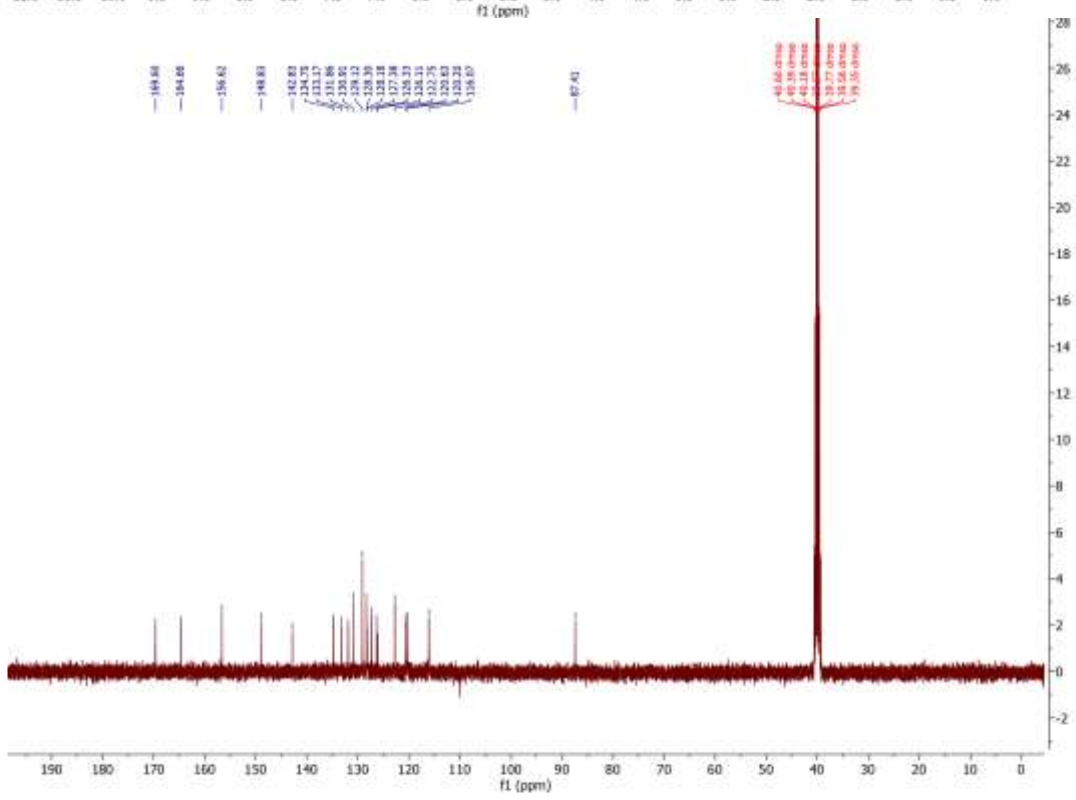

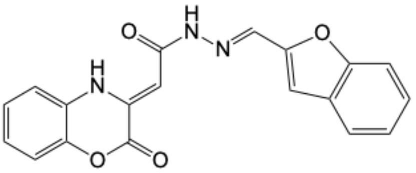

(Z)-N′-((E)-benzofuran-2-ylmethylene)-2-(2-oxo-2H-benzo[b][1,4]oxazin-3(4H)-ylidene)acetohydrazide **1k**

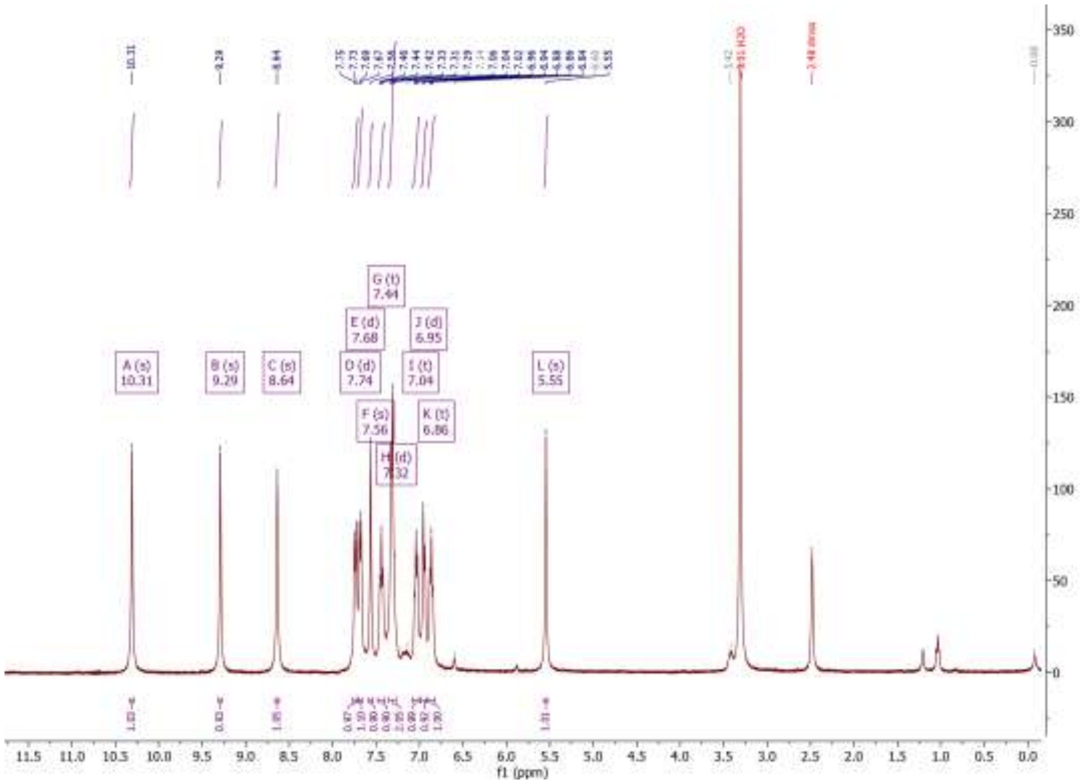

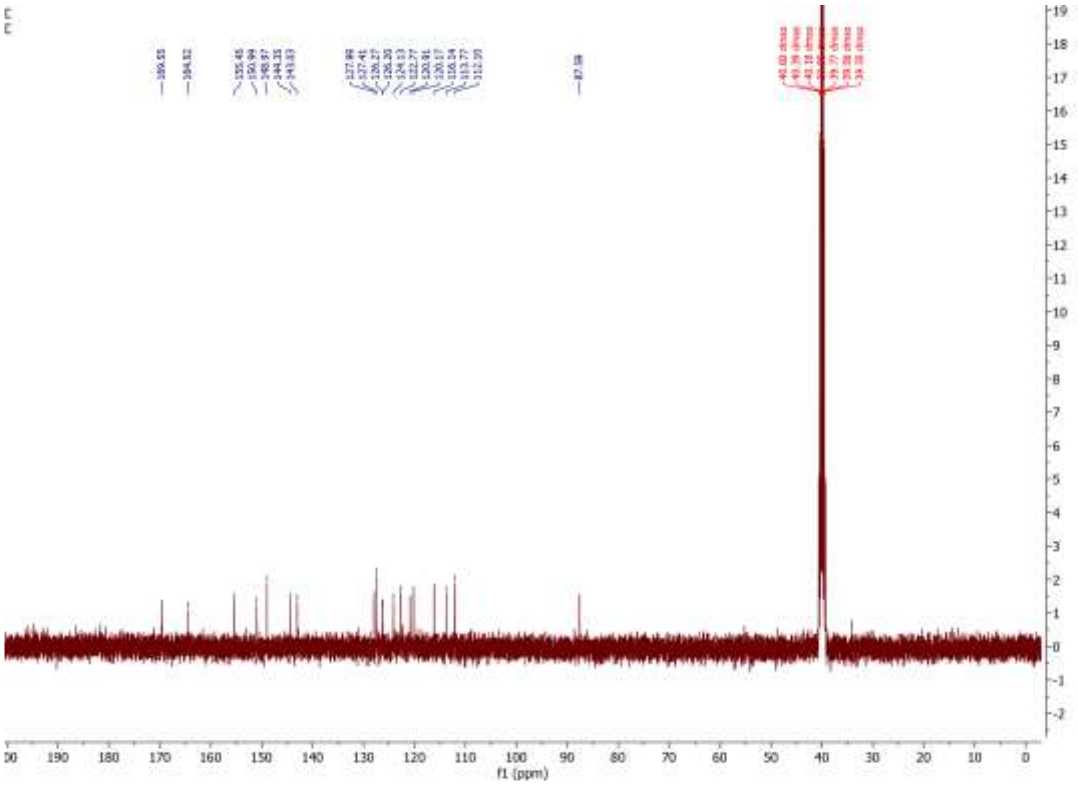

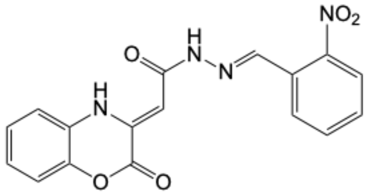

(Z)-N′-((E)-2-nitrobenzylidene)-2-(2-oxo-2H-benzo[b][1,4]oxazin-3(4H)-ylidene)acetohydrazide **1l**

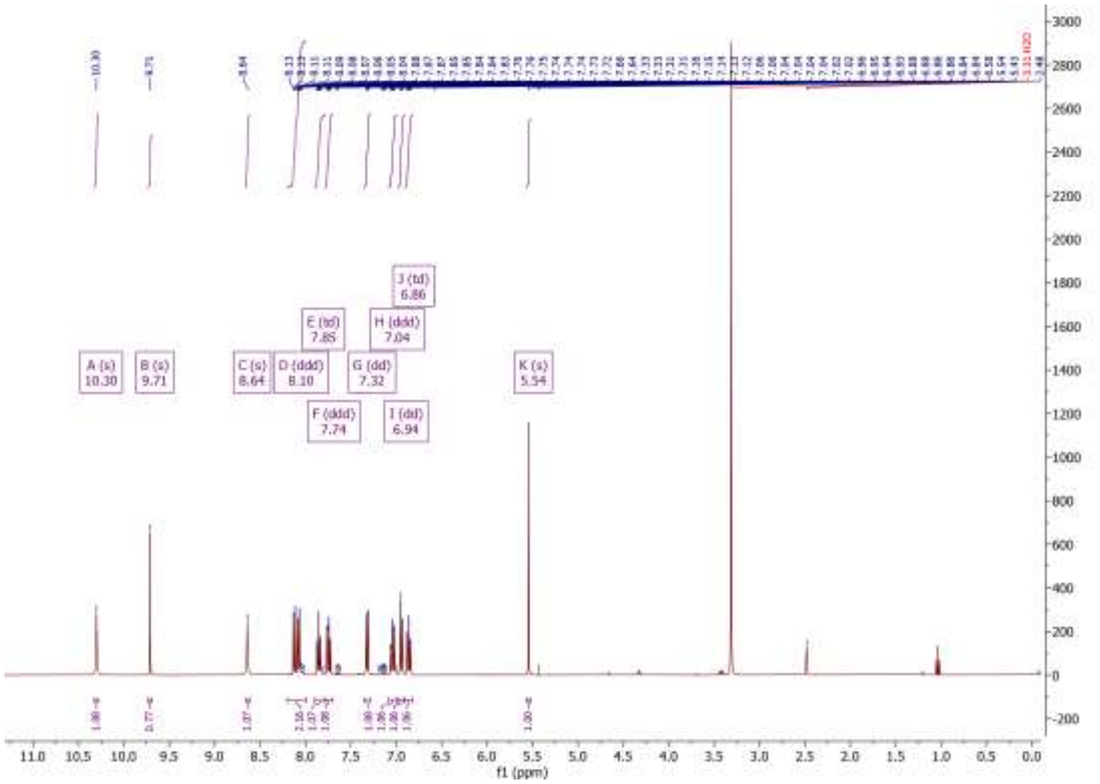

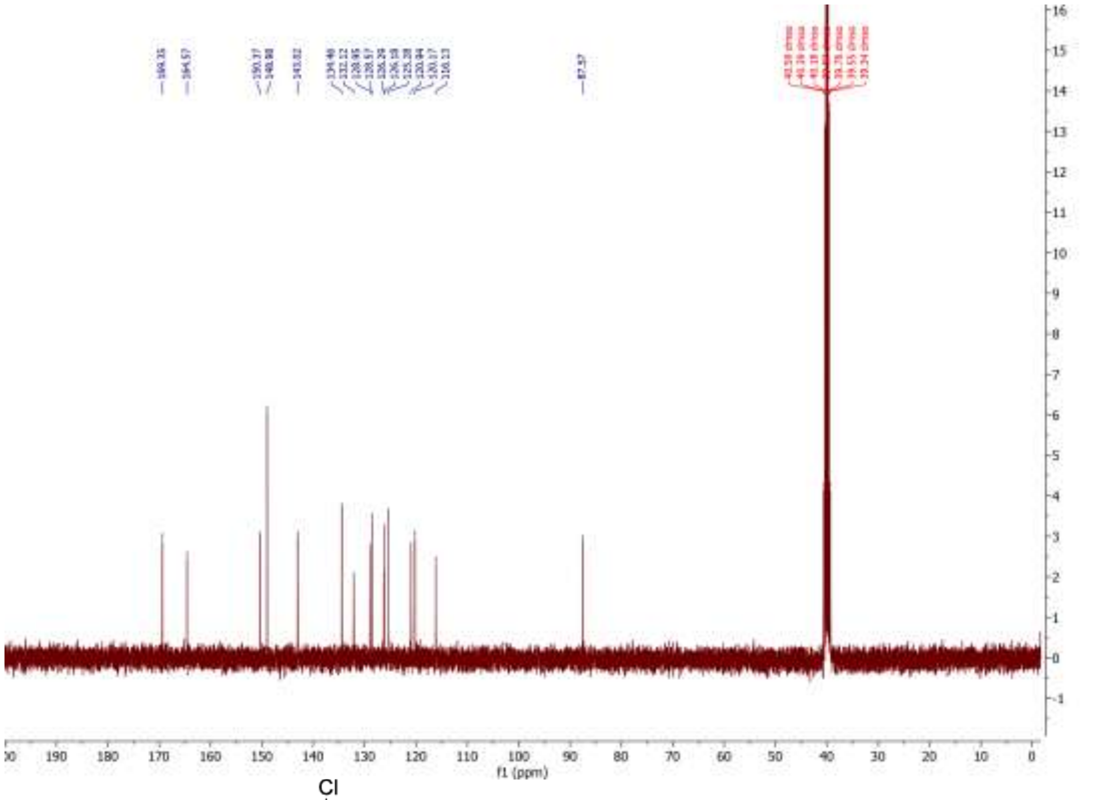

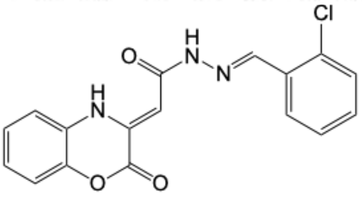

(Z)-N′-((E)-2-chlorobenzylidene)-2-(2-oxo-2H-benzo[b][1,4]oxazin-3(4H)-ylidene)acetohydrazide **1m**

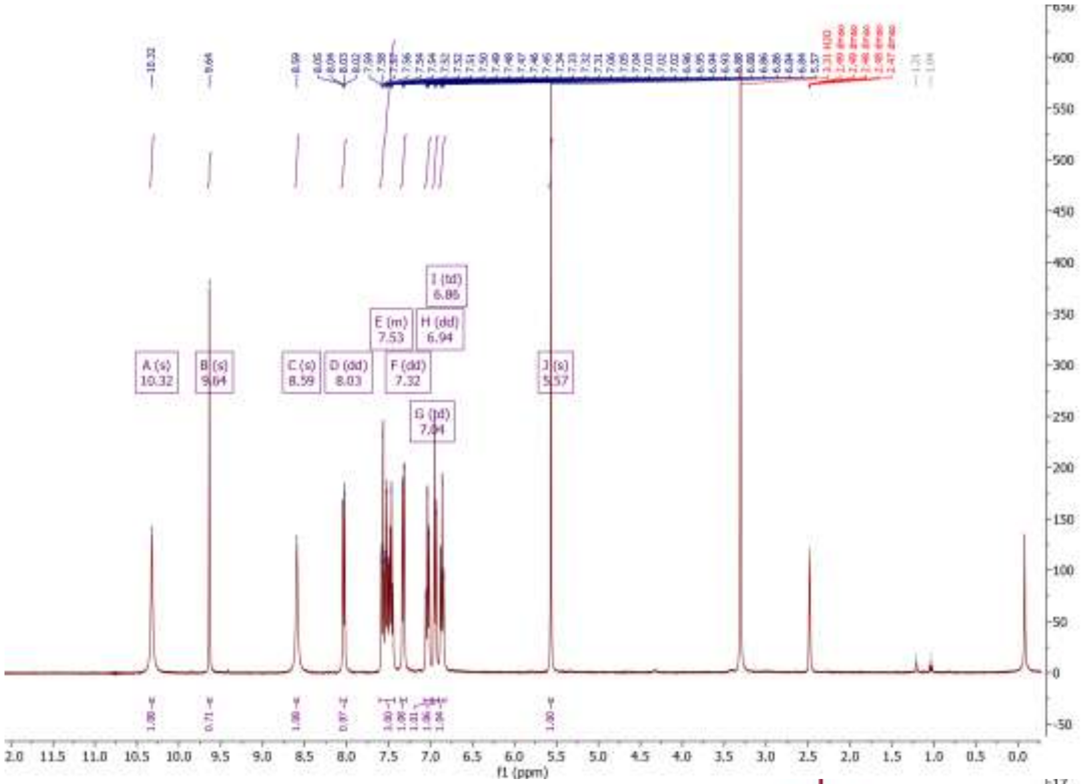

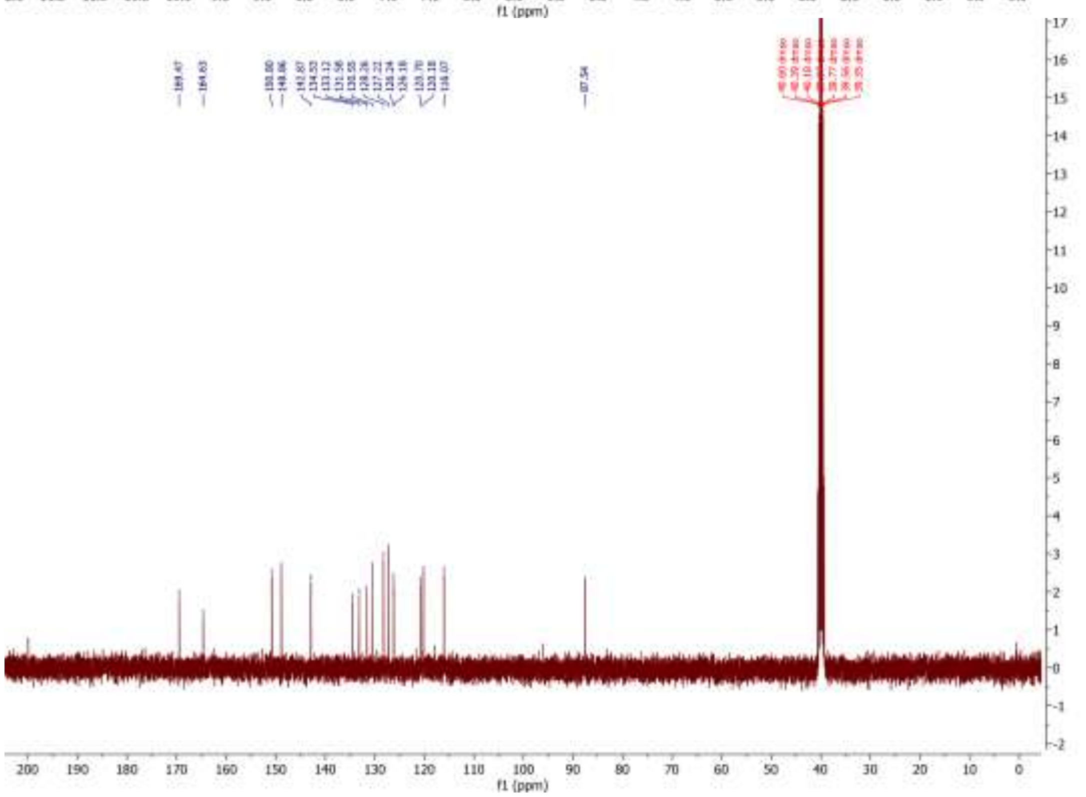

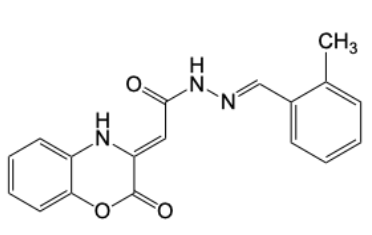

(Z)-N′-((E)-2-methylbenzylidene)-2-(2-oxo-2H-benzo[b][1,4]oxazin-3(4H)-ylidene)acetohydrazide **1n**

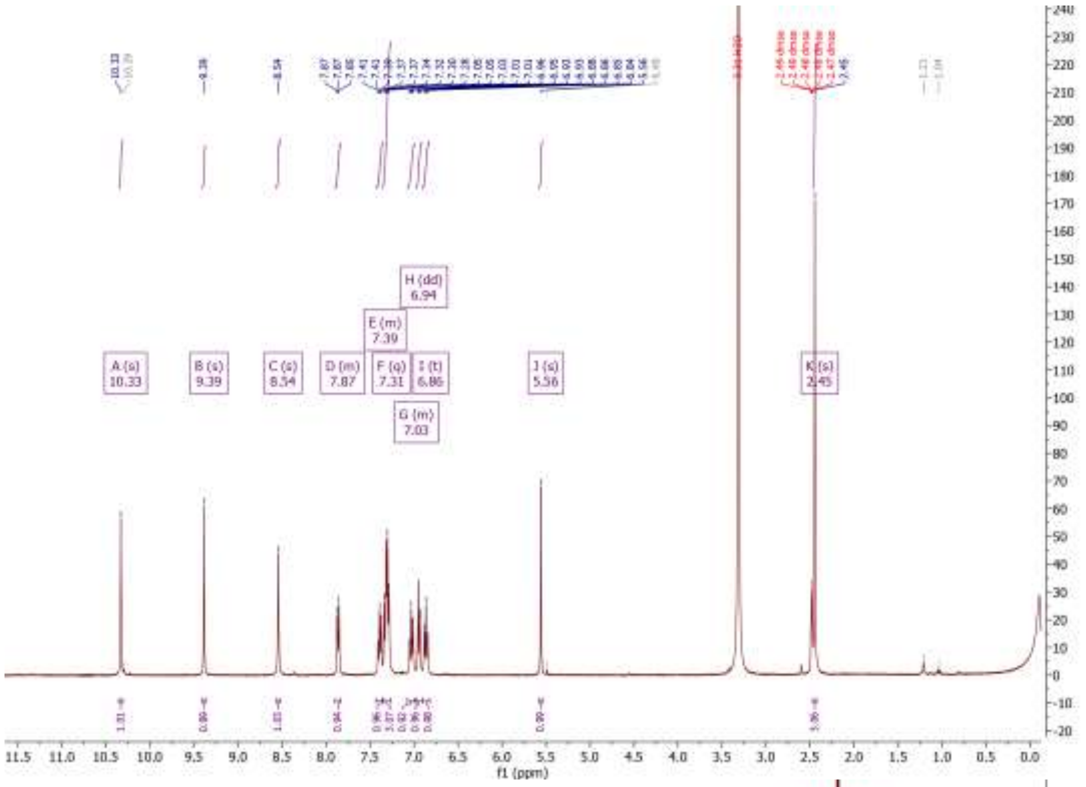

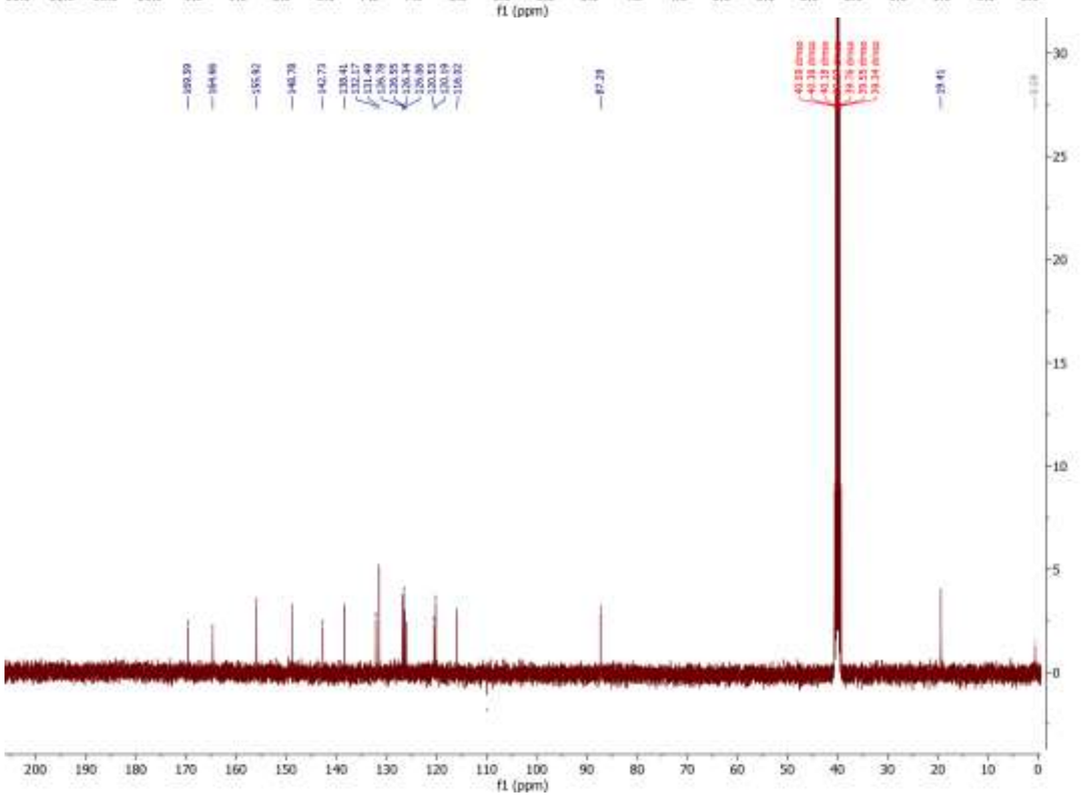

Details for docking calculations

1. **Preparation of library of ligands**

Used script mk_prepare_ligand.py on .mol2 files.
(mk_prepare_ligand.py -i $file.mol2 -o $file.pdbqt)

2. **Preparation of receptor**

Used script prepare_receptor
prepare_receptor -r 4qij_aligned_TAR_waters.pdb -A hydrogens -U nphs_lps_nonstdres -o 4qij_4waters.pdbqt

3. **PyMol visual inspection**

The target shown in the figures, simplified for visual inspection, includes: the residues in the range A14-D269 from the chain J and D270-F314 from the chain H; the ligand 1-HNA-CoA from chain J; four water molecules selected based on energy values of GRID water probe (we kept water molecules with strong interactions, below the threshold set to -10.0 kcal/mol) and calculated buriedness (we kept water molecules with no proximity to the surface of the protein). Finally, water molecules are shown as spheres (commands: "remove solvent"; "remove not ((chain J and resi 14-269) or (chain H and resi 270-314))"; "set sphere_scale, 0.3").
Lateral chains of residues are shown as lines when close (distance below 5Å) to the ligand 1-HNA-CoA, and one letter code is reported in the Calpha of each residue (commands: "select ligand_check, organic"; "select close_residues, (byres (all within 5 of organic)); "show lines, close_residues"; "label close_residues and name CA, oneletter + resi"; "set label_size, 12").

Results of docking calculations

PDB entry 4QIJ, with 1-HNA-CoA co-crystallysed

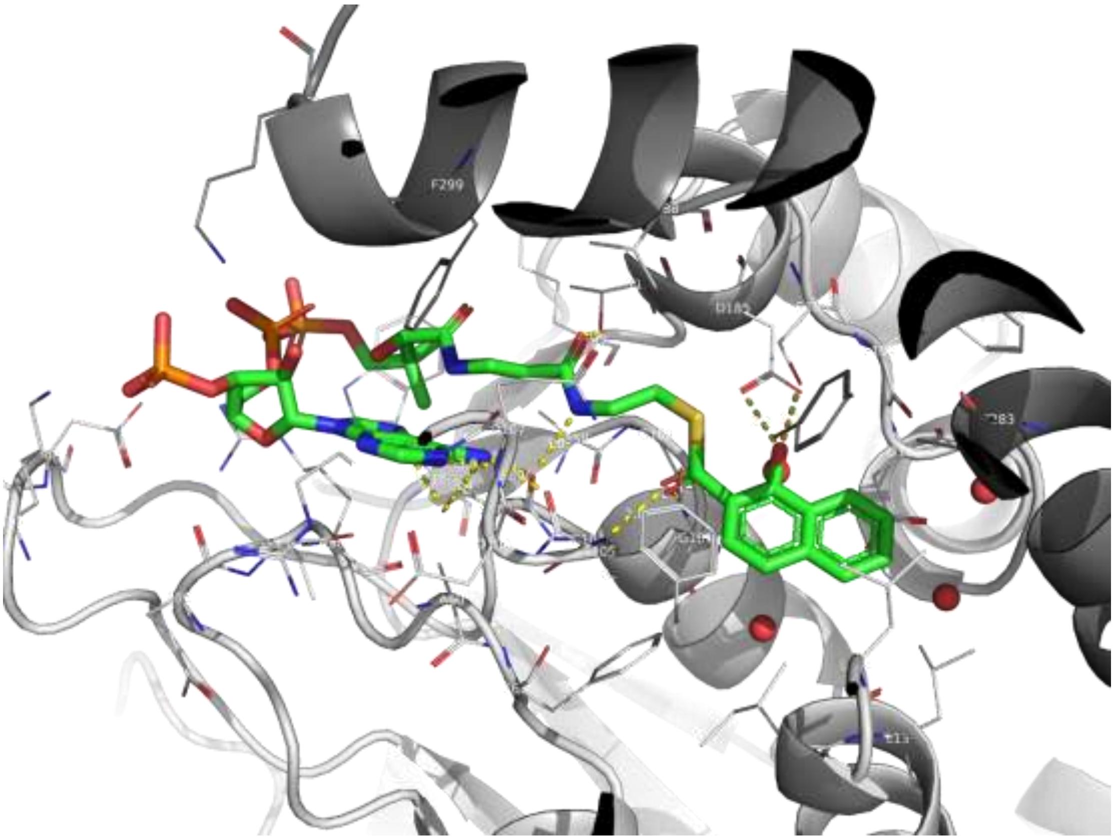

Figure S1. The ligand 1-HNA-CoA is green, whereas polar contacts are represented by dashed yellow lines

Docking results (first pose) for three derivatives from the paper from Li et al. [ref. 13 main text]

Compounds are colour-coded as follows: 1 (yellow), 12 (pink) and 13 (cyan).

**S1**<br>Compound **1** from Li *et al.*	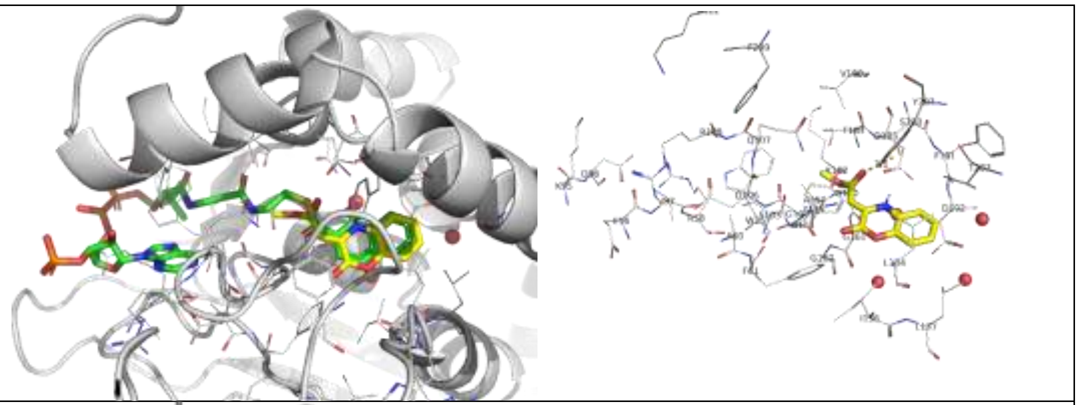
**S2**<br>Compound **12** from Li *et al.*	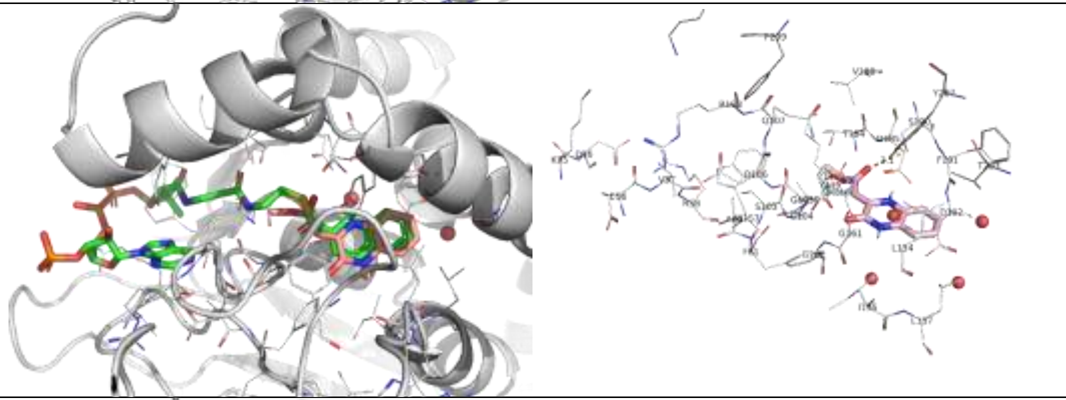
**S3**<br>Compound **13** from Li *et al.*	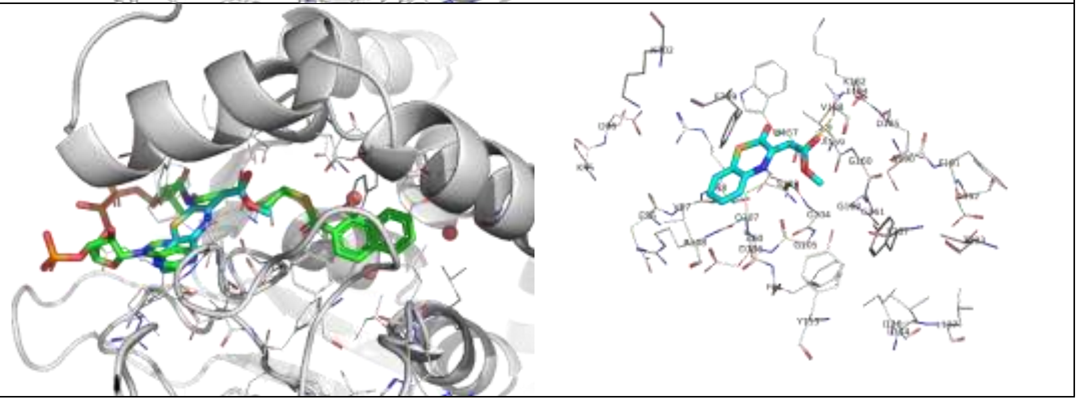

Docking results (first pose) for the 14 synthesised derivatives (1a-n).

**S4**<br>Compound **1a**	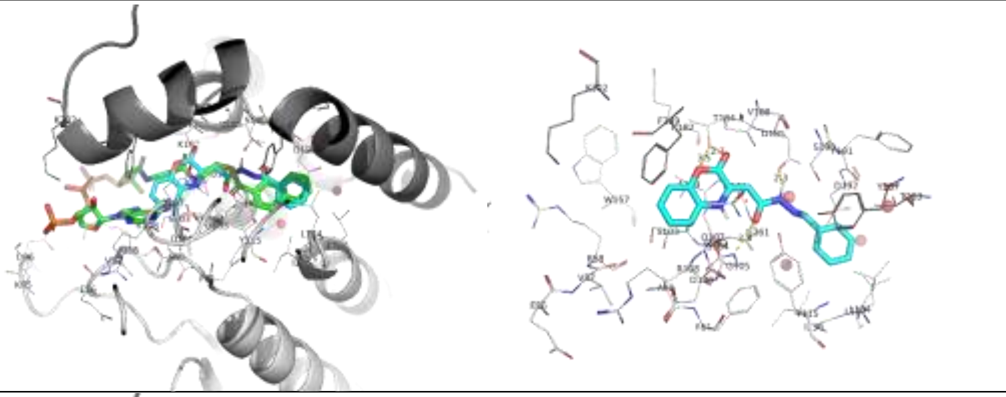
**S5**<br>Compound **1b**	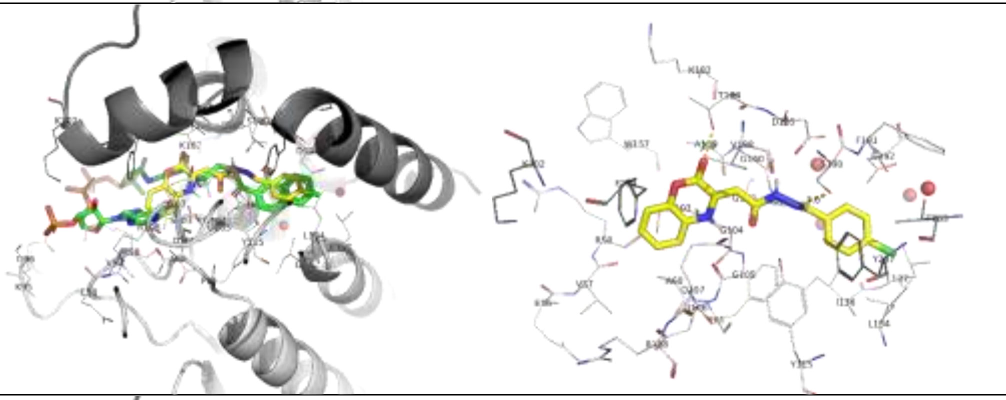
**S6**<br>Compound **1c**	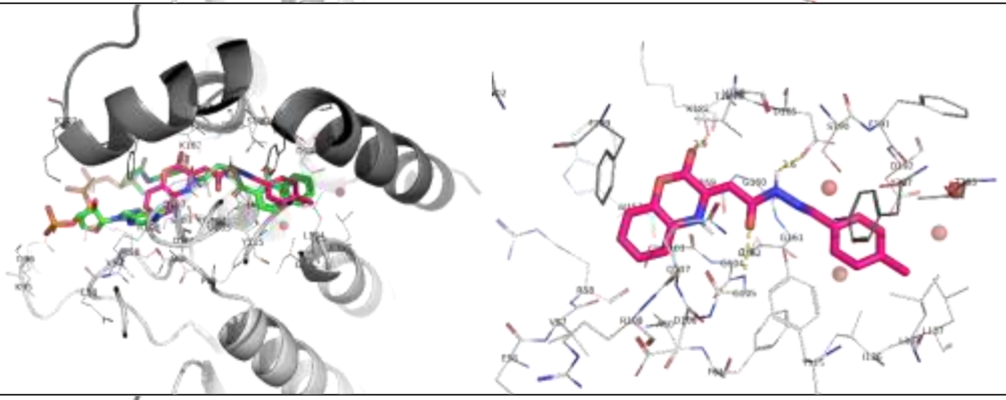
**S7**<br>Compound **1d**	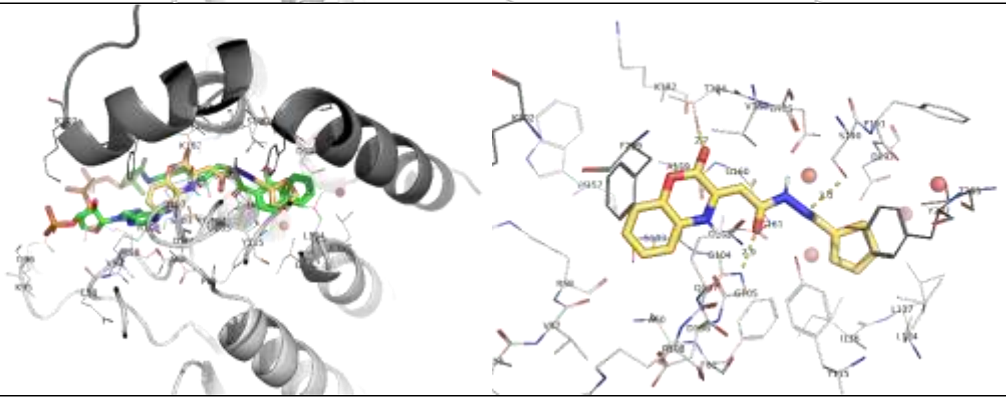

**S8**<br>Compound **1e**	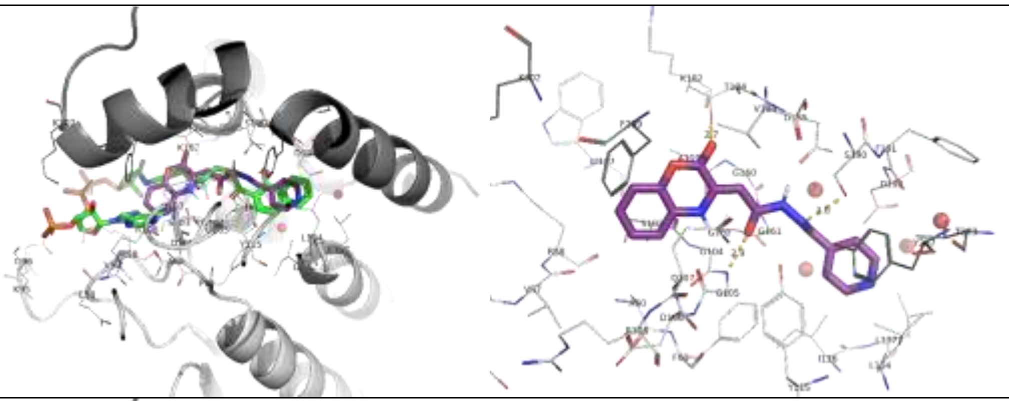
**S9**<br>Compound **1f**	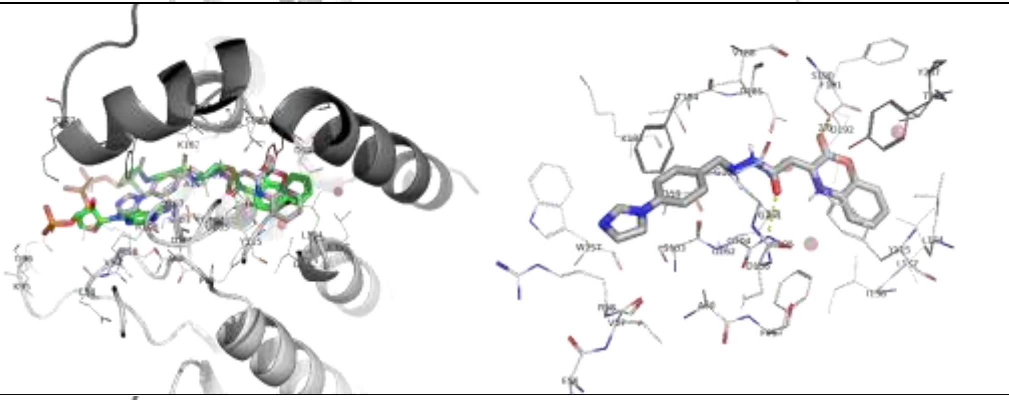
**S10**<br>Compound **1g**	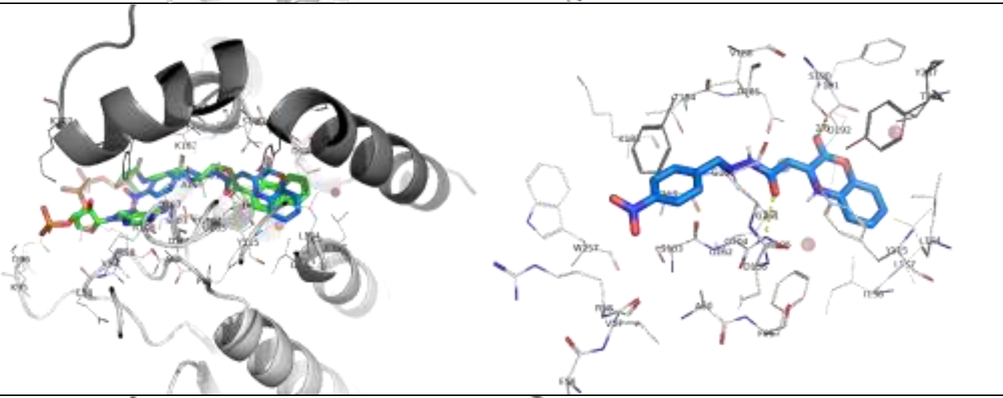
**S11**<br>Compound **1h**	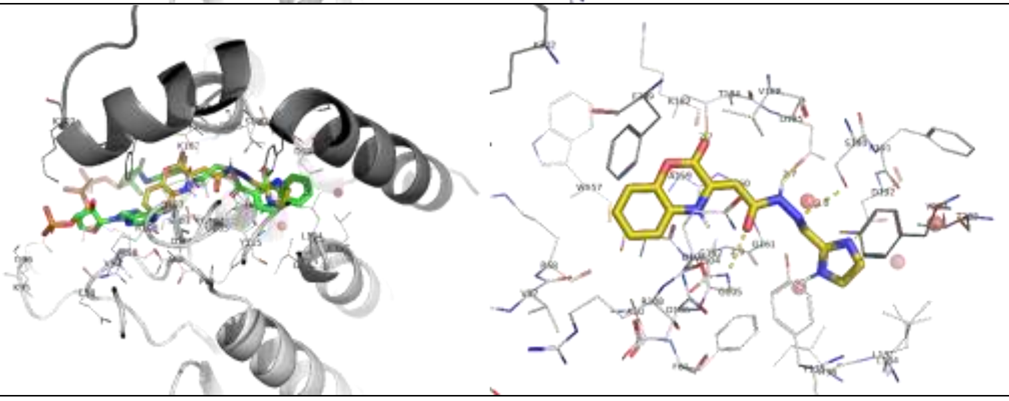

**S12**<br>Compound **1i**	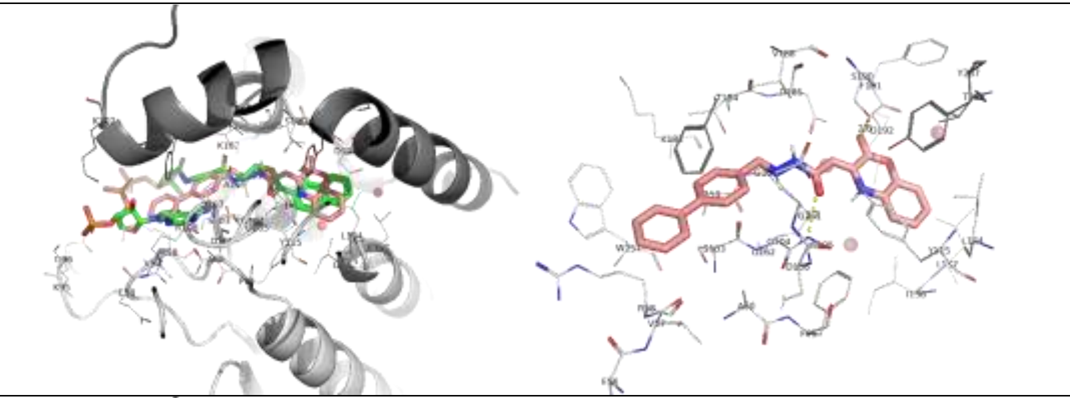
**S13**<br>Compound **1j**	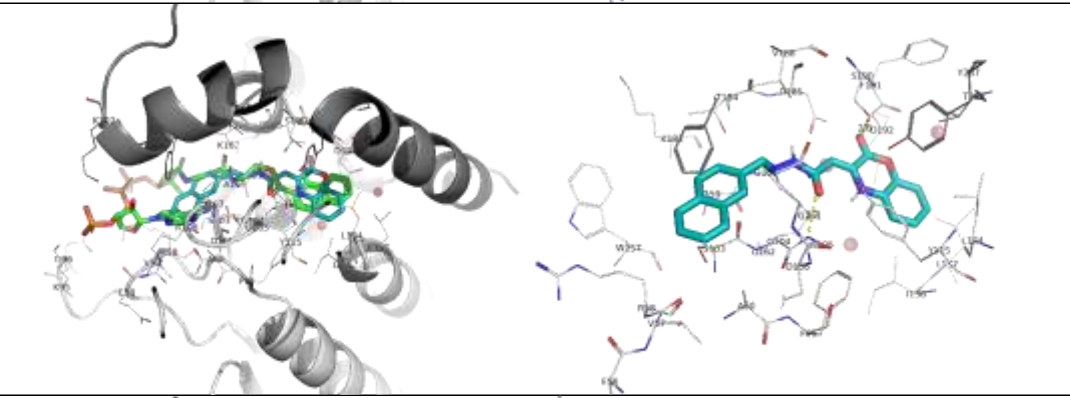
**S14**<br>Compound **1k**	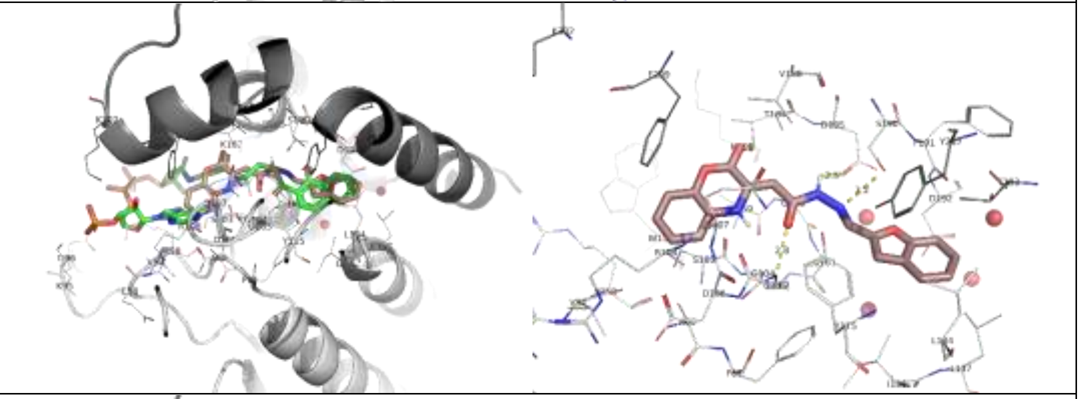
**S15**<br>Compound **1l**	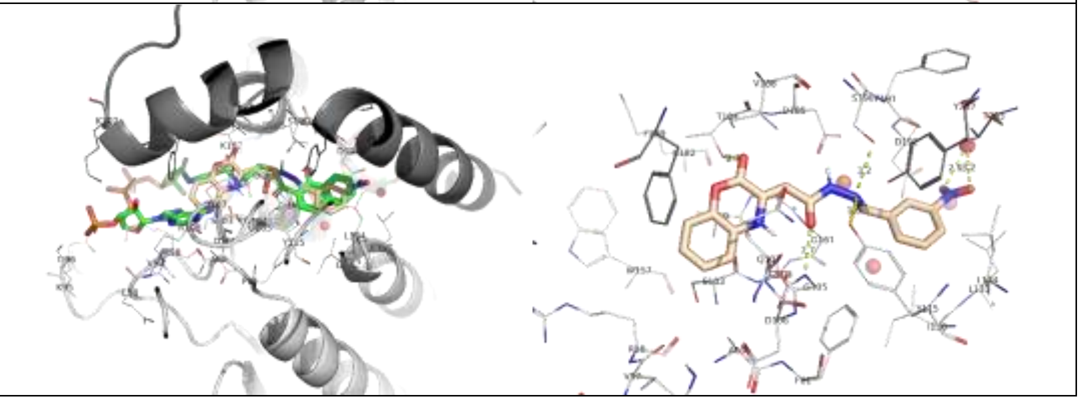

**S16**<br>Compound **1m**	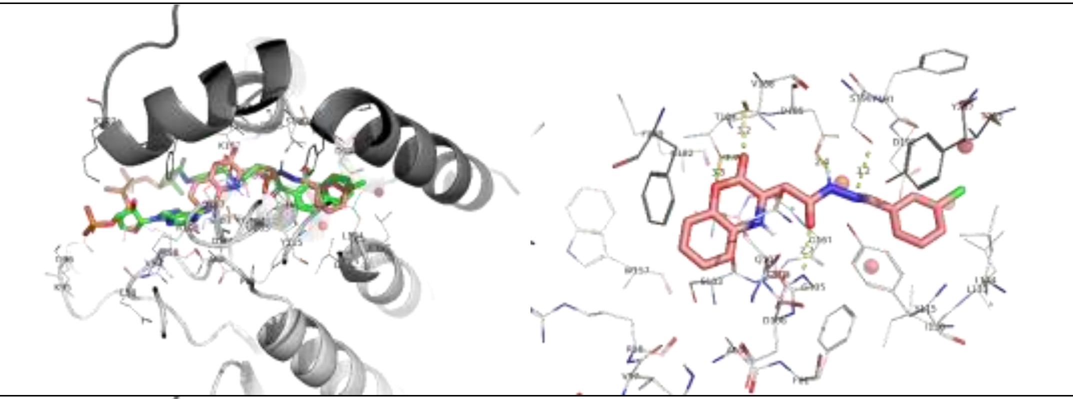
**S17**<br>Compound **1n**	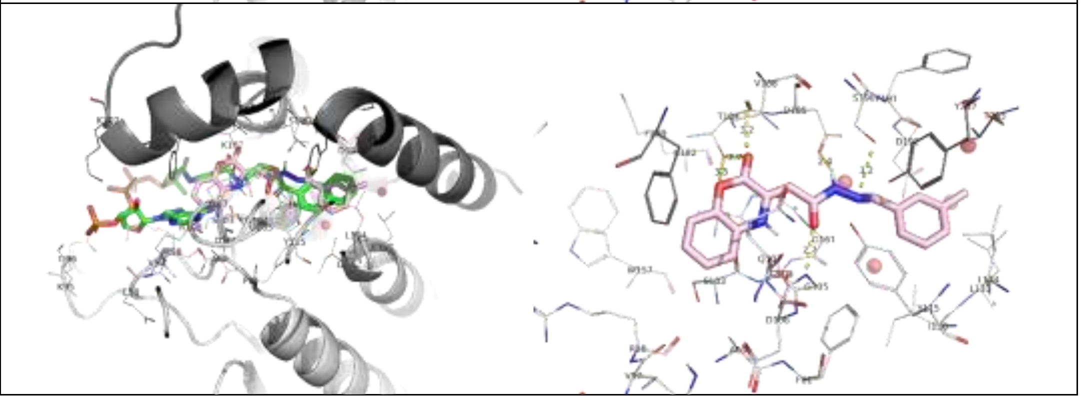

Figures S2. Docking figures for compounds **1a-n**.

Molecular Dynamics (MD)

4QIJ – reference compound 1 (Li *et al*. [reference 13])

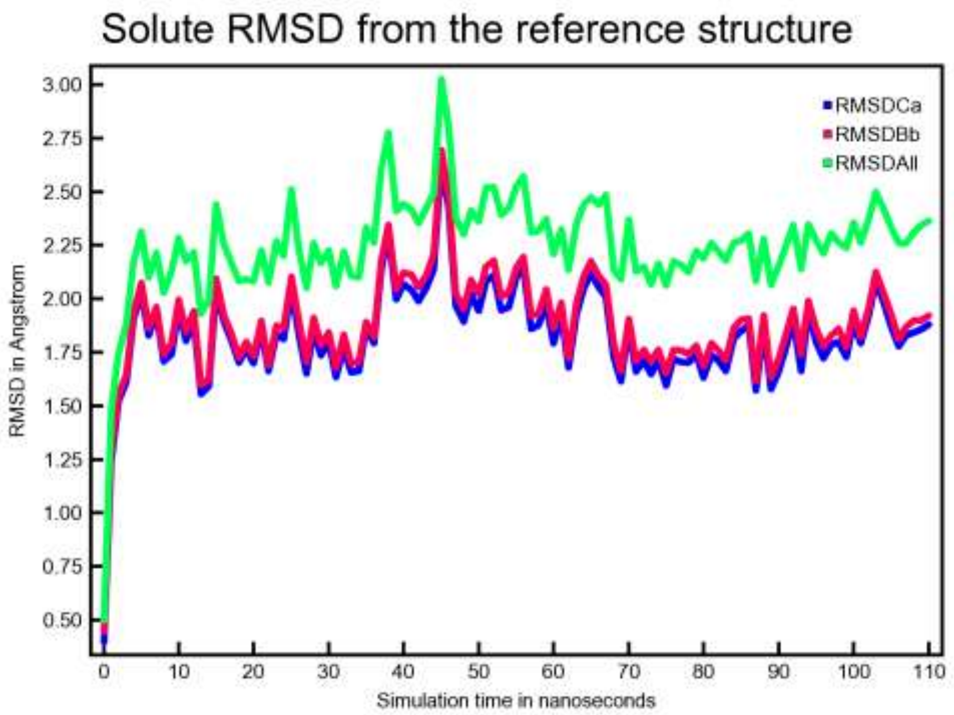

Figure S3. RMSD plot of the system with reference compound 1

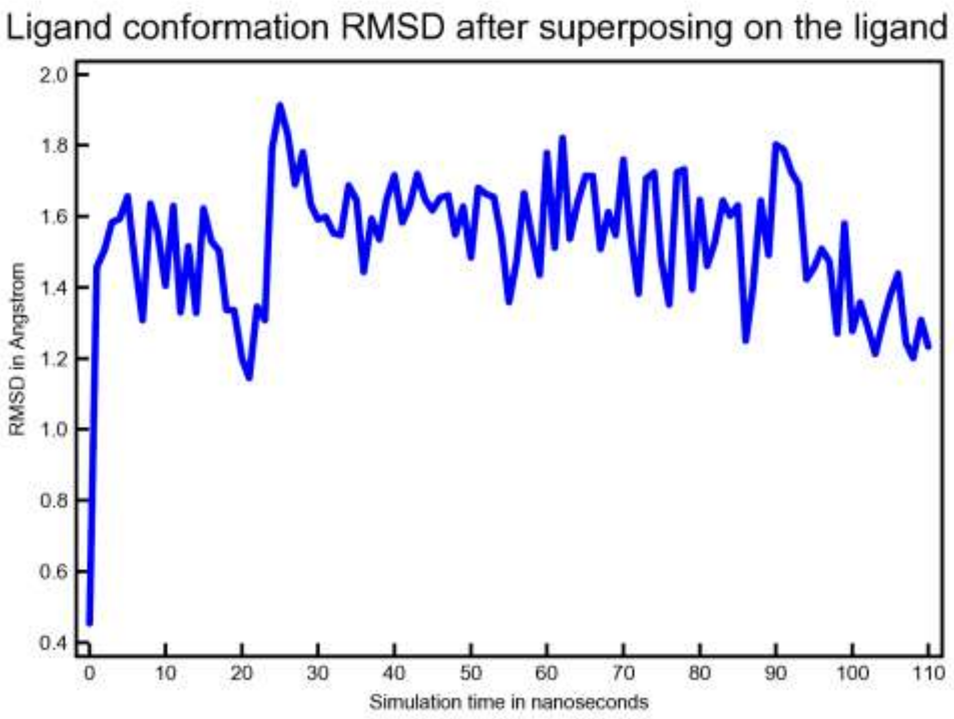

Figure S4. RMSD plot of reference compound 1.

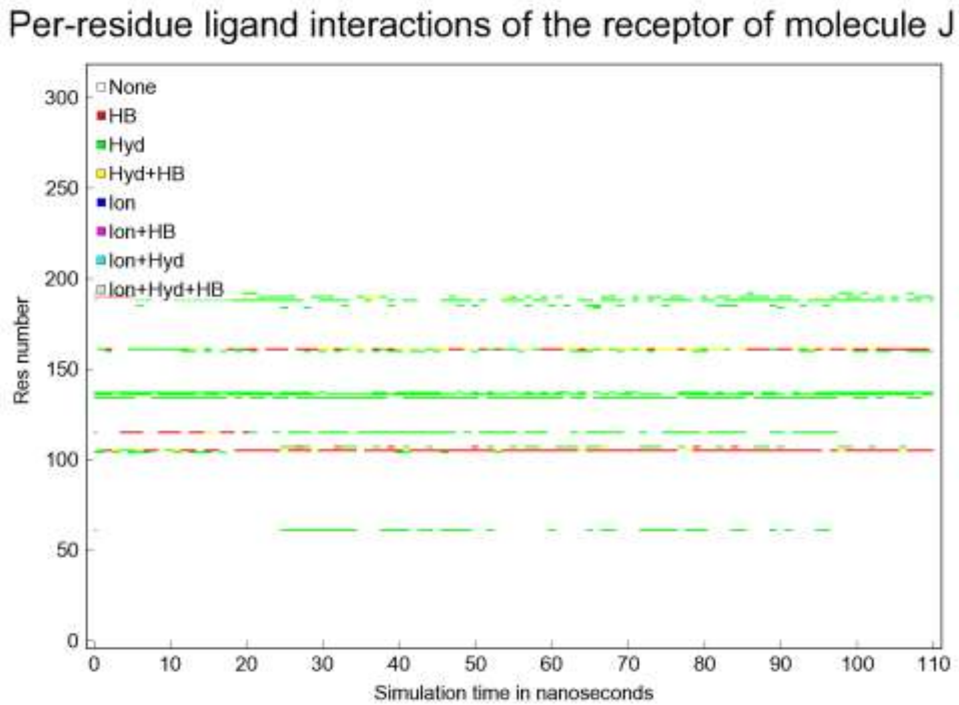

Figure S5. Interaction plot for reference compound 1.

4QIJ – **1a**

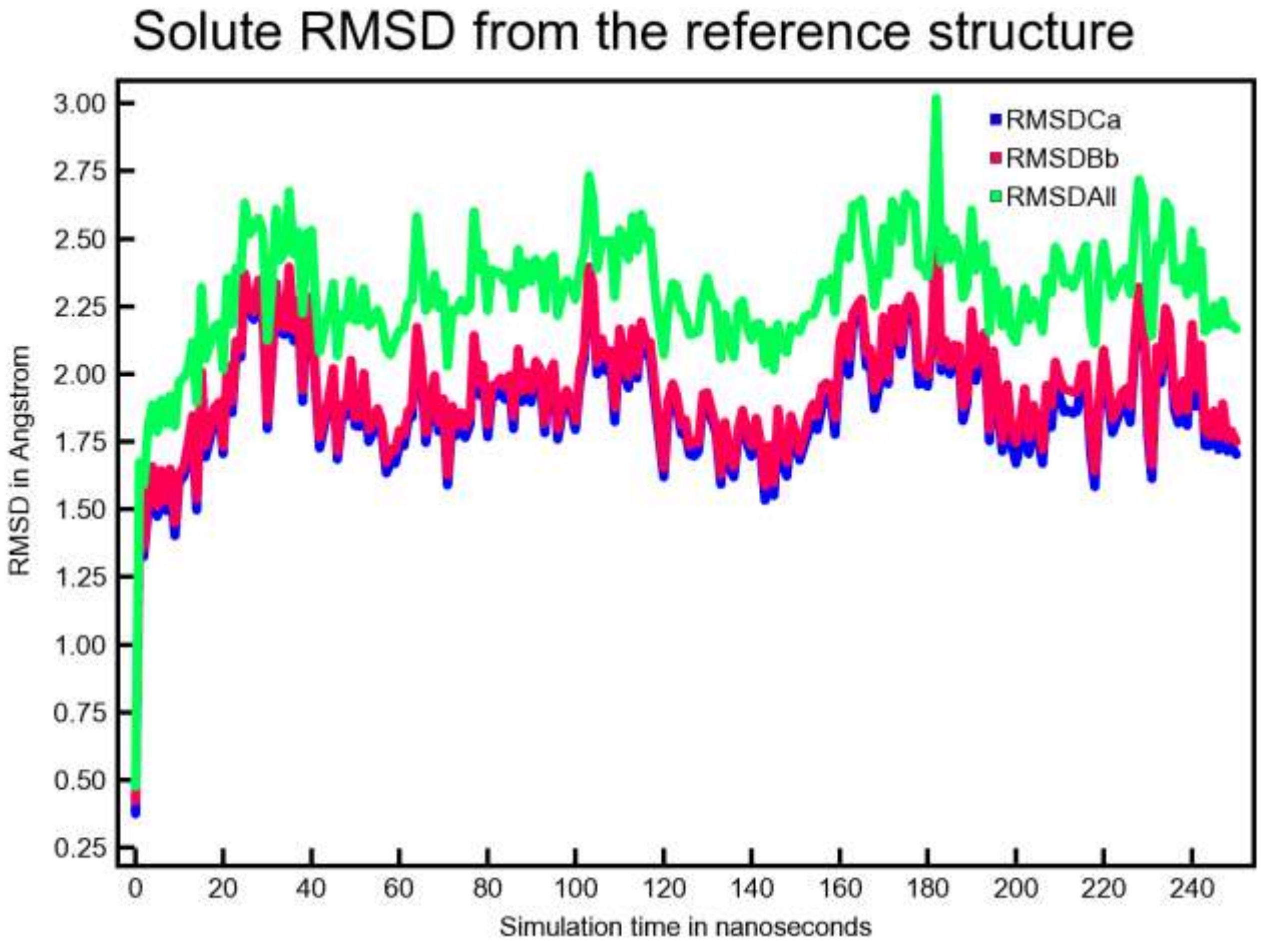

Figure S6. RMSD plot of the system with compound **1a**.

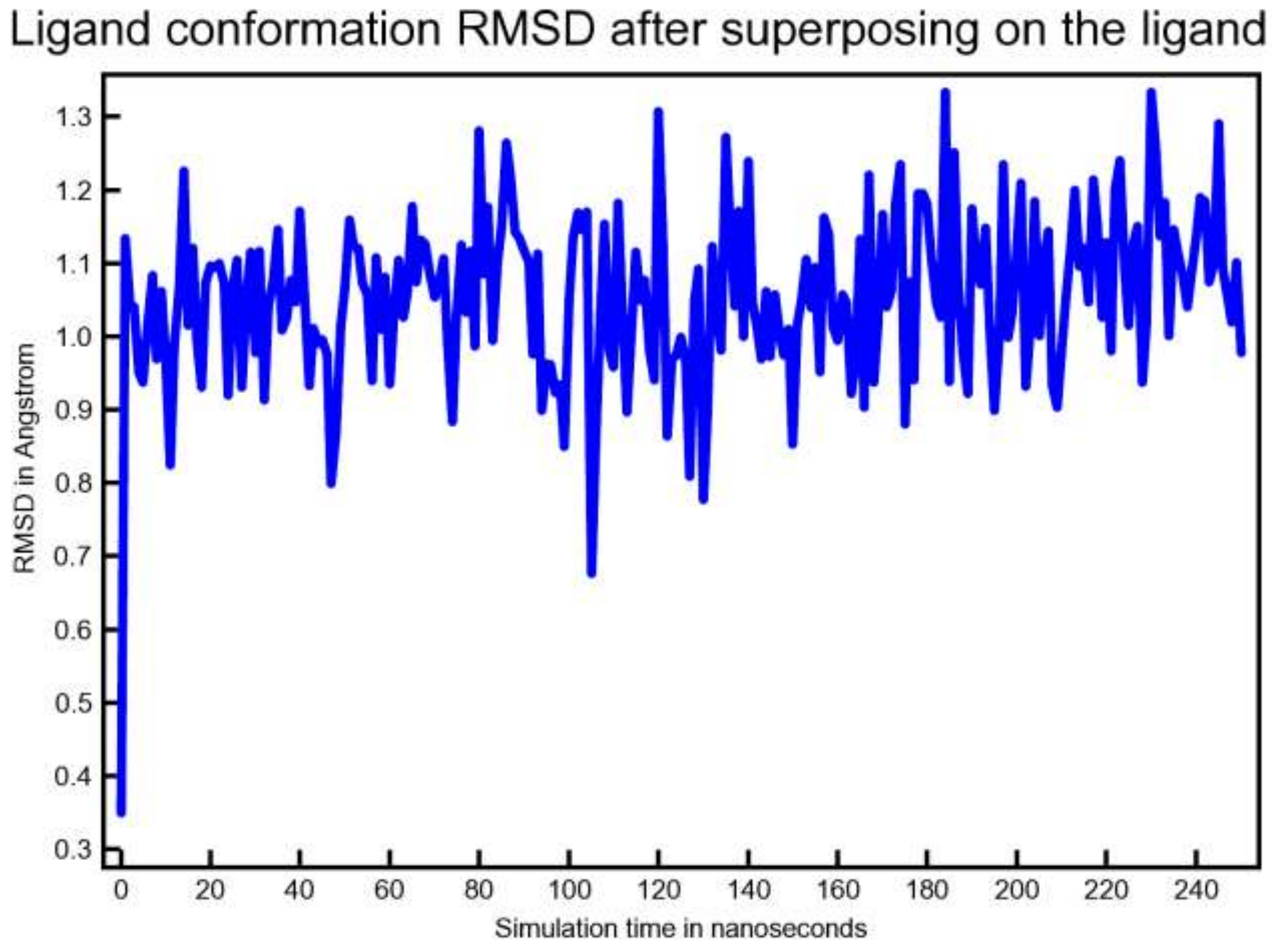

Figure S7. RMSD plot of compound **1a**.

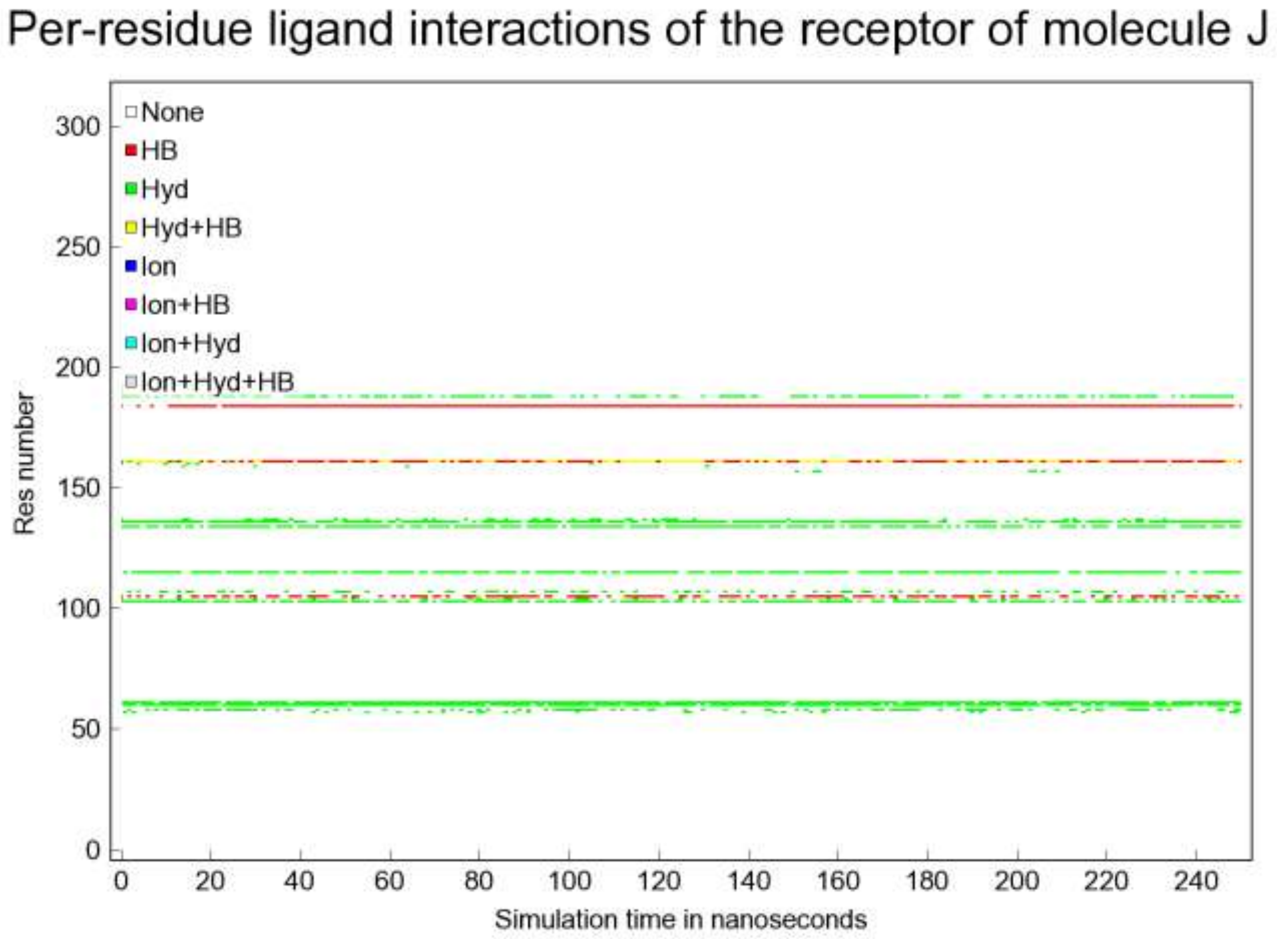

Figure S8. Interaction plot for compound **1a**.

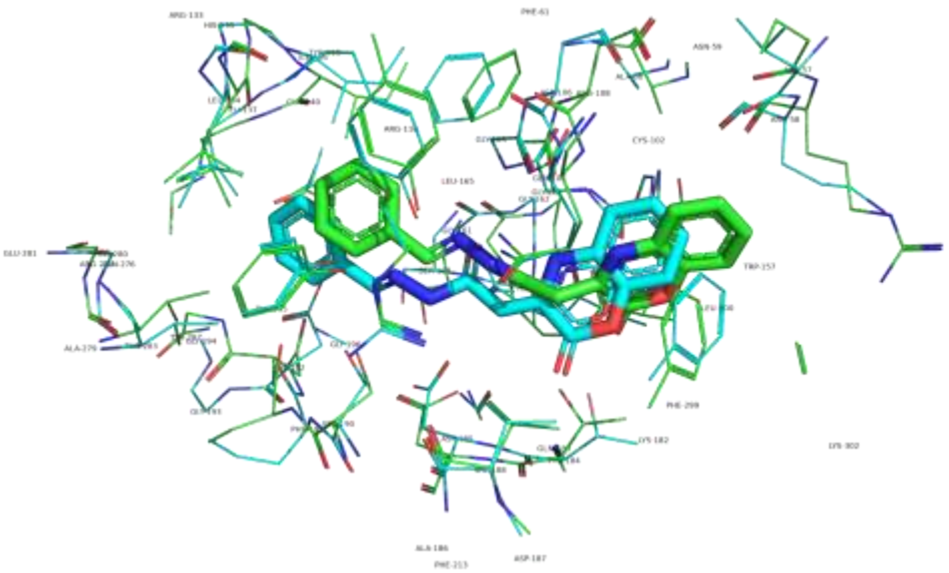

Figure S9. Comparison of first and last MD snaphots for compound **1a** indicating a similar binding pattern (First snapshot is colored magenta with small molecule in stick model and surrounding residues in labelled line model, last snapshot is colored green).

4QIJ – **1g**

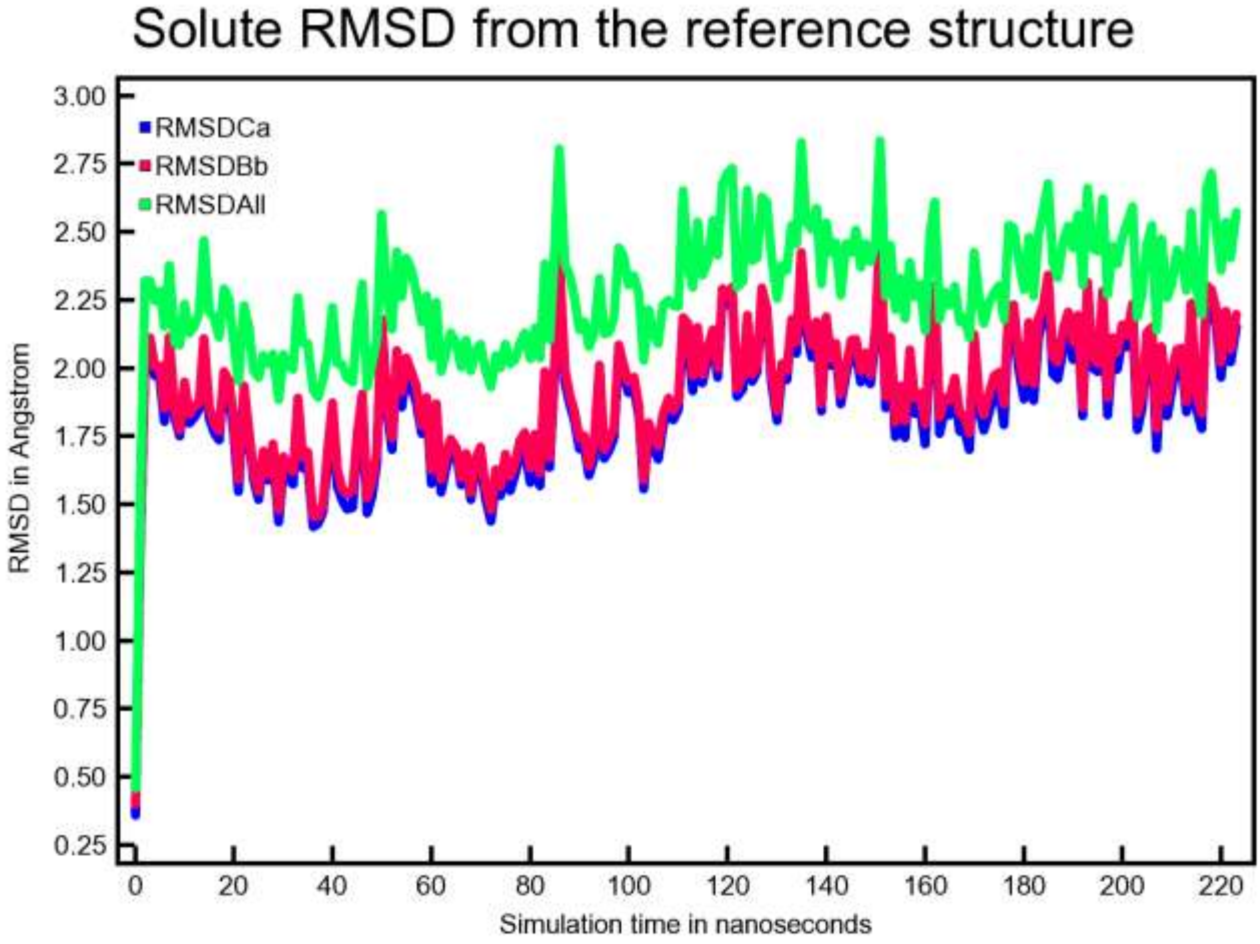

Figure S10. RMSD plot of the system with compound **1g**.

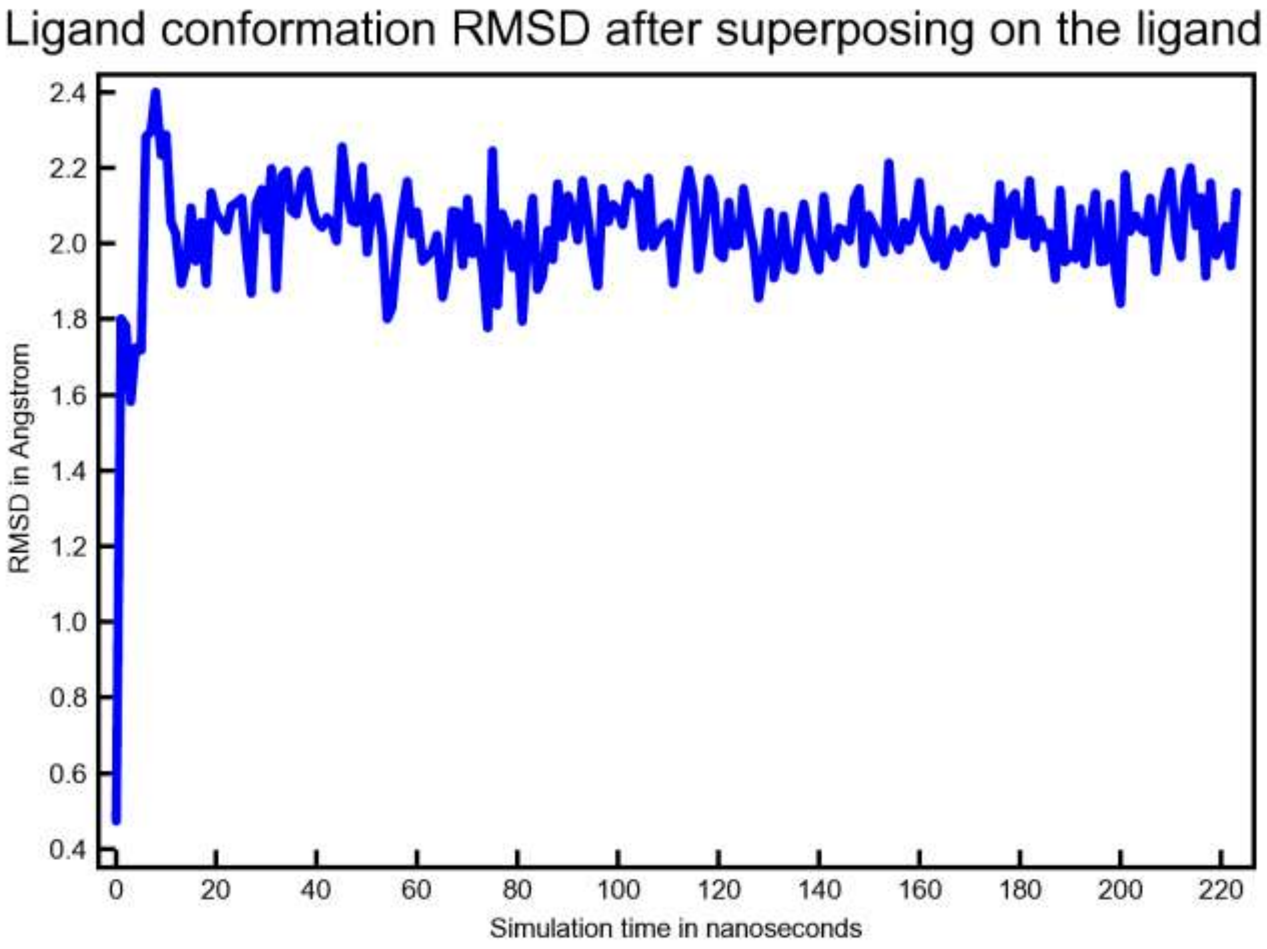

Figure S11. RMSD plot of compound **1g**.

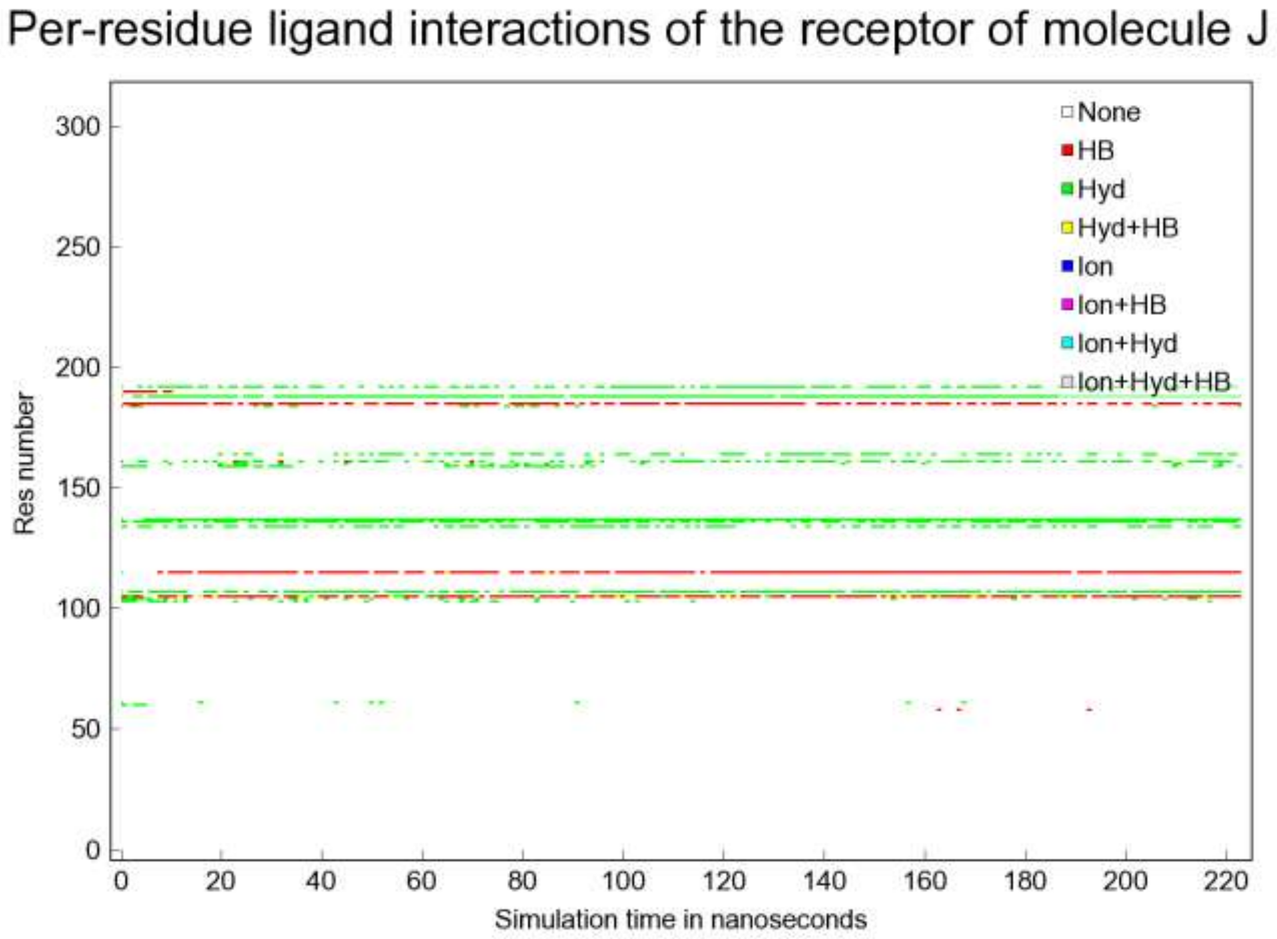

Figure S12. Interaction plot of compound **1g**.

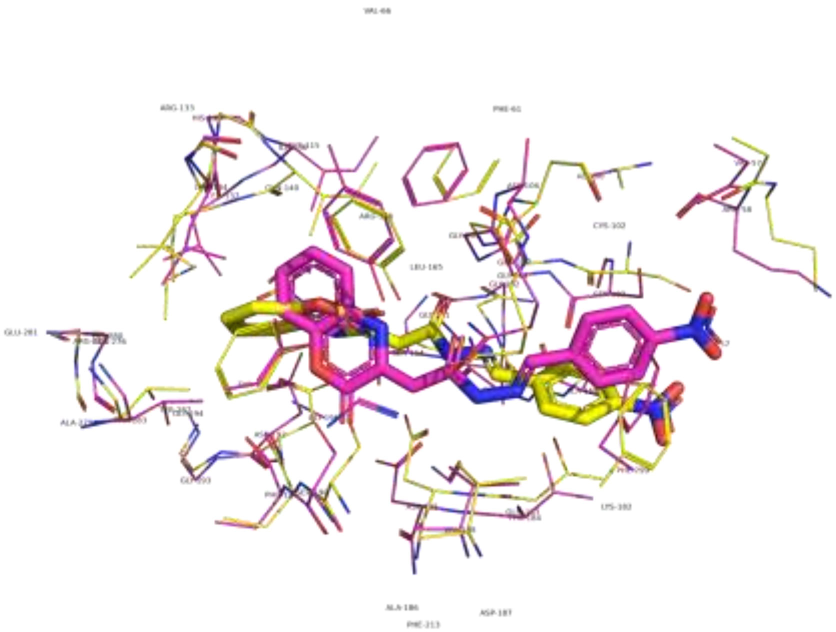

Figure S13. Comparison of first and last MD snaphots for compound **1g** indicating a similar binding pattern (First snapshot is colored purple with small molecule in stick model and surrounding residues in labelled line model, last snapshot is colored yellow).

4QIJ – **1m**

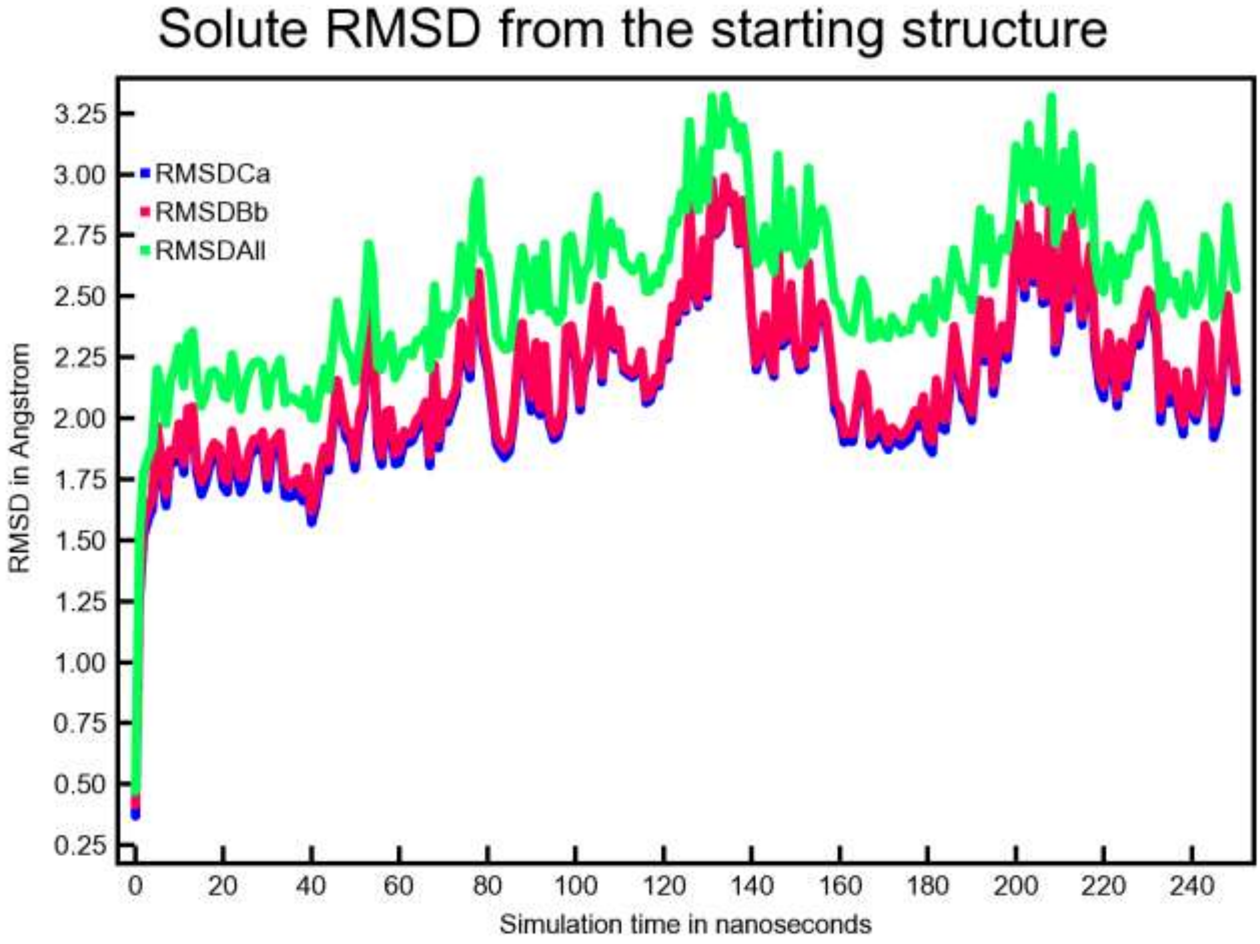

Figure S14. RMSD plot of the system with compound **1m**.

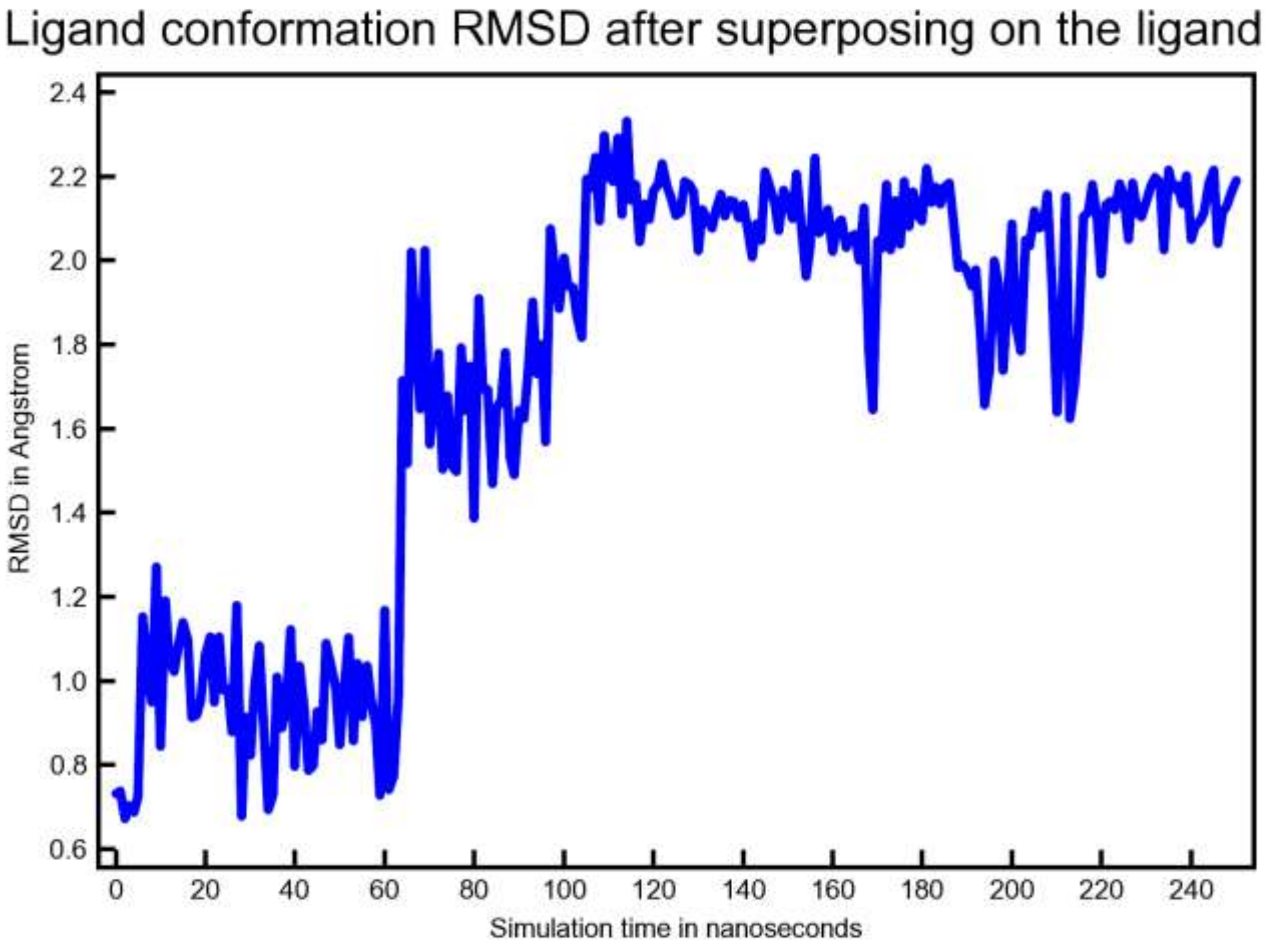

Figure S15. RMSD plot of compound **1m**.

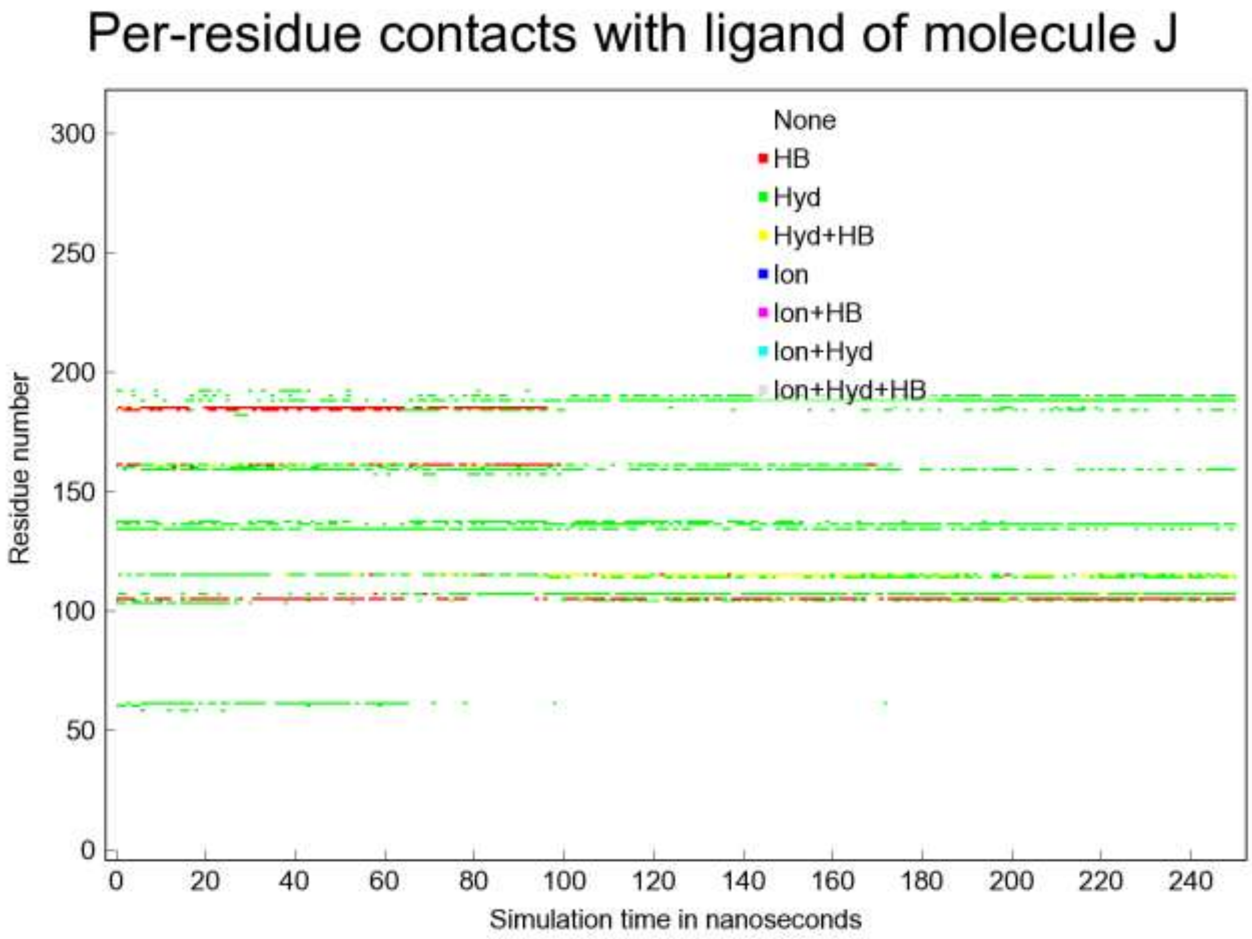

Figure S16. Interaction plot for compound **1m**.

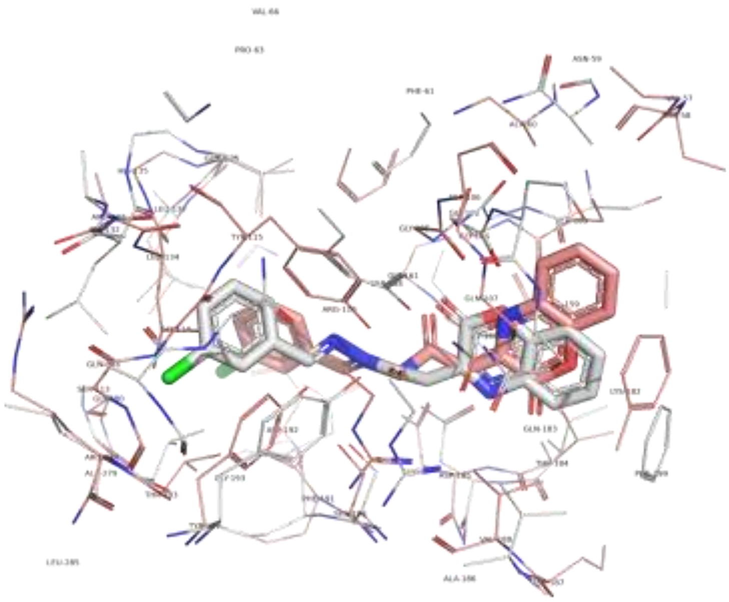

Figure S17. Comparison of first and last MD snaphots for compound **1m** indicating a similar binding pattern (First snapshot is colored pink with small molecule in stick model and surrounding residues in labelled line model, last snapshot is colored light-gray).
